# Metagenomic Shotgun Sequencing of Endocervical, Vaginal, and Rectal Samples among Fijian Women with and without Chlamydia trachomatis Reveals Disparate Microbial Populations and Function across Anatomic Sites: a Pilot Study

**DOI:** 10.1128/spectrum.00105-22

**Published:** 2022-05-17

**Authors:** Sankhya Bommana, Gracie Richards, Mike Kama, Reshma Kodimerla, Kenan Jijakli, Timothy D. Read, Deborah Dean

**Affiliations:** a Department of Pediatrics, University of California San Francisco, Oakland, California, USA; b Ministry of Health and Medical Services, Suva, Fiji; c Department of Medicine, Emory University School of Medicine, Atlanta, Georgia, USA; d Department of Medicine, University of California San Francisco, San Francisco, California, USA; e Department of Bioengineering, Joint Graduate Program, University of California San Francisco and University of California Berkeley, San Francisco, California, USA; f Bixby Center for Global Reproductive Health, University of California San Francisco, San Francisco, California, USA; g Benioff Center for Microbiome Medicine, University of California San Francisco, San Francisco, California, USA; University of Utah and ARUP Laboratories

**Keywords:** *Chlamydia trachomatis*, endocervical microbiome, metabolomics, metagenomic shotgun sequencing, rectal microbiome, sexually transmitted infections, vaginal microbiome, pathogenesis

## Abstract

Chlamydia trachomatis is a sexually transmitted pathogen and a global public health concern. Little is known about the microbial composition and function across endocervical, vaginal, and rectal microbiomes in the context of C. trachomatis infection. We evaluated the microbiomes of 10 age-matched high-risk Fijian women with and without C. trachomatis using metagenomic shotgun sequencing (MSS). Lactobacillus iners and Lactobacillus crispatus dominated the vagina and endocervix of uninfected women. Species often found in higher relative abundance in bacterial vaginosis (BV)—Mageeibacillus indolicus, *Prevotella* spp., *Sneathia* spp., Gardnerella vaginalis, and *Veillonellaceae* spp.—were dominant in C. trachomatis-infected women. This combination of BV pathogens was unique to Pacific Islanders compared to previously studied groups. The C. trachomatis-infected endocervix had a higher diversity of microbiota and microbial profiles that were somewhat different from those of the vagina. However, community state type III (CST-III) and CST-IV predominated, reflecting pathogenic microbiota regardless of C. trachomatis infection status. Rectal microbiomes were dominated by *Prevotella* and *Bacteroides*, although four women had unique microbiomes with *Gardnerella*, *Akkermansia*, *Bifidobacterium*, and *Brachyspira*. A high level of microbial similarity across microbiomes in two C. trachomatis-infected women suggested intragenitorectal transmission. A number of metabolic pathways in the endocervix, driven by BV pathogens and C. trachomatis to meet nutritional requirements for survival/growth, 5-fold higher than that in the vagina indicated that endocervical microbial functions are likely more diverse and complex than those in the vagina. Our novel findings provide the impetus for larger prospective studies to interrogate microbial/microbiome interactions that promote C. trachomatis infection and better define the unique genitorectal microbiomes of Pacific Islanders.

**IMPORTANCE** Chlamydia trachomatis is the primary cause of bacterial sexually transmitted infections worldwide, with a disturbing increase in annual rates. While there is a plethora of data on healthy and pathogenic vaginal microbiomes—defining microbial profiles and associations with sexually transmitted infections (STIs)—far fewer studies have similarly examined the endocervix or rectum. Further, vulnerable populations, such as Pacific Islanders, remain underrepresented in research. We investigated the microbial composition, structure, and function of these anatomic microbiomes using metagenomic shotgun sequencing among a Fijian cohort. We found, primarily among C. trachomatis-infected women, unique microbial profiles in endocervical, vaginal, and rectal microbiomes with an increased diversity and more complex microbial pathways in endocervical than vaginal microbiomes. Similarities in microbiome composition across sites for some women suggested intragenitorectal transmission. These novel insights into genitorectal microbiomes and their purported function require prospective studies to better define Pacific Islander microbiomes and microbial/microbiome interactions that promote C. trachomatis infection.

## INTRODUCTION

Chlamydia trachomatis sexually transmitted infections (STIs) pose a major public health concern due to escalating infections with global estimates of over 130 million cases per year (1). In the United States alone, 1.8 million C. trachomatis STIs were reported in 2019, a 19% increase from 2015 (2). However, Pacific Island Countries and Territories (PICT) have the highest prevalence of C. trachomatis STIs in the world (3–6), a likely consequence of the poor sensitivity and specificity of syndromic management (i.e., based on signs and symptoms) that have resulted in the undertreatment of C. trachomatis STIs (6, 7). Our recent study in Fiji found that C. trachomatis STIs occur at hyperendemic levels (30.5%) among adolescent and young adult women who are primarily asymptomatic (6). These untreated STIs can lead to severe inflammatory-related sequelae, such as pelvic inflammatory disease, tubal factor infertility, and preterm birth (8, 9).

C. trachomatis can influence the vaginal microbiomes causing diseases such as bacterial vaginosis (BV) (10–12). BV is also a risk factor for acquisition of C. trachomatis and other STIs such as Neisseria gonorrhoeae and HIV (13). Using 16S rRNA sequencing, previous studies have developed and utilized vaginal bacterial community state types (CSTs) to better understand the susceptibility to STIs. CST-I, CST-II, CST-III, and CST-V are dominated by Lactobacillus crispatus, Lactobacillus gasseri, Lactobacillus iners, or Lactobacillus jensenii, which produce lactic acid, bacteriocins, hydrogen peroxide, and other antimicrobial compounds to protect against STIs (14). CST-IV is deficient in *Lactobacillus* spp. with higher proportions of anaerobic bacteria such as Prevotella bivia, *Dialister*, Atopobium vaginae, Gardnerella vaginalis, Megasphaera elsdenii, *Peptoniphilus*, *Sneathia*, *Eggerthella*, *Aerococcus*, *Finegoldia*, and *Mobiluncus* (15, 16). This shift from a dominant *Lactobacillus* species population to a diverse microbial state, even when *L. iners* is abundant, is what can lead to acquisition of C. trachomatis and other STIs (13, 17).

The characterization of vaginal microbiomes has largely been based on 16S rRNA sequencing that provides genus-level taxonomic profiles of a community (10–12). This is less robust than metagenomic shotgun sequencing (MSS) where DNA is extracted from the entire microbial population, providing unbiased identification of all taxonomic communities of DNA organisms (e.g., fungi, bacteria, DNA viruses, and protozoa) to the species level and often the strain level. Metabolic pathway genes can also be ascertained directly using MSS. More recent studies have used MSS to elucidate vaginal microbial communities, providing examples of the power of this approach (18–20). Although interrogating vaginal microbial communities is important in understanding healthy versus pathogenic microbial environments and risk for STIs, evaluating the endocervix may be more pivotal, as it is the actual site of C. trachomatis infection, not the vagina. Very few studies have examined cervical microbiomes used 16S rRNA sequencing in the presence or absence of C. trachomatis without comparison to vaginal microbiota (21–24). Only one study to date has evaluated the interrelationship of these microbiomes, again using 16S rRNA sequencing, with a focus on adolescents in South Africa (11). Differences in microbial taxa and diversity were evident between sites, but no significant microbial associations with C. trachomatis were found (11).

Numerous studies have now shown that C. trachomatis rectal infections outnumber those in the female urogenital tract. In a Dutch study, over 70% of women with urogenital C. trachomatis also had rectal C. trachomatis (25). There are also reports of rectal C. trachomatis infections in the absence of urogenital STIs, suggesting a silent reservoir for transmission (25, 26). Additionally, men who have sex with men (MSM) and women also have high rates of C. trachomatis rectal infections (25, 27, 28). Since these infections require a longer treatment regimen of 7 to 21 days compared to a single dose of azithromycin for uncomplicated urogenital infections, there is an increased risk of transmission from ineffective therapy (29, 30). Further, we know relatively little about the interactions of rectal, vaginal, and endocervical microbiomes in transmission and maintenance of infection and disease. Most of what we know about the impact of STIs on rectal microbiomes is from HIV-1 studies (31–34). Currently, few studies have addressed C. trachomatis infections and their effect on rectal microbiota (22, 35).

While many studies have evaluated vaginal and gut microbiomes in various populations, these studies have primarily been based on 16S rRNA sequencing (36), and none have explored the microbial composition of the endocervix, vagina, and rectum within or across women or in the context of C. trachomatis infection. Here, using a small cohort of age-matched Fijian women with and without C. trachomatis infection, we employed MSS to evaluate the microbial composition and relative abundance of microbiota at each site, including concordance/discordance of endocervical, vaginal, and rectal microbiota and the extent and functional significance of microbial sharing across anatomic sites.

## RESULTS

### Patient and sample characteristics.

**FIG 1:**
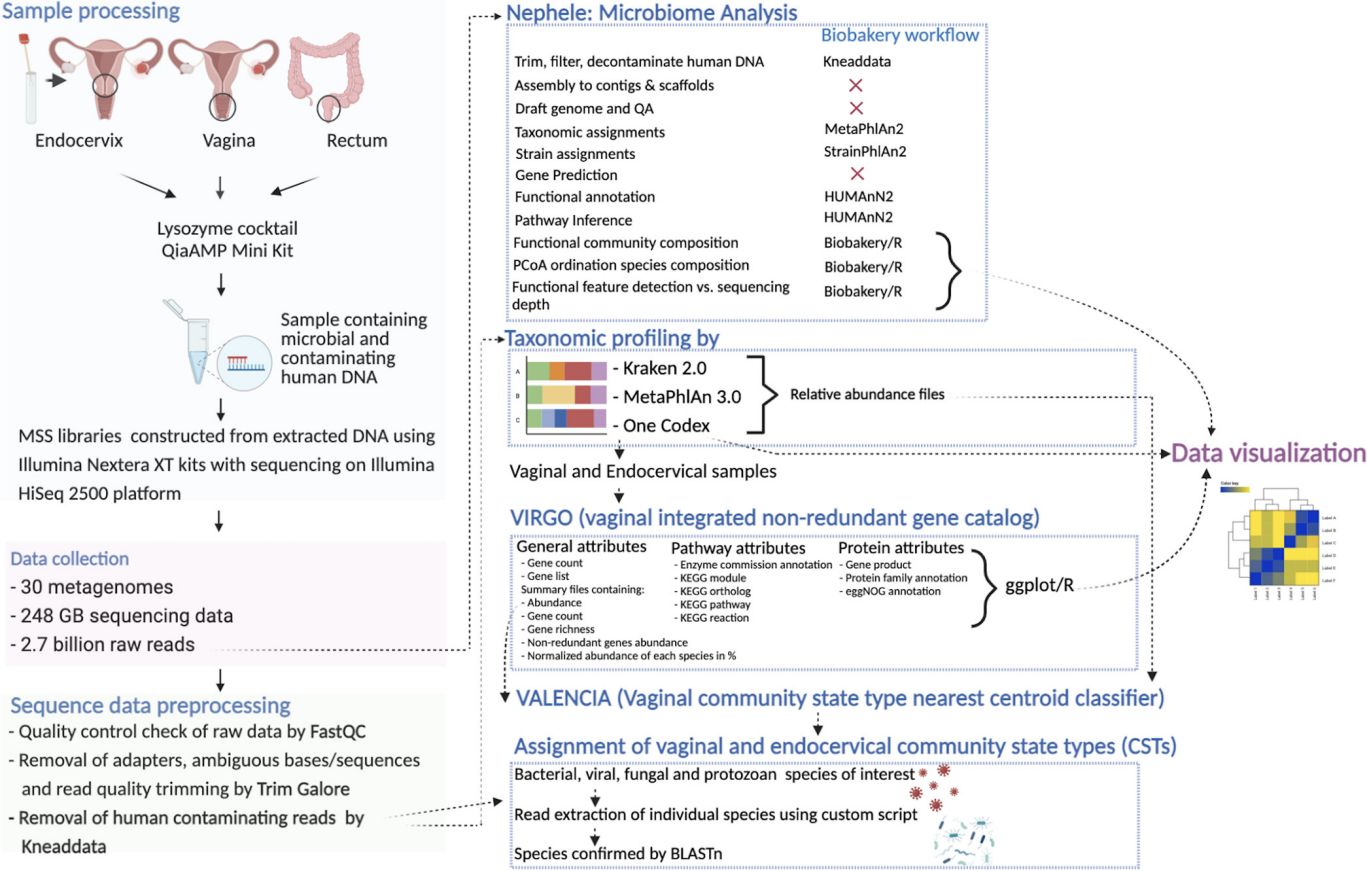
Pipeline for clinical sample data generation, processing, and integration for the characterization of the endocervical, vaginal, and rectal microbiomes.

Table 1 shows the characteristics of the age-matched cohort. The *omp*A genotype, which identifies the C. trachomatis strain, was derived from the respective C. trachomatis genome sequences (Tim Read, personal communication) (37). The C. trachomatis and beta-actin genome copy numbers and chlamydial load for each anatomic site are also shown. The chlamydial mean load was significantly higher in rectal (30.36 ± 20.71) than vaginal sites (1.72 ± 1.77; *P* = 0.0105; one-way analysis of variance [ANOVA]) and in rectal than endocervical sites (0.15 ± 0.05; *P* = 0.0108; one-way ANOVA) (Table 1) in samples from our cohort. Sample 362R was an exception to this, with a higher load detected in the vagina. Sexually transmitted and genitorectal pathogens were considered to be present in the samples if they were detected at a ratio of at least one read per million (RPM; see Materials and Methods) and if the reads corresponded to at least three different regions of the genome. Pathogen reads were confirmed by BLASTn as described by Babiker et al. (see Table S1 in the supplemental material) (38). While all vaginal and endocervical samples were negative for N. gonorrhoeae by the Xpert CT/NG test and for human papillomavirus (HPV) by pap smear, respectively, MSS detected five women with rectal N. gonorrhoeae infections and two women with HPV infections where the aforementioned tests were not available (Table 1; Table S1). *Candida* was detected by wet prep for sample 319V, while MSS additionally identified *Candida* in one vaginal and one rectal sample. Six women had BV based on Amsel criteria from the parent study; four of these women were infected with C. trachomatis, Mycoplasma genitalium, or HPV. M. genitalium was detected based solely on MSS reads (Table S1). All reads were extracted and confirmed by BLASTn (Fig. 1).

**TABLE 1:** Characteristics of age-matched Pacific Islander women of iTaukei ethnicity with and without C. trachomatis infection, including *omp*A genotype and load^*a*^

No.	ID^*b*^	Status of:						*omp*A genotype	Age in yrs^*e*^	C. trachomatis *omp*A (genomes/μL)	Beta-actin (genomes/μL)	Load^*f*^
		C. trachomatis status	BV^*c*^	N. gonorrhoeae	*Candida*	HPV/type^*d*^	M. genitalium					
1	72V	+	+	−	−	−	−	G	32	2,107.5	482.5	4.36
2	72R	+	−	−	−	−	***+***	G		1,664	41.29	40.29
3	72C	+	−	−	−	−	−	G		349.2	2,495.27	0.13
4	98V	+	+	−	−	−	−	G	22	344	3,947.59	0.08
5	98R	+	−	***+***	−	−	***+***	G		31,549	555.29	56.81
6	98C	+	−	−	−	−	−	G		434.4	1,809.35	0.24
7	107V	+	+	−	−	−	***+***	Ja	29	2,218	1,301.54	1.70
8	107R	+	−	−	−	−	***+***	Ja		18,377	708.58	25.93
9	107C	+	−	−	−	−	***+***	Ja		21.2	815.62	0.14
10	192V	+	−	−	−	***+/7***	***+***	Ja	25	328	2,383.45	0.13
11	192R	+	−	***+***	−	***+/53, 54***	***+***	Ja		43,905	1,550.32	28.31
12	192C	+	−	−	−	−	***+***	Ja		275.1	NA	NA
13	362V	+	−	−	−	−	−	F	34	1,933.7	829.48	2.33
14	362R	+	−	−	***+***	−	***+***	F		576.3	1,236.35	0.46
15	362C	+	−	−	−	−	−	F		136.4	1,067.45	0.12
16	57V	−	+	−	−	−	−	NA	32	NA	NA	NA
17	57R	−	−	−	−	−	***+***	NA		NA	NA	NA
18	57C	−	−	−	−	−	***+***	NA		NA	NA	NA
19	35V	−	+	−	−	***+/6b, 16***	−	NA	21	NA	NA	NA
20	35R	−	−	***+***	−	***+/16, 26***	***+***	NA		NA	NA	NA
21	35C	−	−	−	−	***+/16***	−	NA		NA	NA	NA
22	121V	−	−	−	***+***	−	−	NA	29	NA	NA	NA
23	121R	−	−	−	−	−	***+***	NA		NA	NA	NA
24	121C	−	−	−	−	−	−	NA		NA	NA	NA
25	30V	−	+	−	−	−	−	NA	25	NA	NA	NA
26	30R	−	−	***+***	−	−	***+***	NA		NA	NA	NA
27	30C	−	−	−	−	−	−	NA		NA	NA	NA
28	319V	−	−	−	+	−	−	NA	34	NA	NA	NA
29	319R	−	−	***+***	−	−	***+***	NA		NA	NA	NA
30	319C	−	−	−	−	−	−	NA		NA	NA	NA

a NA, not available.

b V, vagina; R, rectum; C, endocervix.

c BV, bacterial vaginosis determined by Amsel criteria.

d + sign refers to the sample being positive (for the respective organism or disease listed at the top of the column), and the − sign refers to the sample being positive (for the respective organism or disease listed at the top of the column); bold italic ***+***, detected by metagenomic shotgun sequencing (MSS).

e Mean age, 28 years for cases and matched controls.

f Load, *omp*A copy no./beta-actin copy no.; mean vaginal load, 1.72; mean endocervical load, 0.16; mean rectal load, 30.36; the letters G, Ja and F refer to the *omp*A genotype based on the sequence of the *omp*A gene, which is the gold standard for genotyping *C. trachomatis* strains.

### MSS.

The 30 metagenomes yielded a total of ~2.8 billion raw reads, of which 1.1 billion (39%) were identified as human and removed (Table S2). The reads per sample ranged from 60.66 million to 117.44 million. Comparing human DNA contamination across anatomic sites, we found that there was a significant difference in human to raw read ratio in the rectum versus in the vagina (*P* < 0.0001, one-way ANOVA) and in the rectum versus in the endocervix (*P* < 0.0001, one-way ANOVA), with a higher mean proportion of human sequence reads in the rectum of 66% (range of 32.4% to 87.6%) than in the endocervix, 24.5%, and in the vagina, 23.6% (range of 8.9% to 48.5%) (Table S2, Fig. S1).

### Endocervical, vaginal, and rectal microbiome alpha and beta diversity.

**FIG 2:**
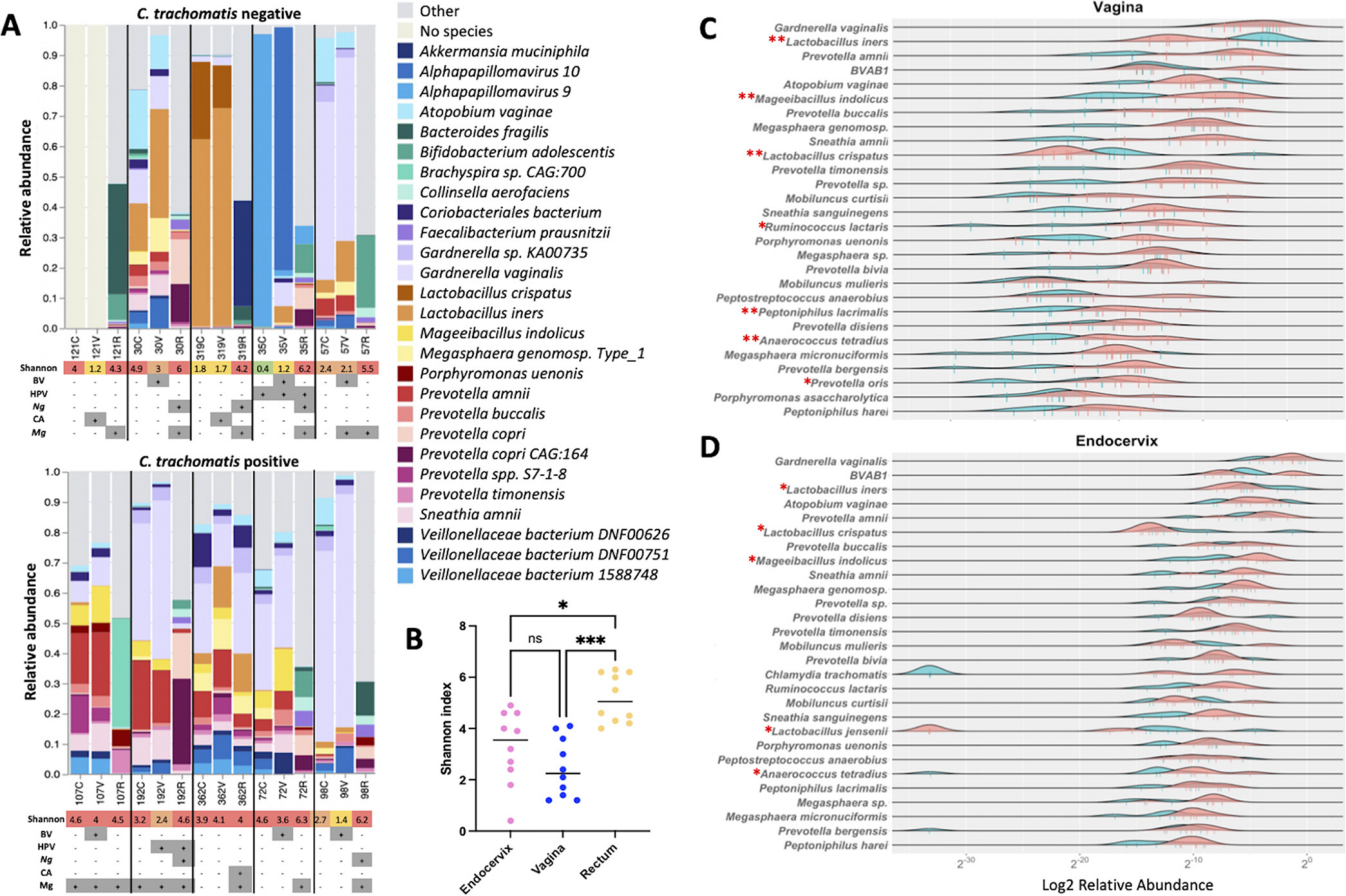
Taxonomic composition of the endocervical, vaginal, and rectal microbiota. (A) The relative abundance bar plots represent the top 28 species within the microbial community structure of the C. trachomatis-positive and -negative women. The colored boxes below indicate the Shannon index. The grid below the Shannon index indicates the presence or absence of bacterial vaginosis (BV), human papillomavirus (HPV), Neisseria gonorrhoeae (*Ng*), Candida albicans (CA), and/or Mycoplasma genitalium (Mg). (B) Anatomic site-specific microbial Shannon diversity for the endocervix, vagina, and rectum. Rectal samples had a statistically significant difference in Shannon diversity compared with endocervical (*, *P* < 0.05) and vaginal (***, *P* < 0.0005) samples. The difference for endocervix versus vagina was not significant (ns) as measured by Kruskal-Wallis test and Dunn’s multiple comparisons. The top 28 species in the vaginal (C) and endocervical (D) microbiomes with the most unique abundant gene content in VIRGO are plotted. Density curves for each species compare the distribution of the relative abundance of reads for the C. trachomatis-positive (in red) and -negative women (in green), and the vertical bars represent the individual data points. Significant differences in relative abundance of species between positive and negative women are shown (*, *P* < 0.05; **, *P* < 0.01). Statistical significance was determined using two-sample Wilcox test between the C. trachomatis-positive and -negative samples. The *x* axis represents the log_2_ of the ratio of the unique gene content of a species divided by the gene content of the entire community.

There were significant differences in alpha diversity across all three anatomic sites (Fig. 2A and B; *P* = 0.0005; Kruskal-Wallis test). In pairwise comparisons, there was a significantly higher diversity in the rectum than in the vagina (*P* < 0.0005) and the endocervix (*P* < 0.05). Similarly, the diversity was higher in the endocervix than in the vagina for both C. trachomatis-positive (mean 3.8 versus 3.1) and C. trachomatis-negative (mean 2.7 versus 1.8) women, but this was not statistically significant. While there were no significant differences between C. trachomatis-negative and -positive microbiomes across all anatomic sites, there was a trend of an overall higher diversity for C. trachomatis-positive microbiomes (mean of 4.07 versus 3.26).

The relative abundance of the top 28 taxa (a limit set by One Codex) identified in the endocervix, vagina, and rectum is shown in Fig. 2A. Endocervical and vaginal microbial communities in C. trachomatis-negative women were dominated mostly by *L. iners* and L. crispatus, with an exception for 35C and 35V, which were dominated by *Alphapapillomavirus* 9 and 10. Sample 121 had no species at high (>25% of classified reads) or medium abundance (>5 to 25% of classified reads), with all organisms occurring at low abundance within 1 to 5%; there was insufficient abundance for representation in either site. C. trachomatis-positive women had microbiomes comprised primarily of G. vaginalis, Mageeibacillus indolicus, *Prevotella* spp., and Sneathia amnii (Fig. 2A). The relative abundance in the vagina of *L. iners* and L. crispatus was significantly higher in C. trachomatis-negative than in C. trachomatis-positive women (*P* < 0.01), whereas the relative abundance of M. indolicus (*P* < 0.01), Ruminococcus lactaris (*P* < 0.05), Peptoniphilus lacrimalis (*P* < 0.01), Anaerococcus tetradius (*P* < 0.01), and Prevotella oris (*P* < 0.05) was significantly higher in C. trachomatis-positive than in C. trachomatis-negative women (Fig. 2C). In the endocervix, the relative abundance of *L. iners*, L. crispatus, and L. jensenii (*P* < 0.05) was significantly higher in C. trachomatis-negative than in C. trachomatis-positive women, whereas the relative abundance of *M. indolicus* and A. tetradius (*P* < 0.05) was significantly higher in C. trachomatis-positive than in C. trachomatis-negative women (Fig. 2D). The bacterial species in the endocervix and vagina mentioned above that were significantly higher in abundance in C. trachomatis-related pathogenic microbiota were all previously found to be in higher abundance in BV (39–44). All endocervical and vaginal microbial species for which the relative abundance was significantly altered between C. trachomatis-positive and -negative samples are shown in Fig. S2A and B.

**FIG 3:**
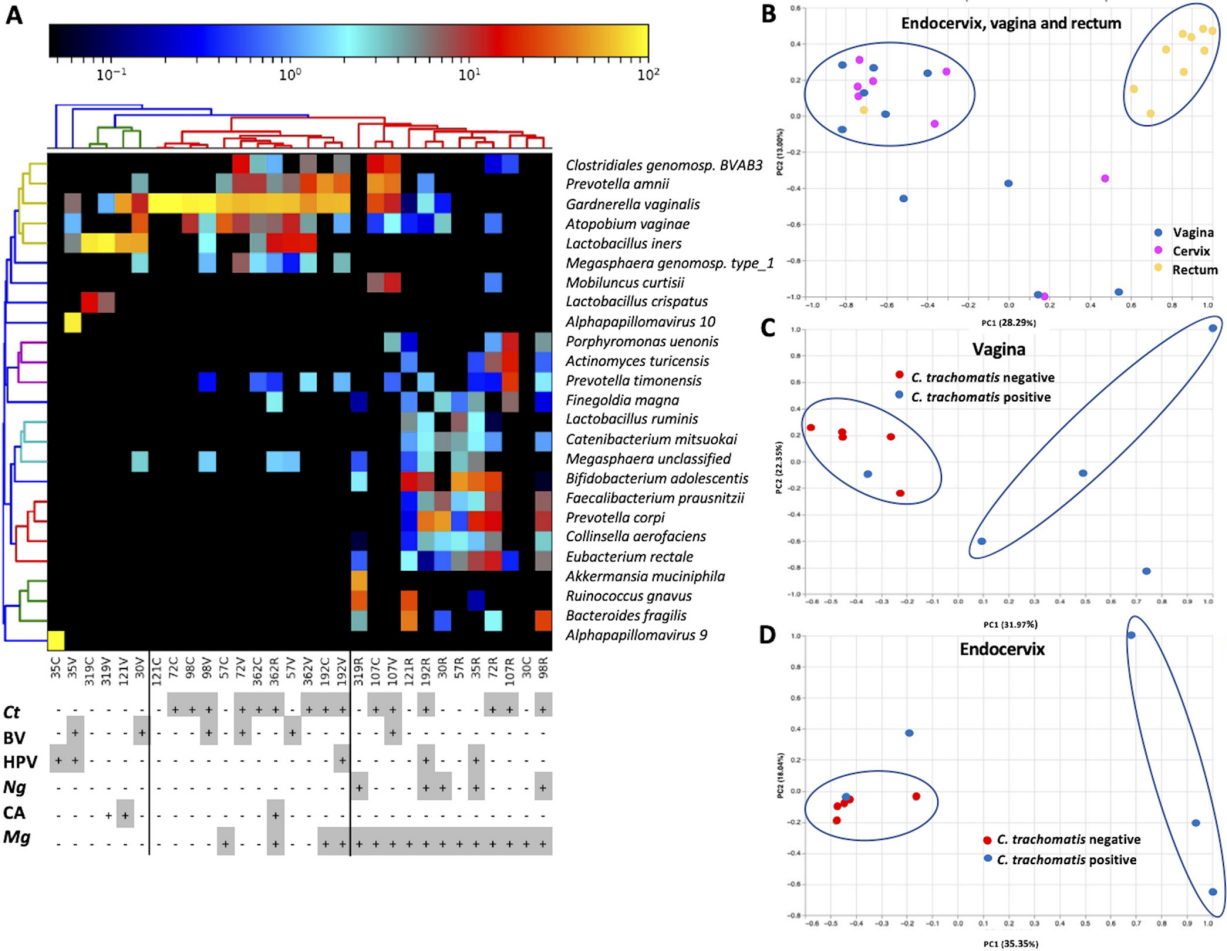
Bray-Curtis hierarchical clustering of endocervical, vaginal, and rectal samples and principal coordinates analysis (PCoA) plot of samples from all three anatomic sites. (A) Clustering is based on species composition and percent abundance of microbial communities. Color key of percent abundance is indicated on the top: scale toward black (0) and yellow (100) indicates lower and higher percent abundance, respectively. Rows represent taxa, and columns represent samples. Grid below heatmap indicates the presence or absence of C. trachomatis (*Ct*), bacterial vaginosis (BV), human papillomavirus (HPV), Neisseria gonorrhoeae (*Ng*), *Candida* (CA), and Mycoplasma genitalium (*Mg*). (B) Principal coordinates analysis (PCoA) plot of samples from all three anatomic sites. C. trachomatis-positive and -negative samples from vaginal (C) and endocervical (D) sites based on Bray-Curtis distance (beta diversity). The first and second principal coordinates are represented.

The heatmap in Fig. 3A shows the top 25 species (species limit set by Nephele pipeline) in the Bray-Curtis hierarchical clustering of endocervical, vaginal, and rectal samples with three distinct clusters. The first cluster consisted of C. trachomatis-negative vaginal and endocervical samples with a predominance of Prevotella amnii, G. vaginalis, A. vaginae, *L. iners*, and L. crispatus. The second cluster consisted of a combination of C. trachomatis-positive and three C. trachomatis-negative vaginal and endocervical samples. C. trachomatis-negative samples 57V and 57C had 12 and 6 C. trachomatis reads, respectively, detected in their metagenomic sequencing data that were confirmed by BLASTn, which may represent prior infection. Sample 121C showed only *G. vaginalis*. The only rectal sample in this cluster was 362R, which had a microbial composition similar to the microbiomes of 362C and 362V (see below). The other samples were dominated by BV pathogens, including Clostridiales genomosp., *BVAB3*, P. amnii, *G. vaginalis*, *A. vaginae*, *L. iners*, *Megasphaera genomosp. type_1*, Prevotella timonensis, and *Megasphaera* spp. The third cluster consisted of rectal C. trachomatis-negative and -positive samples, a single C. trachomatis-negative cervical sample (30C), and C. trachomatis-positive samples 107C and 107V. The predominant species varied across rectal samples but consisted mainly of Bifidobacterium adolescentis, Prevotella copri, Porphyromonas uenonis, Actinomyces turicensis, P. timonensis, Faecalibacterium prausnitzii, Eubacterium rectale, and Bacteroides fragilis (Fig. 3A).

Principal-component analysis (PCoA) based on Bray-Curtis distance (beta diversity) showed clustering patterns (Fig. 3B) that were identical to hierarchical clustering shown in Fig. 3A, with rectal samples primarily clustering separately. Comparing the beta diversity between the C. trachomatis-negative and -positive samples in the vagina (Fig. 3C) and endocervix (Fig. 3D), each formed mostly separate clusters. No clear clusters were present for the rectal samples.

### Endocervical and vaginal microbiota structures and CSTs.

Vaginal and endocervical microbial species resolved into CST-III-A, CST-III-B, CST-IV-A, CST-IV-B, and CST-IV-C0 (Table 2). CSTs were identified by the CST classifier VALENCIA that matches the microbiota profile to 13 reference centroids based on the similarity score (45). These scores were used to gauge confidence in the assignment and were helpful when there was no match or no close match to multiple centroids. In these cases, VALENCIA yielded a low confidence score (closer to zero), indicating ambiguity in the assigned CST (Table 2).

**TABLE 2:** Vaginal and endocervical community state types (CSTs)^*a*^ using the CST classifier VALENCIA and the relative abundance data from One Codex, VIRGO, Kraken, and MetaPhlAn databases

Sample^*b*^	Status of:						VIRGO		One Codex		Kraken		MetaPhlAn	
	C. trachomatis	BV	N. gonorrhoeae	*Candida*	HPV	M. genitalium	CST^*c*^	Score	CST^*c*^	Score	CST^*c*^	Score	CST^*c*^	Score
72C	+	−	−	−	−	−	IV-B	**0.10**	IV-B	0.08	IV-B	0.006	IV-B	0.009
72V	+	+	−	−	−	−	IV-B	**0.14**	IV-B	0.06	IV-B	0.01	IV-B	0.01
98C	+	−	−	−	−	−	IV-B	**0.10**	IV-B	0.10	IV-B	0.007	IV-B	0.05
98V	+	+	−	−	−	−	IV-B	**0.73**	IV-B	0.10	IV-B	0.11	IV-B	0.05
107C	+	−	−	−	−	***+***	IV-A	**0.36**	IV-A	0.02	IV-B	0.01	IV-A	0.01
107V	+	+	−	−	−	***+***	IV-A	**0.15**	IV-A	0.01	IV-B	0.04	IV-A	0.01
192C	+	−	−	−	−	***+***	IV-B	**0.14**	IV-B	0.04	IV-B	0.01	IV-B	0.02
192V	+	−	−	−	***+***	***+***	IV-B	**0.50**	IV-B	0.04	IV-B	0.14	IV-B	0.04
362C	+	−	−	−	−	***+***	IV-A	**0.38**	IV-B	0.05	IV-B	0.18	IV-B	0.02
362V	+	−	−	−	−	***+***	IV-A	**0.36**	IV-B	0.04	IV-B	0.11	III-A	0.06
30C	−	−	−	−	−	−	IV-B	0.01	IV-B	0.03	III-A	0.001	III-A	**0.05**
30V	−	+	−	−	−	−	IV-B	**0.22**	III-B	0.13	III-B	0.20	III-A	0.12
35C	−	−	−	−	***+***	−	***IV-C0***	0.003	IV-C3	0.03	IV-C3	0.007	IV-C3	**0.09**
35V	−	+	−	***+***	***+***	−	***IV-B***	**0.18**	III-B	0.10	III-A	0.02	III-A	0.12
57C	−	−	−	−	−	***+***	IV-B	**0.35**	IV-B	0.14	IV-B	0.05	IV-B	0.05
57V	−	+	−	−	−	−	IV-B	**0.67**	IV-B	0.11	IV-B	0.12	IV-B	0.06
121C	−	−	−	−	−	−	***IV-B***	0.04	IV-B	**0.06**	IV-B	0.002	IV-B	0.05
121V	−	−	−	***+***	−	−	***IV-B (III-A)***	**0.68**	III-A	0.14	III-A	0.13	III-A	0.11
319C	−	−	−	−	−	−	IV-C0 (III-B)	**0.20**	III-B	0.13	III-A	0.04	III-A	0.10
319V	−	−	−	+	−	−	III-B	**0.26**	III-B	0.17	III-A	0.07	III-A	0.11

a CSTs were assigned using the similarity of a microbiota profile to each of the 13 VALENCIA reference centroids based on the similarity score. VIRGO taxonomic data run in VALENCIA was used for defining CSTs;

b V, vagina; R, rectum; C, endocervix.

c Underlined CST, unique CST compared to the other three databases; bold italic CST, discordant between endocervix and vagina; CST in parentheses, assigned CST based on comparison with all VALENCIA reference centroids; bold score, highest value across all data sets; gray shading, variance from VIRGO CST; + sign refers to the sample being positive (for the respective organism or disease listed at the top of the column), and the − sign refers to the sample being positive (for the respective organism or disease listed at the top of the column); bold italic ***+***, detected by metagenomic shotgun sequencing (MSS).

Comparing the relative abundance data for the four databases run through VALENCIA, VIRGO (18) had the highest overall similarity scores compared to One Codex, Kraken, and MetaPhlAn, except for 30C, 35C, and 121C (Table 2). Although the score for 35C was the lowest for VIRGO, there was a relatively even community of *Prevotella* spp. with only 7% other spp., which was a better match with the reference CST-IV-C0 bacterial composition than CST-IV-C3, as determined by the other databases (Fig. S3). For 30C, both VIRGO and One Codex abundance data matched CST-IV-B, while 121C matched CST-IV-B for all databases; 121V was assigned CST-IV-B using the VIRGO database, although the microbiome was more similar to reference III-A (Fig. S4), which was the same for the other three databases. Similarly, 319C was assigned CST-IV-C0 but had a closer match to reference III-B (Fig. S5), as it did for the One Codex database.

**FIG 4:**
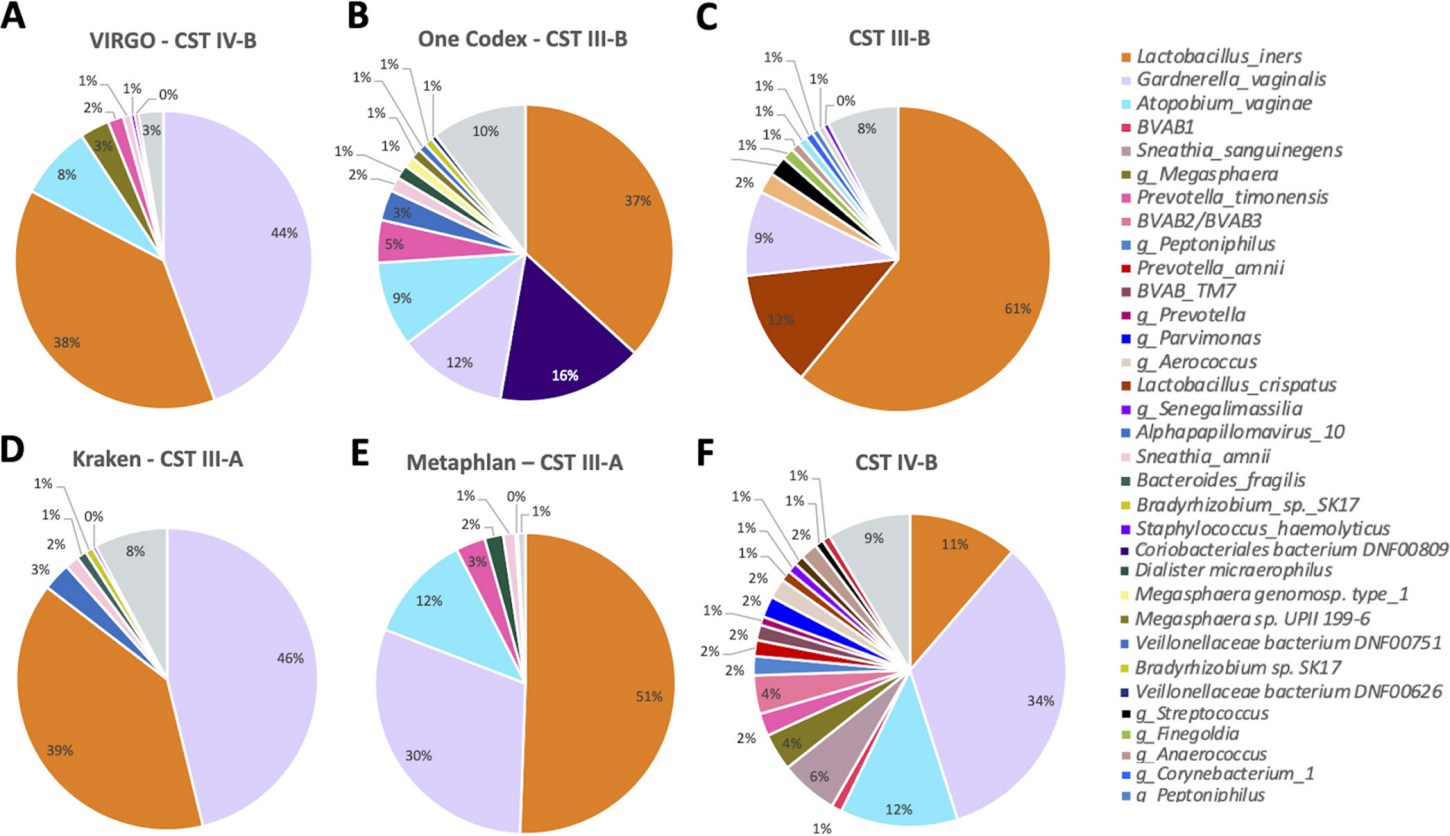
Pie chart representation of community state types (CSTs) for patient sample 35V identified by the CST classifier VALENCIA based on the taxonomic outputs generated by four different databases. Represented databases: VIRGO (A), One Codex (B), Kraken (D), and Metaphlan (E). The reference bacterial compositions for CST-III-B (C) and IV-B (F) are shown for comparison.

The microbiomes of C. trachomatis-negative women consisted of CST-III-A, CST-III-B, CST-IV-B, and CST-IV-C0, whereas C. trachomatis-positive women had CST-IV-A and CST-IV-B, although there was no significant association between C. trachomatis status and CST. C. trachomatis-negative patients 35 and 121 had discordant CSTs between the endocervix and vagina; 35V was assigned CST-IV-B with a best match to reference CST-IV-B (Fig. 4), whereas 35C was assigned, as described above, CST-IV-C0. 121V was assigned CST-III-A, while 121C was assigned CST-IV-B.

### Rectal enterotypes and existing gaps in the typing scheme.

**FIG 5:**
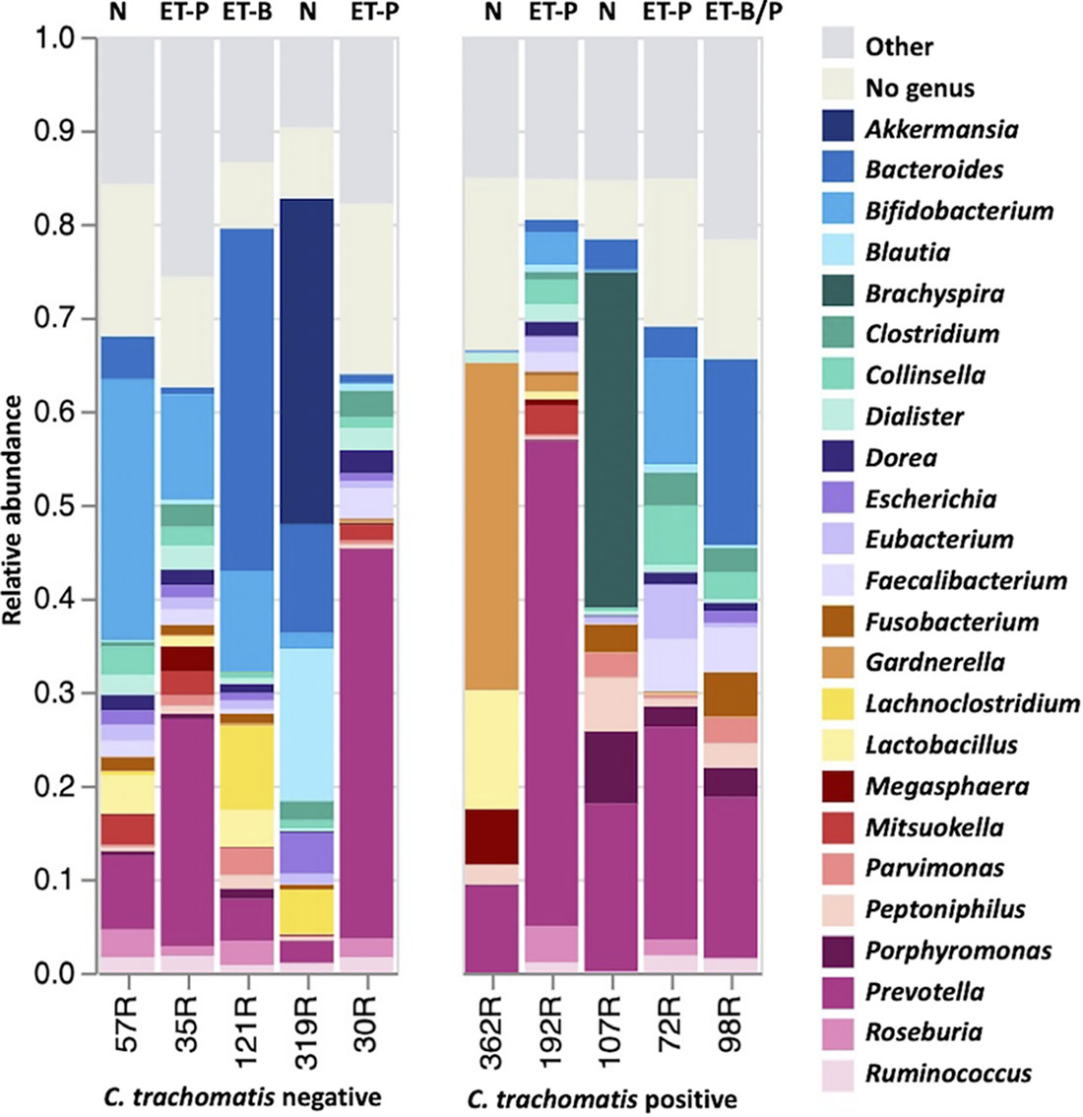
Enterotypes of rectal microbiota based on taxonomic relative abundances at the genus level for C. trachomatis-positive and -negative women. Only genera with relative abundance of >1% on average are reported. Remaining taxa are grouped as “Other.” Enterotypes are indicated at the top of each relative abundance bar: enterotype 1 (ET-B), *Bacteroides* as the best indicator; enterotype 2, *Prevotella* as the driver, a genus whose abundance is inversely correlated with *Bacteroides* (ET-P); enterotype 3, distinguished by an overrepresentation of *Firmicutes*, most prominently *Ruminococcus* (ET-F, not present); N, indicates that an enterotype could not be predicted based on current enterotype compositions.

The dominant genera of bacteria in the rectum, which probably reflects carryover from the gastrointestinal (GI) microbiome, have been defined by three enterotypes each dominated by *Bacteroides*, *Prevotella*, and *Ruminococcus*, respectively (46). These clusters may in fact be more appropriately characterized as a ratio of *Bacteroides* versus *Prevotella* abundance, with the *Ruminococcus* enterotype folded into the *Bacteroides* group (47, 48). Pathogenic rectal microbiota have been proposed to be characterized by reduced diversity with a shift away from commensal species. While the rectal microbiota is composed of primarily *Firmicutes*, *Bacteroidetes*, *Proteobacteria*, *Actinobacteria*, and *Fusobacteria*, our cohort occasionally also had microbiomes dominated by *Spirochaetes* and *Verrucomicrobia* (data not shown). Based on the enterotype method of classification (49), enterotype 2 (ET-P) was most common, which is dominated by genus *Prevotella.* Sample 98R was a combination of ET-P and enterotype 1 (ET-B), where *Bacteroides* predominates. However, four rectal microbiomes could not be placed into an enterotype and contained bacteria primarily from the genera *Gardnerella*, *Akkermansia*, *Bifidobacterium*, and *Brachyspira* (Fig. 5). There was no correlation between enterotype and C. trachomatis positivity, although the microbial composition of 362R was similar to the respective endocervical and vaginal microbiomes (Fig. 2).

### Predicted functional profiles of microbial communities in the endocervix, vagina, and rectum.

**FIG 6:**
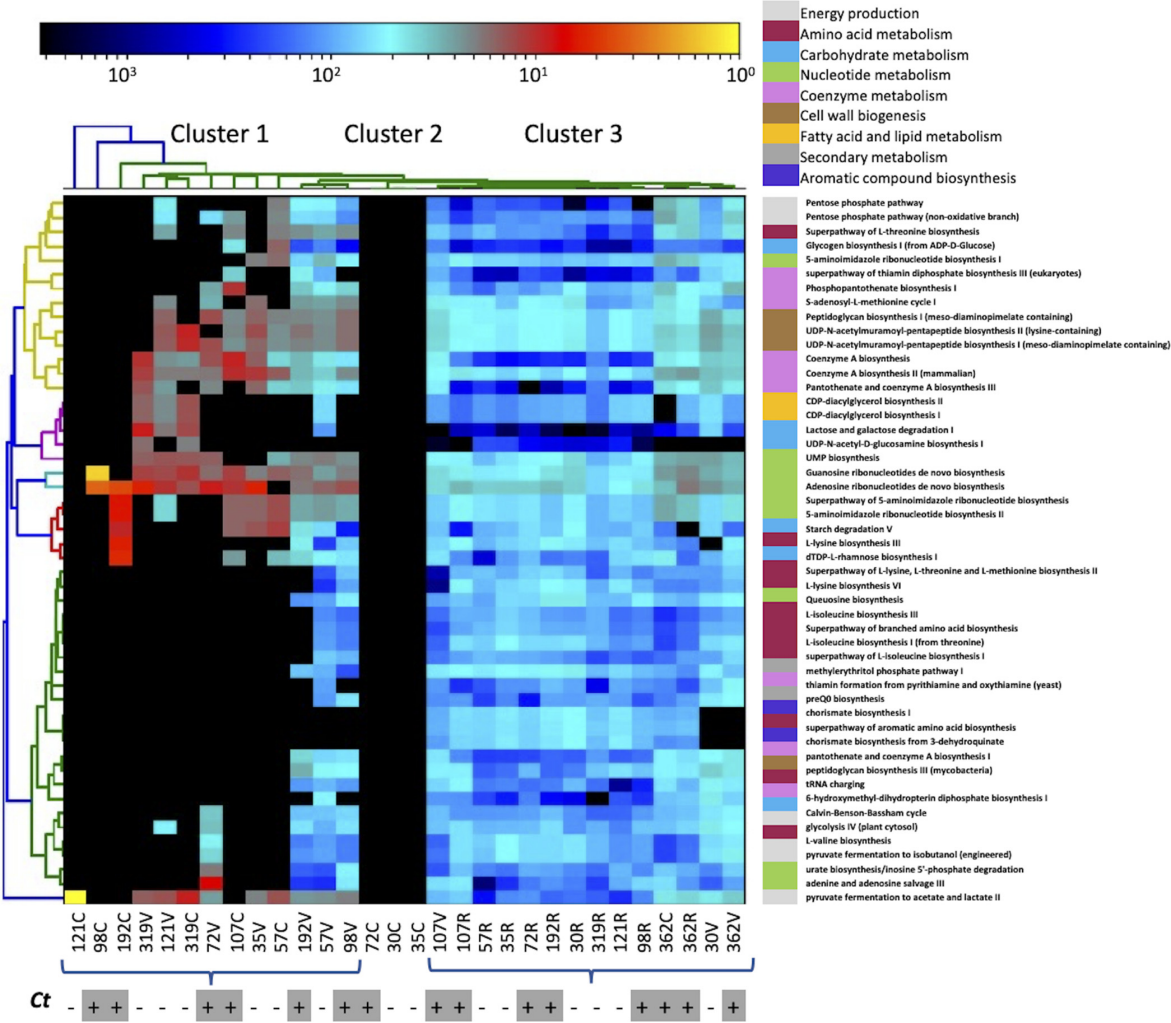
Spectral clustering of the top 50 predicted functional pathways in endocervical, vaginal, and rectal metagenomes. The clustering was based on the average abundance matrix of the predicted functional pathways with the greatest variance across the three anatomic sites. Rows represent the predicted functional categories of the bacterial communities, whereas the columns represent the sample identities. The color key of the average abundance is indicated above the heatmap. C. trachomatis infection status is indicated below the heatmap matrix. Key functional categories are indicated on the right above the functional pathway designations.

We characterized the microbial function at the whole-pathway level of the endocervical, vaginal, and rectal microbiomes using HUMAnN2 v3.0 (50). Substantial variability existed at the functional level across the three anatomic sites, with the samples segregating into three clusters based on pathway quantification (Fig. 6). The top 50 pathways (default pathway limit set by the Nephele pipeline) are visualized in the heatmap. Cluster 1 consisted of C. trachomatis-positive and -negative vaginal and cervical samples. Cluster 2 comprised only three endocervical samples. Cluster 3 consisted of rectal samples in addition to 107V, 362C, and 362V, where these three microbiomes were similar in composition to the rectal microbiomes. Samples 192V, 57V, and 98V in cluster 1 and samples 107V to 362V in cluster 3 had a higher abundance of pathways that were distinct from all other samples in cluster 1 and cluster 2.

No pathways were unique to the endocervical site. While the majority of the vaginal samples also had no unique pathways, the guanosine ribonucleotides *de novo* biosynthesis pathway of BVAB3 was unique to C. trachomatis-positive samples 72V, 107V, and 192V. Interestingly, 107V had many pathways driven by BV pathogens such as *Prevotella*, *BVAB3*, *Mobiluncus*, *Megasphaera*, *Peptoniphilus*, *Peptostreptococcus*, and *Anaerococcus* that were unique to this vaginal sample (Table S3). Several pathways that were identified as unique to the rectal site were also specific to rectal microbial species (Table S4). These pathways were mainly involved in chorismite, amino acid, coenzyme A (CoA), and nucleotide biosynthesis and carbohydrate metabolism. Sample 362R, which had a microbiome similar to vaginal microbiomes, had very few pathways unique to the rectum.

BV pathogens such as *P. amnii*, *P. timonensis*, *G. vaginalis*, *BVAB3*, Peptostreptococcus anaerobius, P. uenonis, and Porphyromonas asaccharolytica were the drivers of 57 pathways and showed a significant difference in abundance (*P* < 0.05) between C. trachomatis-positive and -negative endocervical samples (Table S5). The predicted pathways characterizing C. trachomatis-positive endocervical samples were involved in the biosynthesis of peptidoglycan, CoA, dTDP-l-rhamnose, l-lysine, UMP, CDP-diacylglycerol, UDP-N-acetylmuramoyl-pentapeptide, guanosine ribonucleotides, and NAD. In the vagina, 11 pathways were significantly greater in abundance in C. trachomatis-positive than in C. trachomatis-negative samples and were involved in CoA, amino acid, nucleotide and nucleoside, amines and polyamines, secondary metabolite, and enzyme factor biosynthesis (Table S6); 8 of the 11 pathways could not be attributed to microbiota at the species level. The remaining three pathways involved in CoA and nucleotide biosynthesis were driven by *P. amnii* and BVAB3.

A total of 38 pathways involved in carbohydrate, CoA, amino acid, cell structure, fatty acid and lipid, secondary metabolite, nucleotide and enzyme factor biosynthesis, and nucleoside, nucleotide, and carbohydrate degradation were significantly greater in abundance in the rectal C. trachomatis-positive samples than in the C. trachomatis-negative samples (Table S7). Nineteen of these pathways were found only in *P. lacrimalis*, *P. uenonis*, F. prausnitzii, Collinsella aerofaciens, *P. timonensis*, and Eubacterium biforme.

**FIG 7:**
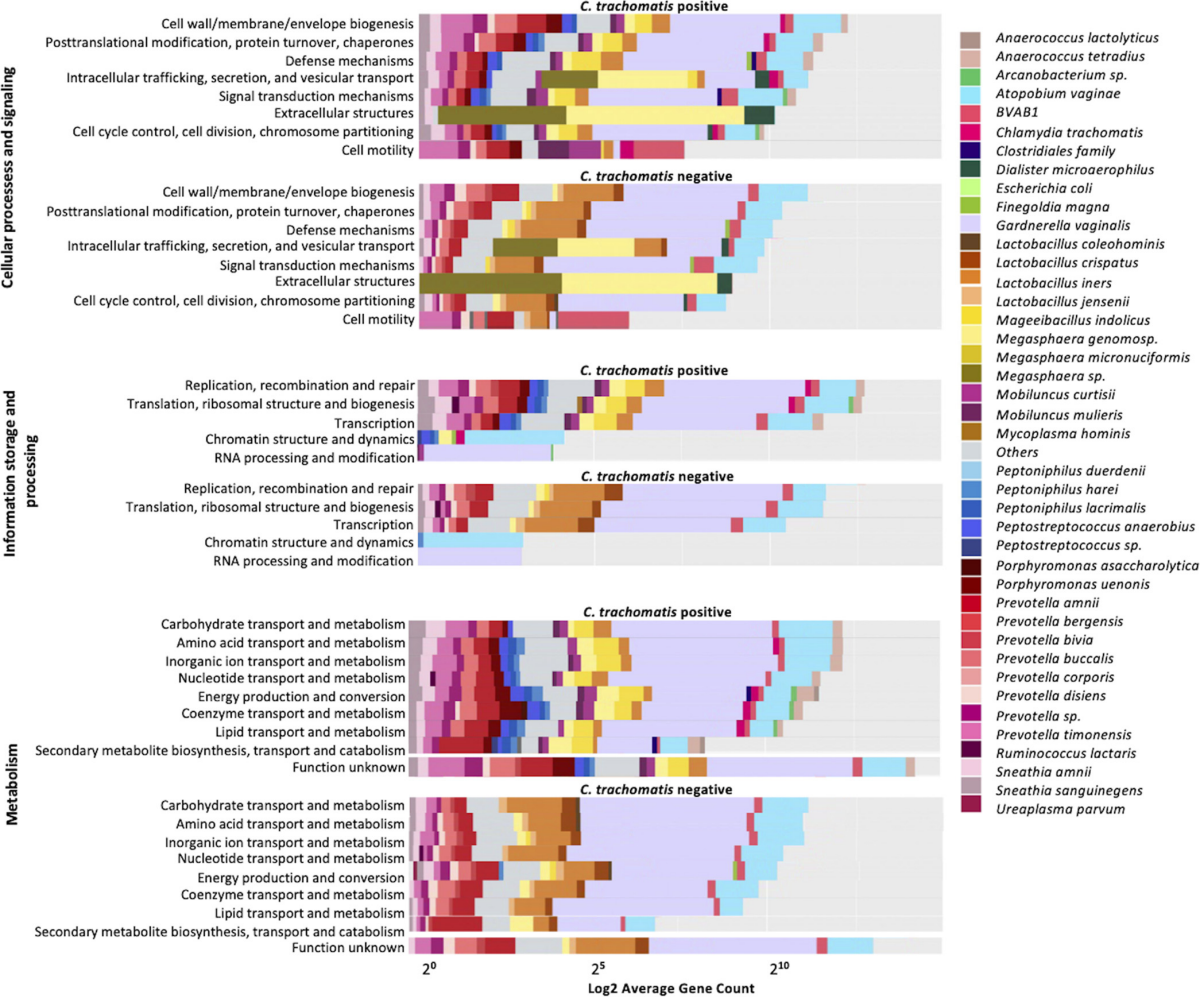
Functional profiling of the vaginal metagenomes for C. trachomatis-positive and -negative women. Functional categories were annotated using EggNOG v4.5 (see Materials and Methods). Functional profiles were stratified by species using the taxonomic profiling provided by VIRGO. *y* axis, functional categories; *x* axis, log 2 transformed gene counts for individual species.

Using VIRGO, we were able to characterize the abundance of genes within each species and categorize them into their functional categories for the vaginal and endocervical sites. While there was no significant difference in the average gene count across different functional categories between the vaginal C. trachomatis-positive and -negative samples, there was a clear difference in the species involved in the stratification of the functional categories (Fig. 7). Within the category of cellular processes and signaling, genes from the bacterial species *P. uenonis*, *P. anaerobius*, *P. lacrimalis*, Peptoniphilus harei, Mobiluncus mulieris, Mobiluncus curtisii, and A. tetradius were unique to C. trachomatis-positive compared to -negative samples. The pattern was very similar for the functional category information storage and processing and metabolism, where, in addition, *M. indolicus* was unique to C. trachomatis-positive compared to -negative samples. Similar to vaginal samples, there was no significant difference between the average gene count of functional categories between C. trachomatis-positive and -negative endocervical samples (Fig. S6). However, genes from *A. tetradius*, *Arcanobacter* spp., C. trachomatis, Dialister micraerophilus, *M. indolicus*, M. curtisii, Mobiluncus mulieris, *P. lacrimalis*, *P. anaerobius*, *Prevotella* spp., and *P. timonensis* were unique to C. trachomatis-positive samples for the functional category of cellular processes and signaling. In addition, *P. harei* was also unique to C. trachomatis-positive samples for the functional category of information storage and processing. *Peptostreptococcus* spp., Escherichia coli, *S. amnii*, Sneathia sanguinegens, and *R. lactaris* were unique to C. trachomatis-positive samples in addition to the species mentioned above for metabolism.

## DISCUSSION

The last decade has provided a plethora of data on the vaginal microbiome, defining both healthy and pathogenic microbial profiles in addition to associations with various STIs. Far fewer studies have similarly examined the endocervix. To the best of our knowledge, this is the first study to investigate the microbial composition, structure, and purported function of the endocervical, vaginal, and rectal microbiomes using MSS technology within and among women and also in association with C. trachomatis infection. Further, while several studies have highlighted racial and ethnic differences in the microbiome of healthy asymptomatic women as well as women with BV and STIs from ethnicities of European, Asian, African, and Hispanic descent (51–56), none have studied Pacific Islanders in these contexts.

The cohort of women in this study were of iTaukei ethnicity, which we had previously identified as at higher risk for STIs than Indo-Fijian and other Pacific Islanders (6). Women with C. trachomatis infection had a significantly higher mean load in the rectum than in the vagina and endocervix. It is possible that this reflects the higher percentage of human contamination for rectal than vaginal samples in that the former has not only a larger surface area of epithelial cells (approximate mean surface area of 193 to 212 cm^2^) (57) than the vagina (approximate mean surface area of 87 cm^2^) (58) but perhaps a greater number of cells that can be infected with C. trachomatis and then sloughed. This ties in with well-known trends in tissue-specific human contamination (59). However, one of our study subjects had a higher vaginal load that may indicate intragenitorectal transmission given the similarity in composition across all three microbiomes. The latter case is consistent with a recent study in the Netherlands that found a higher mean vaginal load than concurrent anorectal infection (60). A much larger sample size will be needed to discern the clinical significance of differential loads among these anatomic sites.

The vaginal Xpert CT/NG and wet prep tests were able to detect N. gonorrhoeae, *Trichomonas vaginalis*, and *Candida* in the cervicovaginal environment, with one additional candidiasis case detected by MSS. MSS identified one other subject with rectal candidiasis for which there is no standard test. Interestingly, subject 57, who tested negative for C. trachomatis, had six C. trachomatis reads in the endocervix, 12 in the vagina, and 2 in the rectum, suggesting a prior infection that may have recently cleared but with the presence of residual DNA. Indeed, C. trachomatis DNA can be detected by commercial diagnostic tests for 2 to 4 weeks following successful treatment (61). MSS was also instrumental in detecting 2 subjects with HPV, 5 with rectal N. gonorrhoeae, and all 10 with M. genitalium infections (3 of whom had M. genitalium in other anatomic sites) where pap smear, rectal STI, and M. genitalium tests, respectively, were not available. The N. gonorrhoeae findings might be expected in that recent studies have found rectal infections in women to be as high as 13.4% irrespective of urogenital infections based on nucleic acid amplification tests (NAATs) (28, 62). However, finding M. genitalium in all rectal microbiomes was somewhat surprising, although rectal M. genitalium has frequently been reported as asymptomatic and more common than urogenital infections, at least among men who have sex with men (MSM) (63). There are extremely limited data on women, but our findings suggest that MSS may be more sensitive than NAATs in detecting rectal M. genitalium. MSS holds promise as a scalable global microbial “diagnostic” for STIs for near-future use.

The species-level alpha diversity among vaginal samples from our cohort was similar to that of other studies (64). However, we found that the diversity was higher in the endocervix than in the vagina for both C. trachomatis-positive (mean 3.8 versus 3.1) and -negative (mean 2.7 versus 1.8) women, although not statistically significantly. These results were supported by a study of high-risk adolescents in South Africa where the endocervix was also found to have a higher diversity than the vagina based on 16S rRNA sequencing (11).

While previous studies have largely asserted that endocervical and vaginal microbiomes have similar microbial populations (65, 66), this was not the case in our study. In general, for C. trachomatis-positive women, the relative abundance of *R. lactaris*, *P. lacrimalis*, *A. tetradius*, and P. oris was higher in the vagina than in the endocervix, whereas *A. tetradius* was higher in abundance in the endocervix. These data are consistent with a 16S rRNA sequencing-based study where young women were found to have significantly dissimilar microbiomes between the endocervix and vagina. For example, the abundant species in the former were Achromobacter spanius, Gordonia terrae, and Enterococcus faecium (11). Dissimilar microbiomes were also identified in a study of HPV infection among women in China (67). Another recent publication that did not address STIs also found differences in 16S rRNA microbial profiles between these two anatomic sites (68). Additional research is required to determine the full breadth of endocervical and vaginal microbial discordance among C. trachomatis-positive and -negative subjects and the statistical associations of microbial species with each.

The endocervical and vaginal microbiota were dominated by *L. iners* and L. crispatus in C. trachomatis-negative women. In the vagina, the relative abundance of *L. iners* and L. crispatus in C. trachomatis-negative women was significantly higher than that in C. trachomatis-positive women, whereas, in the endocervix, the relative abundance of *L. iners*, L. crispatus, and L. jensenii was significantly higher in C. trachomatis-negative than -positive women. *L. iners*-dominated sites also harbored strict anaerobes, such as *A. vaginae*, *G. vaginalis*, and *Prevotella* spp., defined as “Intermediate BV microbiota,” which is considered a transient state between the mixed anaerobic and *Lactobacillus*-dominant microbiota (69). *L. iners* is biologically unique from other species of the genus since it is able to survive among different community members and also has a greater potential for disease pathogenesis (69–71). While these findings are somewhat surprising for C. trachomatis-negative women, they likely reflect the fact that many Fijian women have BV, similar to other minority, ethnic, and racial populations, such as Black and Hispanic women in the United States (44, 55, 56). Indeed, three of the five C. trachomatis-negative women in our study had clinical BV.

C. trachomatis-positive women had primarily polymicrobial vaginal and endocervical microbiomes comprised of *G. vaginalis*, *Prevotella* spp., *M. indolicus*, *Sneathia* spp., and *Veillonellaceae* spp., which are pathogens previously reported in several ethnicities with BV (72). In the vagina, the relative abundance of *M. indolicus*, *R. lactaris*, *P. lacrimalis*, *A. tetradius*, and *P. oris* in C. trachomatis-positive women was significantly higher than that in C. trachomatis-negative women, while, in the endocervix, the relative abundance of *M. indolicus* and *A. tetradius* was significantly higher than that in C. trachomatis-negative women. Although these species have previously been reported in association with BV, the combination among the C. trachomatis-positive women in this study appears to be unique to the Pacific Islanders.

Vaginal and endocervical samples were classified into CSTs using four databases and the VALENCIA classifier. The VIRGO database had the highest similarity scores compared to all other databases, which most likely reflects the fact that it was developed to include the current global diversity of vaginal microbial populations from North America, Africa, and Asia (18). However, there were five exceptions in our data set where VIRGO either had a lower score but the best CST match based on examination of the microbial composition or had the highest score but more closely matched another CST than the original assignment. Four of the five exceptions were endocervical samples (Table 2). These data suggest that the microbial populations of Pacific Islanders do not entirely fit the current VALENCIA classification system. Additional modifications to the CSTs will be required for endocervical and vaginal microbiomes of Pacific Islanders and other ethnic/racial groups as more globally diverse communities are examined in order to broaden our understanding of microbial profiles and structures across populations.

The rectal microbial profiles had an alpha diversity (mean of 5.2 for C. trachomatis-negative and 5.1 for C. trachomatis-positive women) much higher than those of the vagina and endocervix microbial profiles. Interestingly, C. trachomatis-positive subject 362 had a rectal microbial composition almost identical to the microbial profiles of the subject’s endocervix and vagina as evidenced by clustering of 362R (Fig. 3B, yellow data point) with 362V and 362C (Fig. 3B, blue and pink data points) in the PCoA plot based on Bray-Curtis distance. There was similarity of composition across the anatomic sites, including *Veillonellaceae* spp., *S. amnii*, *Prevotella* spp., *M. genomosp. type_1*, *M. indolicus*, *L. iners*, *G. vaginalis*, Clostridioides difficile, and *A. vaginae*. These data suggest a high degree of microbial sharing between anatomic sites or even transmission from the endocervix/vagina to the rectum. A recent study in Italy also found similarities between the vaginal and rectal taxa suggestive of microbial sharing along with what they described as dysbiosis in both sites (22). Overall, however, principal-component analysis showed that the microbial composition of the rectal samples clustered separately from the endocervical and vaginal samples with no distinct clustering based on C. trachomatis status.

The healthy human gastrointestinal (GI) microbiome has been found to be primarily composed of *Firmicutes*, *Bacteroidetes*, *Proteobacteria*, *Actinobacteria*, and *Fusobacteria* (46), which was consistent for the rectal microbiome of Pacific Islanders in our study. However, in our study, the rectal microbiome was occasionally also dominated by *Spirochaetes* and *Verrucomicrobia*. While a previous 16S rRNA study showed that *Firmicutes*, *Bacteroidetes*, *Proteobacteria*, and *Fusobacteria* were the top four dominant phyla in healthy and C. trachomatis-positive men who have sex with men (MSM) (35), our study showed that *Firmicutes*, *Bacteroidetes*, *Actinobacteria*, and *Fusobacteria* were the top four in C. trachomatis-negative women and *Firmicutes*, *Bacteroidetes*, *Actinobacteria*, and *Proteobacteria* were the top four in C. trachomatis-positive women. Six of 10 women (2/6 C. trachomatis-negative and 4/6 *C.-trachomatis*-positive) in our study also had a higher *Prevotella/Bacteroides* ratio, characteristic of a high-fiber and -carbohydrate diet of nonwesternized populations, which was similar to previous 16S and reverse transcription quantitative PCR (RT-qPCR) studies on the fecal microbiota of adults in Papua New Guinea (73, 74).

Due to a lack of convention for classifying rectal microbiota, we used the enterotype classification system, which is based on GI microbiota (49, 75), as a proxy. We also used three of the largest available metagenomic data sets, including the Human Microbiome Project (HMP), MetaHIT, and a Chinese type II diabetes study. We found that Pacific Islanders primarily had ET-P dominated by the genus *Prevotella* but also ET-B dominated by *Bacteroides* and one subject with a mixed ET-B/P enterotype. However, the microbial profile of two C. trachomatis-positive and two C. trachomatis-negative women was unique. These rectal microbiomes were dominated by *Gardnerella* and *Brachyspira* for C. trachomatis-positive women and *Akkermansia*, *Bifidobacterium*, and *Blautia* for C. trachomatis-negative women. The data highlight the limitations to the enterotype classification system because it does not entirely represent the rectal microbiome, and the resulting stratification only partially reflects the more complex structure in our population. Consequently, there is a need for improved modeling and inclusion of globally diverse communities to create typing schemes that are more inclusive. This would allow for a systematic comparison of Pacific Islander microbiomes to those of westernized or other ethnic populations that currently is not possible.

A higher involvement of CoA, aromatic amino acids, nucleotide and nucleoside, and enzyme factor biosynthesis pathways was predicted in the microbiome of C. trachomatis-positive patients, and many of these pathways were driven by pathogens associated with BV. Although functional studies to understand the dynamics of these anatomic ecological niches are warranted, we can speculate that these changes in metabolic activities are associated with pathogenic microbes and that the presence of these pathways in other bacteria result in support of chlamydial intracellular survival, growth, and development (76). For instance, C. trachomatis is dependent on the aromatic amino acid tryptophan. Most, but not all, genital and rectal C. trachomatis strains are tryptophan auxotrophs with tryptophan biosynthesis capabilities using substrates such as indole (77, 78). However, the organism can also scavenge tryptophan produced by other bacteria in the microbiome (79). Fundamentally, C. trachomatis displays reduced metabolic capabilities compared to its free-living *Chlamydia*-like counterparts and is therefore dependent on a wide range of host-derived metabolic precursors, purines and pyrimidines, and various cofactors (76). In support of our data, a recent 16S rRNA sequencing-based study of the vaginal microbiome predicted higher chorismate, a precursor of indole, and biosynthesis of aromatic amino acids in C. trachomatis-positive subjects than in C. trachomatis-negative subjects (22).

We found a higher abundance of CoA biosynthesis that was driven primarily by anaerobic BV pathogens in the C. trachomatis-infected endocervical, vaginal, and rectal microbiomes. CoA plays an important role in the citric acid cycle to generate energy and in mixed acid fermentation. Indeed, fermentation produces short-chain fatty acids that are one of the main drivers of an imbalance in the microbiota that can lead to disease pathogenesis (80). While a previous study also reported similar trends of increased fermentation in the C. trachomatis-positive vaginal microbiome (22), it was not clear if the pathways identified were driven by BV-associated pathogens.

The predicted pathways involving peptidoglycan biosynthesis and pyrimidine and purine metabolism by BV pathogens such as *G. vaginalis* were in higher abundance in the C. trachomatis-infected endocervical and rectal microbiomes than in the vaginal microbiome. Importantly, the C. trachomatis-infected endocervix had a number of metabolic pathways for CoA, amino acid, nucleoside and nucleotide, and enzyme biosynthesis driven by pathogens such as *Clostridiales*, *P. uenonis*, *P. amnii*, *P. timonensis*, and *Pophyromonas asaccharolytica* that were 5-fold higher than in the vagina. A recent study reported similar findings of a relatively more active and complex metabolism occurring in the HPV-infected endocervix compared to the vagina (67). Furthermore, folate, carbohydrate, cell structure, fatty acid and lipid, and secondary metabolite biosynthesis and amino acid and carbohydrate degradation pathways driven by *G. vaginalis*, *P. lacrimalis*, *P. uenonis*, and *Peptostreptococcus* were unique to the C. trachomatis-infected endocervix compared to the vagina. These functions are likely important for the survival of the aforementioned pathogens but also may indirectly support C. trachomatis infection. These novel data suggest that microbial functions in the endocervix are more diverse and complex than those in the vagina during C. trachomatis infection.

We acknowledge the limitation of our small sample size comparing the C. trachomatis-positive and -negative groups across the anatomic sites. The findings nevertheless provide a detailed pipeline and, more importantly, greater insight into the microbiomes of Pacific Islanders. Further research, including larger prospective studies, is required to unravel the role of microbiomes in acquisition and prevention of C. trachomatis infection and to better classify the genitorectal microbiomes in this population.

## MATERIALS AND METHODS

### Study design and sample characteristics.

Women attending the Fijian Ministry of Health and Medical Services (MoHMS) health centers and outreach clinics in the Central Division, Viti Levu, Fiji were consequently enrolled in a nonprobability parent study after informed consent as described previously (6). Women were excluded from the study if they were HIV positive, had untreated syphilis, had a diagnosis of cancer, or had been treated with antibiotics within the prior month.

Endocervical, vaginal, and rectal swab samples were collected similarly by trained clinicians and placed in SWAB/A-50 buffer (Cepheid, Sunnyvale, CA) and 1 mL of M4 medium (Remel, Lenexa, KS). Vaginal samples were collected as described previously (6). To ensure no contamination of the endocervix with vaginal material, after inserting the speculum and visualizing the cervix, the ectocervix was cleaned with a large swab to remove any material at this site, including the cervical os. A FLOQ swab (Copan) was then carefully inserted through the os into the endocervix without touching the speculum or walls of the vagina. Rectal samples were collected last using a FLOQ swab. All samples were stored at 4°C for no more than 4 h before transport to the lab at the same temperature. Vaginal samples in Cepheid buffer were kept at 4°C until processed same day; remnant Cepheid buffer samples were stored at −80°C. All samples for MSS were immediately placed at −80°C until processed.

Vaginal samples were tested for C. trachomatis and N. gonorrhoeae using the nucleic acid amplification test (NAAT) Xpert CT/NG test (Cepheid). Endocervical, vaginal, and rectal samples were tested by an in-house qPCR to detect the C. trachomatis single-copy *omp*A gene and human single-copy beta-actin gene and to quantitate each as we described previously (37). C. trachomatis genome copy number was then divided by the beta-actin genome copy number to arrive at the number of C. trachomatis genomes per cell.

All swab samples (stored at −80°C) along with clinical data and test results for C. trachomatis, beta-actin, Trichomonas vaginalis, *Candida*, and BV were provided by the parent study deidentified using a unique ID number with no trace to patient identifiers. We randomly selected (using a table of random numbers) five women with confirmed C. trachomatis infection in all three anatomic sites and age-matched them to five women without C. trachomatis infection in any site (Table 1).

### Nucleic acid extraction, library construction, and MSS.

Genomic DNA (gDNA) was extracted from endocervical, vaginal, and rectal swab samples from the 10 women (Fig. 1). Briefly, cells were lysed using 100 μL of lysozyme (10 mg/mL; Sigma-Aldrich), 12 μL of mutanolysin (25,000 U/mL; Sigma-Aldrich), and 6 μL of lysostaphin (4,000 U/mL in sodium acetate; Sigma-Aldrich) and incubated for 1 h at 37°C as we described previously (37). DNA was isolated from the resulting crude lysate using the QIAamp DNA minikit (Qiagen) as per the manufacturer’s instructions with elution in 100 μL of AE buffer. DNA concentration was measured using the Qubit double-stranded DNA (dsDNA) broad-range (BR) assay kit (Invitrogen). MSS libraries were constructed using Illumina Nextera XT kits and sequenced using 150-nucleotide paired-end reads on an Illumina HiSeq 2500 platform.

### Identification of endocervical, vaginal, and rectal microbiota and metabolic function.

Raw MSS sequence data were processed using FastQC v1.0.0 (81) to identify any problem areas in the data. TrimGalore v0.6.5 (82) was used to excise Illumina adapters, trim reads at an average quality score threshold of Q15, and remove reads containing ambiguous bases or sequences that were too short. All human reads were removed using Kneaddata v. 0.11.0 (83) (Fig. 1). Bacterial and viral reads were confirmed by BLASTn (query coverage of >95% and sequence identity of >95%) to the species level using custom python scripts back in blast.sh and BLAST_to_Excel.py (https://github.com/ddeanlab/Fiji-10-Patients-MSS). The number of confirmed reads for a pathogen was divided by the total number of reads for a sample, excluding human reads, and then multiplied by 1 million to arrive at a value for reads per million (RPM) for that pathogen as described by Babiker et al. (38).

The NIH-supported Nephele metagenome pipeline (84) was used on the raw reads, including the bioBakery package MetaPhlAn v3.0 (84) for species identification, StrainPhlan v3.0 (50) for identifying single nucleotide polymorphism (SNP) variation within species, and HUManN2 v3.0 (50) for identifying functional modules (Fig. 1). Functional pathways unique to each anatomic site were identified using a custom python script path_abundance.py.

One Codex (85), Kraken v2.0 (86), MetaPhlAn 3.0 (84), and VIRGO (18) were used to determine the taxonomic composition and presence of bacteria, fungi, protozoa, and viruses in each sample.

### Determination of CSTs for endocervical and vaginal samples.

For classification of vaginal and endocervical microbiota into CSTs, a nearest-centroid-based algorithm, VALENCIA (45), was applied to taxonomic files generated independently by One Codex, Kraken v2.0, MetaPhlAn v3.0, and VIRGO. CSTs were assigned based on the similarity of a microbiota profile to each of the 15 reference centroids calculated using Yue and Clayton’s *θ* (87). This yielded an array of 15 similarity scores, ranging from 0.0 (no shared taxa) to 1.0 (all taxa shared at the same relative abundance) for each sample. The reference centroid to which the sample bears the highest similarity score provided an assignment to a sub-CST.

### Enterotyping of rectal microbiota.

Phylum- and genus-level relative abundance of rectal samples was calculated and plotted in One Codex. Rectal samples were classified based on three enterotypes by the following criteria (49): (i) enterotype 1 (ET-B), with *Bacteroides* as its best indicator, (ii) enterotype 2 (ET-P), driven by *Prevotella*, a genus whose abundance is inversely correlated with *Bacteroides*, and (iii) enterotype 3 (ET-F), distinguished by an overrepresentation of *Firmicutes*, most prominently *Ruminococcus* (49).

### Statistical analysis and visualization.

Relative abundance of the top 28 species predicted by One Codex in the combined C. trachomatis-positive and C. trachomatis-negative samples for each site was compared using the one-sample Wilcoxon test and visualized using R software (88) and the ggplot2 package (v3.3.3) (89). The Shannon and Simpson index alpha-diversity was calculated in One Codex, and the Wilcoxon rank sum test was used to compare C. trachomatis-positive and -negative samples for each anatomic site. To visualize distances between samples, we used PCoA plots based on taxa at species rank and PCoA plots based on the beta-diversity metric Bray-Curtis dissimilarity, Manhattan distance, Jaccard distance, weighted UniFrac, and unweighted UniFrac. Bray-Curtis dissimilarity plot was chosen and plotted for samples from all three sites in One Codex. Heatmaps of the average species abundance (Spearman and Bray-Curtis) were plotted in Nephele. Wilcoxon rank sum test was used on pathway abundance files generated from HUMAnN2 v3.0 to compare C. trachomatis-positive and -negative samples for each anatomical site using the custom script Pathway_statistics.RMD. The average abundances of the top 50 functional pathways were plotted using Nephele.

### Data availability.

The human read-removed FASTQ files were submitted to the SRA under the BioProject accession number PRJNA826539.
