# Supplementary material for: Metagenomic Shotgun Sequencing of Endocervical, Vaginal, and Rectal Samples among Fijian Women with and without Chlamydia trachomatis Reveals Disparate Microbial Populations and Function across Anatomic Sites: a Pilot Study
Source: Microbiol Spectr. 2022 May 17;10(3):e00105-22. doi: 10.1128/spectrum.00105-22 (PMC9241848; doi:10.1128/spectrum.00105-22)
Supplement: SUPPLEMENTAL FILE 1 — Supplemental material. Download spectrum.00105-22-s0001.pdf, PDF file, 4.2 MB [file spectrum.00105-22-s0001.pdf]

Supplementary Material

Metagenomic shotgun sequencing of endocervical, vaginal and rectal samples among Fijian women with and without *Chlamydia trachomatis* reveals disparate microbial populations and function across anatomic sites: A pilot study

Sankhya Bommana, Gracie Richards, Mike Kama, Reshma Kodimerla, Kenan Jijakli, Timothy D. Read, Deborah Dean

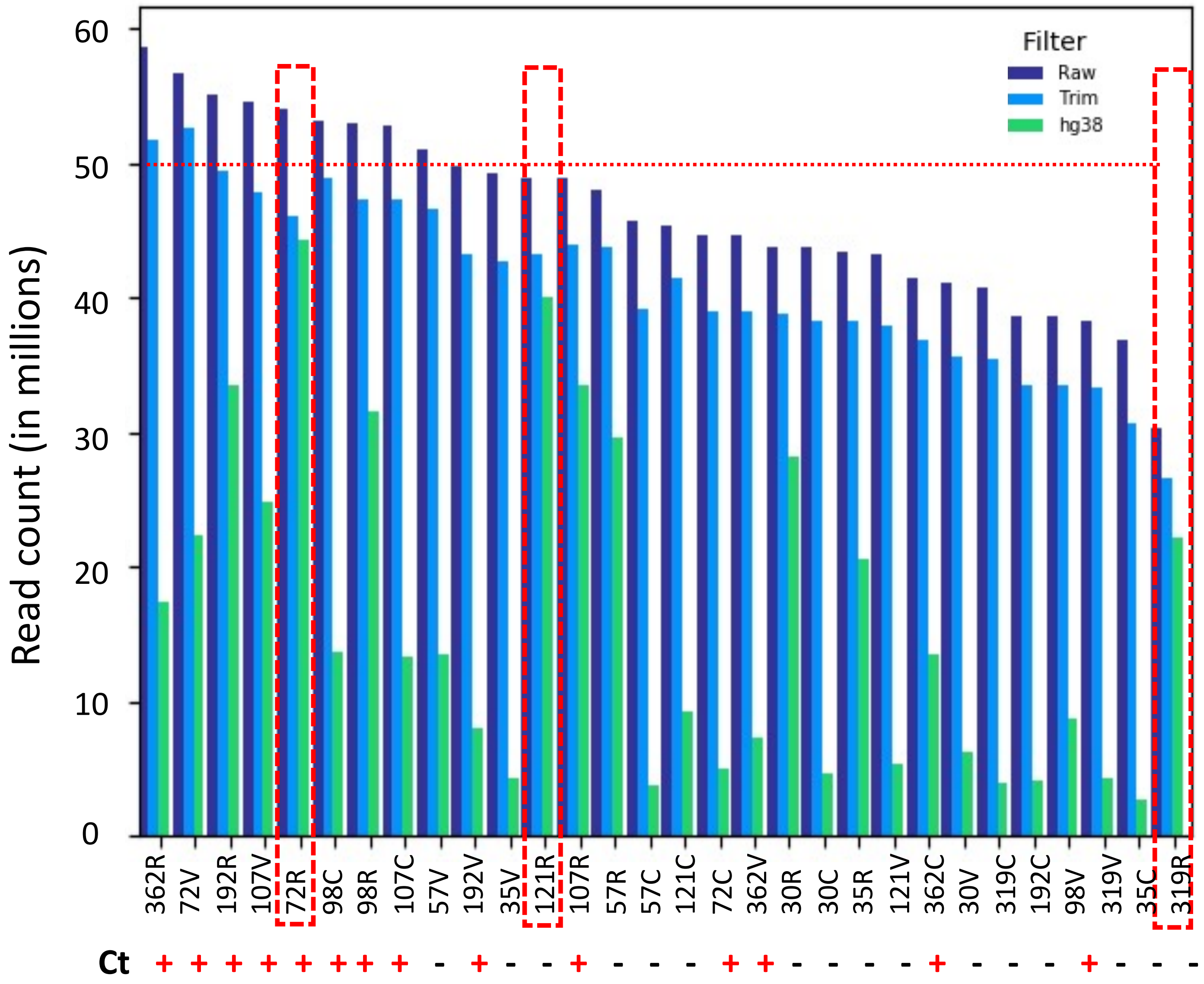

Supplementary Figure 1. Bar graph depicting sample characteristics of the DNA paired end reads from metagenomic shotgun sequencing on an Illumina HiSeq 2500 platform. Dark blue bars, raw paired end reads; Blue bars, trimmed reads; Green bars, contaminating human reads. An increase in contaminating human DNA was noted in three rectal samples, 72R, 121R and 319R, denoted by hatched red rectangles. Samples positive (+) or negative (-) for *C. trachomatis* (*Ct*) are noted below the graph.

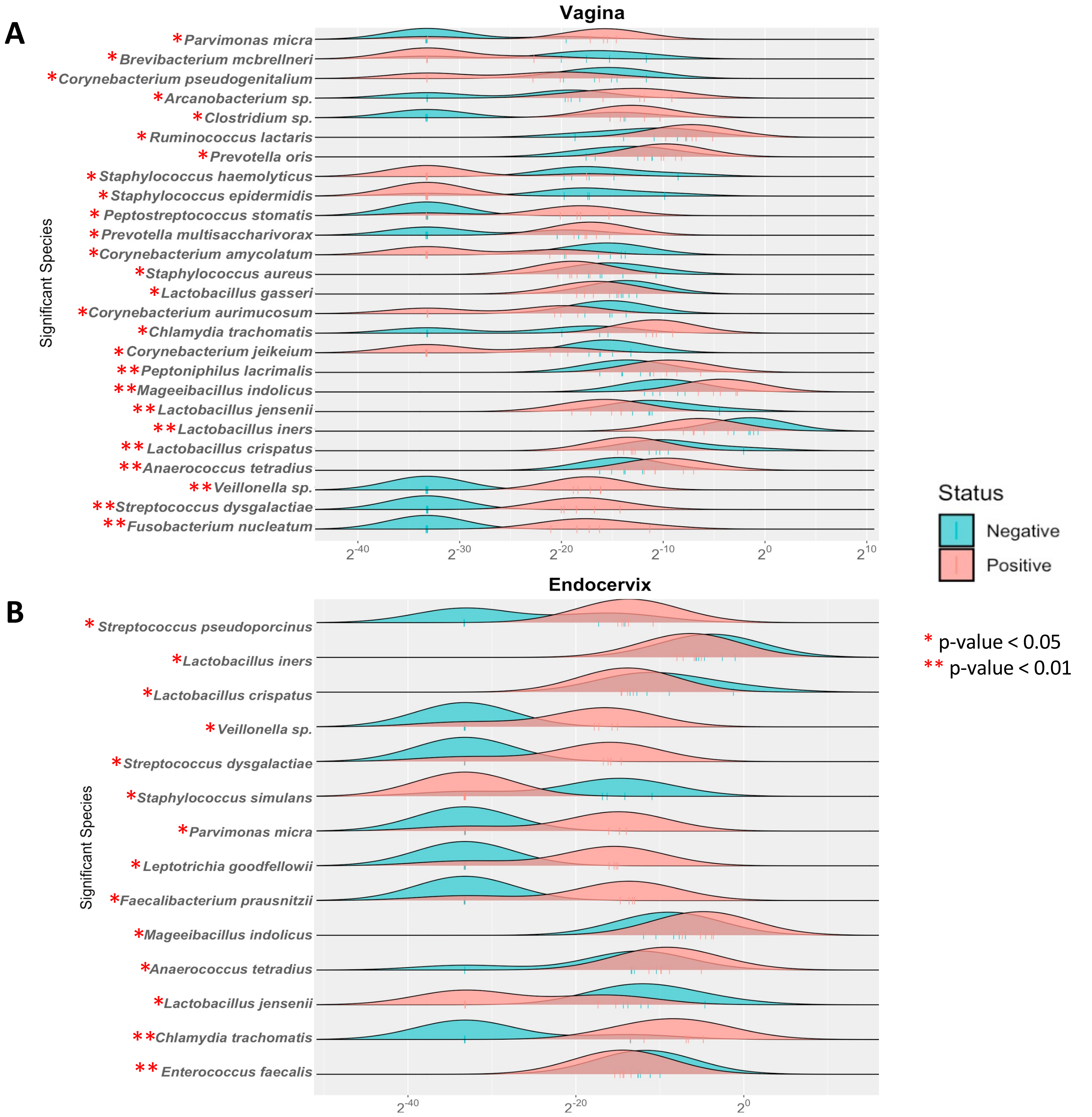

Supplementary Figure 2. Relative abundance of all significantly different species between *C. trachomatis* (*Ct*) positive and negative women for vaginal (A) and endocervical (B) microbiomes. The most unique abundant gene content in VIRGO is plotted for each site (see Materials and Methods). Density curves for each species are shown for the distribution of the relative abundance of reads for *Ct* positive versus negative subjects. The vertical bars represent the individual data points. Significant differences in relative abundance of species calculated using the two-sample Wilcox test are shown. X-axis represents the logarithm to the base 2 of the ratio of the unique gene content of a species divided by the gene content of the entire community. *, p-value <0.05; ** p-value <0.01.

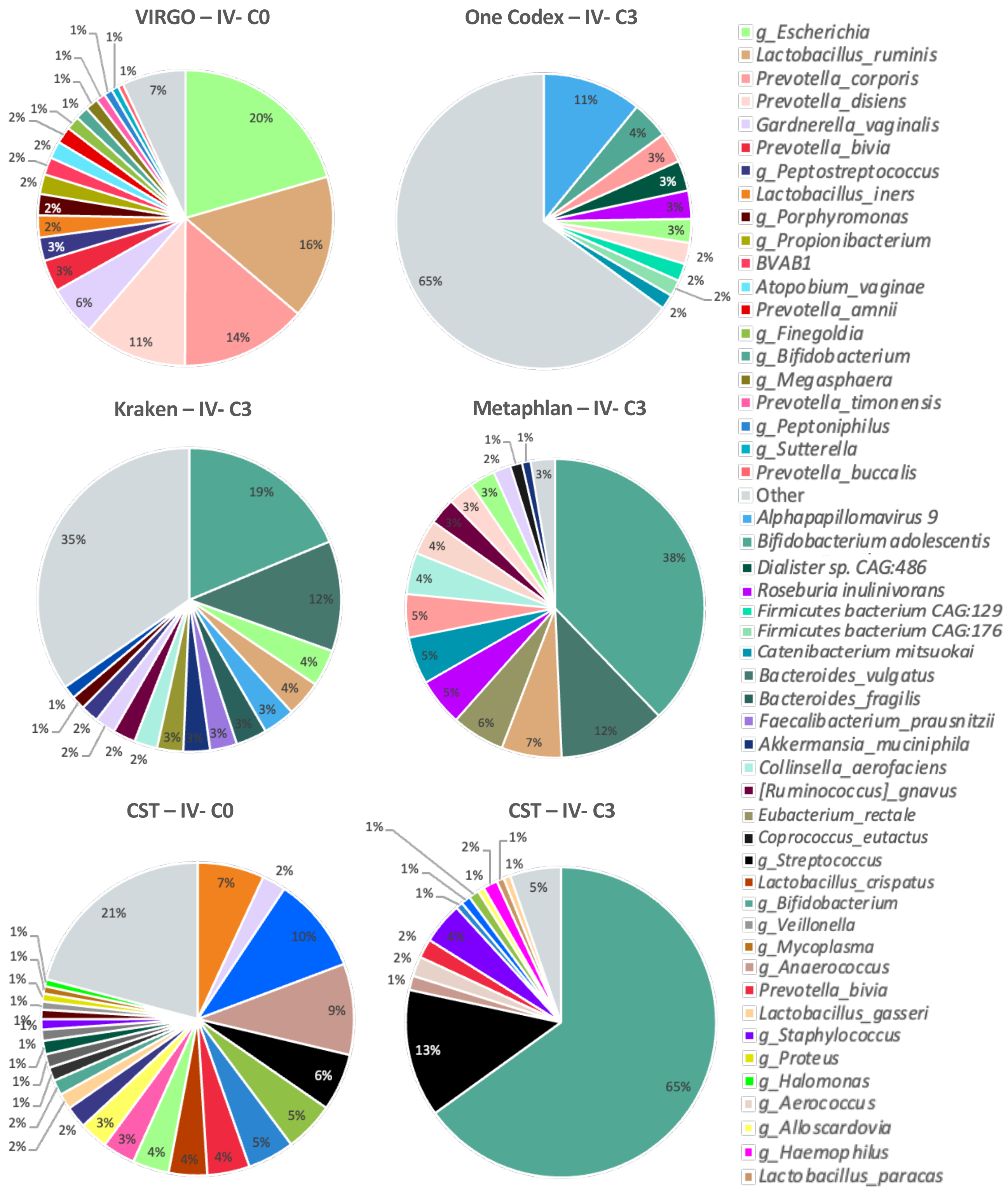

Supplementary Figure 3. Pie chart representation of Community State Types (CSTs) for patient sample 35C identified by the CST classifier VALENCIA based on the taxonomic outputs generated by four different databases: (A) VIRGO; (B) One Codex; (C) Kraken; and (D) Metaphlan. The reference bacterial compositions for CST IV-C0 and IV-C3 are shown for comparison.

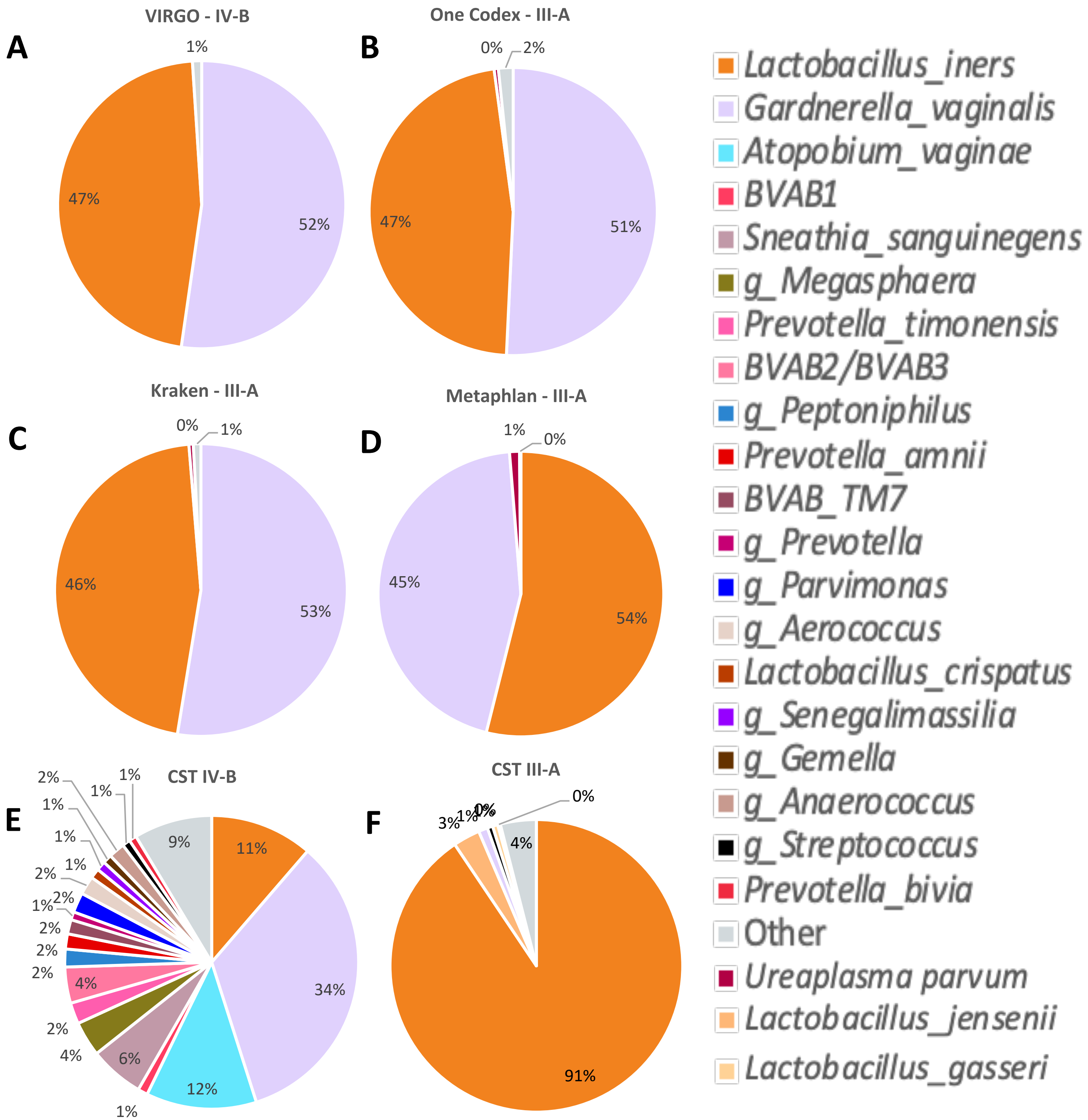

Supplementary Figure 4. Pie chart representation of Community State Types (CSTs) for patient sample 121V identified by the CST classifier VALENCIA based on the taxonomic outputs generated by four different databases. Represented databases: VIRGO (A), One Codex (B), Kraken (C) and Metaphlan (D). The reference bacterial compositions for CST IV-B and III-A are shown for comparison.

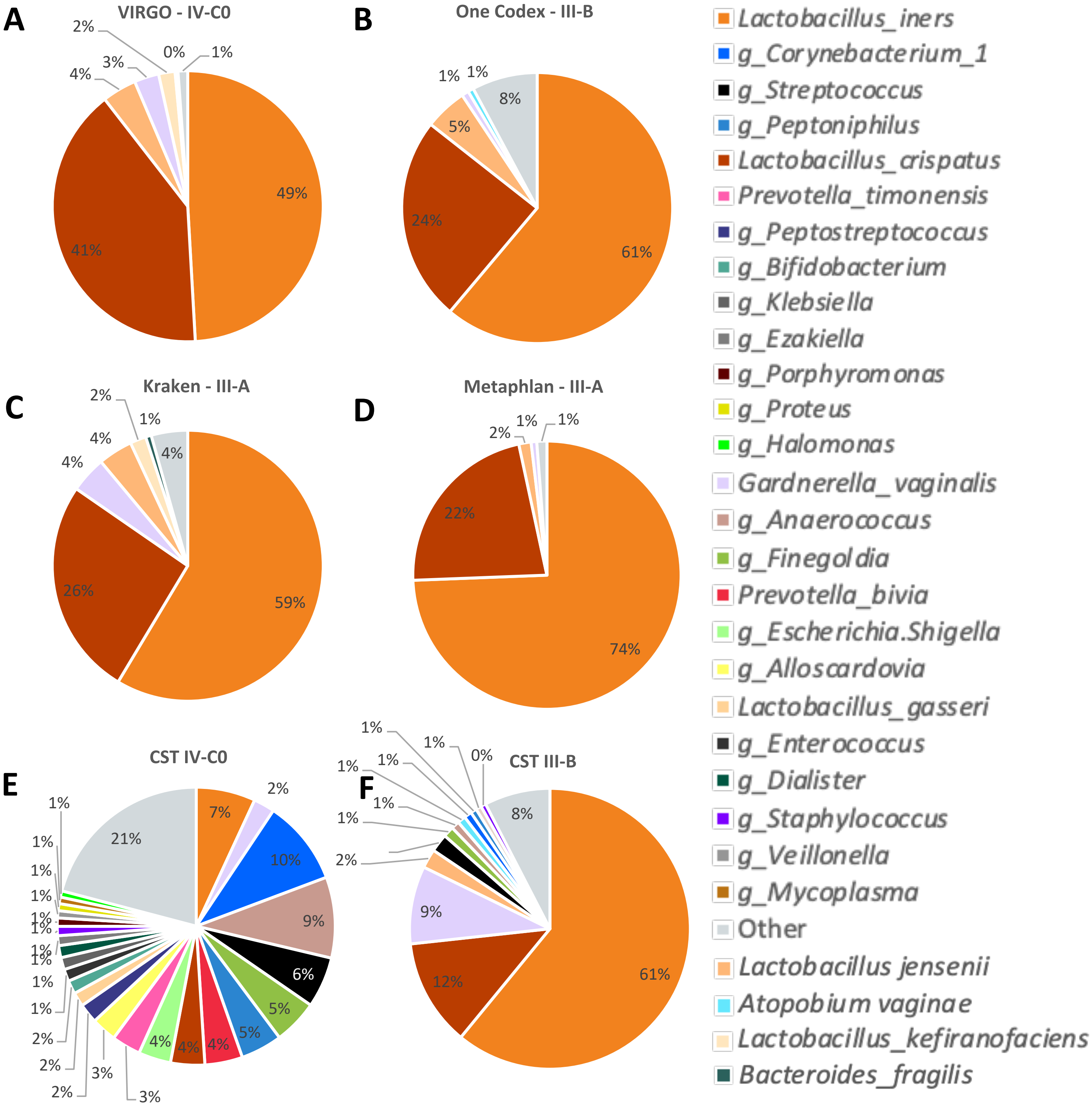

Supplementary Figure 5. Pie chart representation of Community State Types (CSTs) for patient sample 319C identified by the CST classifier VALENCIA based on the taxonomic outputs generated by four different databases: (A) VIRGO; (B) One Codex; (C) Kraken; and (D) Metaphlan. The reference bacterial compositions for CST III-B and IV-C0 are shown for comparison.

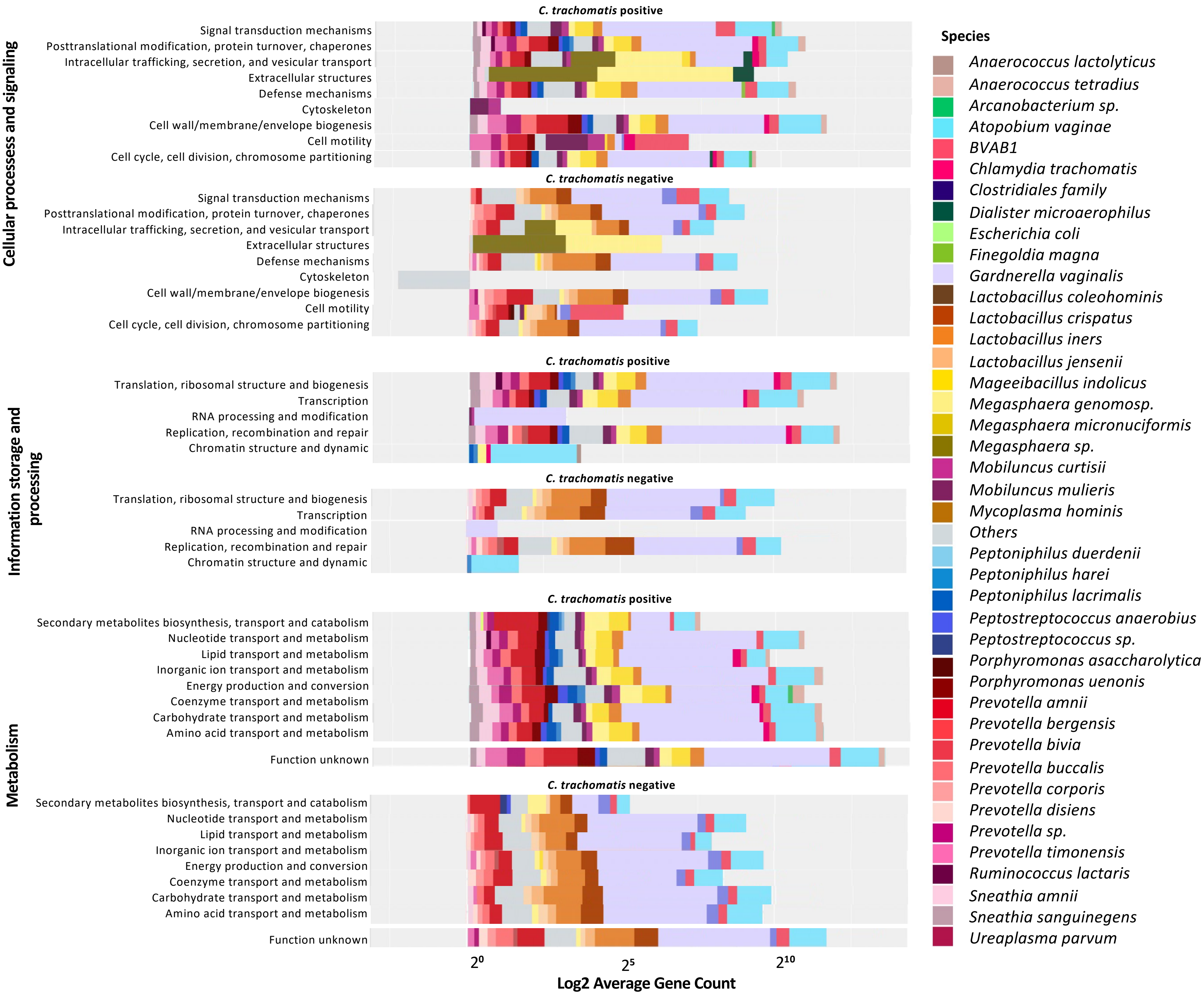

Supplementary Figure 6. Functional profiling of the endocervical metagenomes for *C. trachomatis* (*Ct*) positive and negative women. Functional categories were annotated using EggNOG v4.5 (see Material and Methods). Functional profiles were stratified by species using the taxonomic profiling provided by VIRGO. y-axis, functional categories; x-axis, Log 2 transformed gene counts for individual species.

Supplementary Table 1. Metagenomic shotgun sequencing (MSS) reads confirmed by BLASTn

Sample ID	*Ct* status	Sexually transmitted and genitorectal pathogens	# of reads*	Reads per million**
30C	*Ct-*	Human papillomavirus type 6b	2	
30R	*Ct-*	**Mycoplasma genitalium**	**191**	**18.01**
	Ct-	**Neisseria gonorrhoeae**	**67**	**6.32**
	Ct-	Human papillomavirus type 6b	1	
30V	*Ct-*	Mycoplasma genitalium	2	
	Ct-	Neisseria gonorrhoeae	1	
	Ct-	Human papillomavirus type 16	1	
35C	*Ct-*	**Human papillomavirus type 16**	**4613**	**164.49**
	Ct-	Human papillomavirus type 71	1	
35R	*Ct-*	**Mycoplasma genitalium**	**174**	**9.88**
	Ct-	**Neisseria gonorrhoeae**	**318**	**18.07**
	Ct-	**Human papillomavirus type 16**	**3844**	**218.37**
	Ct-	**Human papillomavirus type 26**	**28**	**1.59**
	Ct-	Human papillomavirus type 6b	10	
35V	*Ct-*	Mycoplasma genitalium	1	
	Ct-	Neisseria gonorrhoeae	3	
	Ct-	**Human papillomavirus type 6b**	**2943**	**76.56**
	Ct-	**Human papillomavirus type 16**	**219**	**5.7**
	Ct-	Candida albicans	16	0.42
57C	*Ct-*	**Mycoplasma genitalium**	**42**	**1.19**
	Ct-	Human papillomavirus type 16	1	
	Ct-	Chlamydia trachomatis	6	0.17
57R	*Ct-*	**Mycoplasma genitalium**	**58**	**4.09**
57V	*Ct-*	Mycoplasma genitalium	8	
	Ct-	Chlamydia trachomatis	12	0.37
	Ct-	Human papillomavirus type 6b	13	0.39
121C	*Ct-*	Human papillomavirus type 6b	1	
	Ct-	Candida albicans	4	
121R	*Ct-*	**Mycoplasma genitalium**	**36**	**11.04**
	Ct-	Human betaherpesvirus 5	2	
121V	*Ct-*	Human papillomavirus type 6b	1	
	Ct-	**Candida albicans**	**46**	**1.41**
192C	*Ct+*	**Mycoplasma genitalium**	**200**	**6.77**
	Ct+	Alphapapillomavirus 7	8	
	Ct+	Alphapapillomavirus 10	2	
	Ct+	Human papillomavirus type 6b	2	
	Ct+	Alphapapillomavirus 9	1	
	Ct+	Human papillomavirus type 16	1	
192R	*Ct+*	**Mycoplasma genitalium**	**65**	**4.1**
	Ct+	**Neisseria gonorrhoeae**	**59**	**3.72**

	Ct+	**Human papillomavirus type 54**	**155**	**9.78**
	Ct+	**Human papillomavirus type 53**	**30**	**1.89**
	Ct+	Human papillomavirus type 6b	6	
	Ct+	Alphapapillomavirus 7	4	
	Ct+	Human papillomavirus type 103	4	
192V	*Ct*+	Human betaherpesvirus 5	4	
	Ct+	**Mycoplasma genitalium**	**38**	**1.08**
	Ct+	**Alphapapillomavirus 7**	**107**	**3.04**
	Ct+	Human papillomavirus type 16	4	
319C	*Ct*-	Human papillomavirus type 16	2	
319R	*Ct*-	**Mycoplasma genitalium**	**25**	**5.64**
	Ct-	**Neisseria gonorrhoeae**	**25**	**5.64**
	Ct-	Human alphaherpesvirus 2	1	
	Ct-	Human papillomavirus type 16	4	
	Ct-	Candida albicans	4	
319V	*Ct*-	None		
72C	*Ct*+	Mycoplasma genitalium	4	
	Ct+	Human papillomavirus type 16	1	
72R	*Ct*+	**Mycoplasma genitalium**	**7**	**3.82**
	Ct+	Human papillomavirus type 7	4	
	Ct+	Human papillomavirus type 16	2	
72V	*Ct*+	Mycoplasma genitalium	7	0.23
	Ct+	Human papillomavirus type 7	4	
	Ct+	Human papillomavirus type 16	2	
98C	*Ct*+	None		
98R	*Ct*+	**Mycoplasma genitalium**	**162**	**10.3**
	Ct+	**Neisseria gonorrhoeae**	**19**	**1.2**
	Ct+	Human papillomavirus type 6b	1	
	Ct+	Human papillomavirus type 16	1	
98V	*Ct*+	Mycoplasma genitalium	2	
107C	*Ct*+	**Mycoplasma genitalium**	**44**	**1.3**
	Ct+	Neisseria gonorrhoeae	7	
	Ct+	Human papillomavirus type 6b	1	
107R	*Ct*+	**Mycoplasma genitalium**	**79**	**2.88**
	Ct+	Alphapapillomavirus	1	
	Ct+	Alphapapillomavirus 9	1	
	Ct+	Human papillomavirus type 16	1	
	Ct+	Alphapapillomavirus 4	1	
107V	*Ct*+	**Mycoplasma genitalium**	**148**	**6.39**
	Ct+	Neisseria gonorrhoeae	5	
	Ct+	Alphapapillomavirus	1	
	Ct+	Alphapapillomavirus 9	1	
	Ct+	Human papillomavirus type 16	1	

362C	*Ct*+	Mycoplasma genitalium	20	0.86
	Ct+	Alphapapillomavirus	2	
	Ct+	Alphapapillomavirus 9	2	
	Ct+	Human papillomavirus type 16	2	
362R	*Ct*+	**Mycoplasma genitalium**	**36**	**1.05**
	Ct+	Neisseria gonorrhoeae	1	
	Ct+	Human alphaherpesvirus 2	2	
	Ct+	Human papillomavirus type 16	2	
	Ct+	Alphapapillomavirus 10	1	
	Ct+	Human papillomavirus type 6b	1	
	Ct+	**Candida albicans**	**38**	**1.11**
362V	*Ct*+	Mycoplasma genitalium	15	0.47
	Ct+	Neisseria gonorrhoeae	1	
	Ct+	Human papillomavirus type 16	4	
	Ct+	Alphapapillomavirus 9	4	

*All reads confirmed by BLASTn

**Bold indicates putative presence of the pathogen based on >3 different regions of the genome represented by the reads; confirmed reads were divided by total microbial reads, excluding all human reads, and multiplied by a million to arrive at a value per million reads (RPM)

Supplementary Table 2. Metagenomic shotgun sequencing results.

No.	Sample ID	*Ct* status	DNA conc. (ng/μl)	Yield (Mbases)	No. of reads (all 3 lanes combined)	No. of human reads	hg38 / Raw*
1	72V	Pos	3.96	5,679	113,463,562	47,057,878	0.41474
2	72R	Pos	2.7	5,448	108,158,802	94,718,989	0.87574
3	72C	Pos	12.2	4,499	89,275,434	11,795,963	0.13213
4	98V	Pos	3.86	3,886	77,107,842	19,236,093	0.24947
5	98R	Pos	6.76	5,319	105,832,652	66,902,111	0.63215
6	98C	Pos	10.6	5,331	106,385,460	29,687,926	0.27906
7	107V	Pos	12.4	5,502	109,224,392	53,012,059	0.48535
8	107R	Pos	3.4	4,928	97,894,146	70,497,490	0.72014
9	107C	Pos	3.6	5,316	105,761,650	28,851,778	0.2728
10	192V	Pos	11.8	5,016	99,595,634	18,166,244	0.1824
11	192R	Pos	14.8	5,535	110,141,344	70,754,799	0.6424
12	192C	Pos	9.52	3,889	77,215,428	9,551,548	0.1237
13	362V	Pos	14	4,494	89,209,608	16,439,547	0.18428
14	362R	Pos	8.14	5,917	117,444,904	38,001,648	0.32357
15	362C	Pos	13.2	4,172	82,937,714	29,328,434	0.35362
16	57V	Neg	10.4	5,127	102,155,532	29,484,130	0.28862
17	57R	Neg	6.46	4,820	96,183,146	61,839,030	0.64293
18	57C	Neg	2.44	4,611	91,440,332	9,218,100	0.10081
19	35V	Neg	9.38	4,957	98,400,552	10,313,362	0.10481
20	35R	Neg	11	4,362	86,712,654	44,084,713	0.5084
21	35C	Neg	2.79	3,717	73,629,352	6,578,783	0.08935
22	30V	Neg	16.4	4,149	82,309,702	14,166,323	0.17211
23	30R	Neg	16	4,396	87,430,188	59,737,550	0.68326
24	30C	Neg	22	4,402	87,402,556	11,126,345	0.1273
25	121V	Neg	17.2	4,349	86,308,898	12,494,076	0.14476
26	121R	Neg	9.06	4,922	97,894,650	84,150,241	0.8596
27	121C	Neg	6.3	4,545	90,674,888	9130278	0.1007
28	319V	Neg	12	3,857	76,500,464	9,991,726	0.13061
29	319R	Neg	8.98	3,049	60,655,066	47,099,265	0.77651
30	319C	Neg	9.34	4,101	81,390,062	9,221,494	0.1133

*Mean endocervical human reads per raw reads: 0.245167; Mean vaginal human reads per raw reads: 0.235715; Mean rectal human reads per raw reads: 0.66647; difference in human to raw read ratio in rectum vs. vagina ($p < 0.0001$, One-way ANOVA) and rectum vs. endocervix ($p < 0.0001$, One-way ANOVA).

Supplementary Table 3. Metabolic pathways unique to the vaginal site based on the HUMAnN2 analysis.

# Pathway	107V	121V	192V	30V	319V	35V	362V	57V	72V	98V
PWY-7221: guanosine ribonucleotides de novo biosynthesis|g__Clostridiales_noname.s__Clostridiales_genomosp_BVAB3	0.0046	0	0.0044	0	0	0	0	0	0.0236	0
PWY-6703: preQ0 biosynthesis|g__Prevotella.s__Prevotella_buccalis	0.0000	0	0	0	0	0	0	0	0	0.0073
COA-PWY-1: coenzyme A biosynthesis II (mammalian)|g__Clostridiales_noname.s__Clostridiales_genomosp_BVAB3	0.0045	0	0	0	0	0	0	0	0	0
PWY-7197: pyrimidine deoxyribonucleotide phosphorylation|g__Mobiluncus.s__Mobiluncus_curtisii	0.0044	0	0	0	0	0	0	0	0	0
TRNA-CHARGING-PWY: tRNA charging|g__Clostridiales_noname.s__Clostridiales_genomosp_BVAB3	0.0041	0	0	0	0	0	0	0	0	0
PEPTIDOGLYCANSYN-PWY: peptidoglycan biosynthesis I (meso-diaminopimelate containing)|g__Clostridiales_noname.s__Clostridiales_genomosp_BVAB3	0.0037	0	0	0	0	0	0	0	0	0
PANTO-PWY: phosphopantothenate biosynthesis I|g__Mobiluncus.s__Mobiluncus_curtisii	0.0036	0	0	0	0	0	0	0	0	0
PWY-6121: 5-aminoimidazole ribonucleotide biosynthesis I|g__Mobiluncus.s__Mobiluncus_curtisii	0.0035	0	0	0	0	0	0	0	0	0
PWY-7221: guanosine ribonucleotides de novo biosynthesis|g__Mobiluncus.s__Mobiluncus_curtisii	0.0034	0	0	0	0	0	0	0	0	0
PWY-6387: UDP-N-acetylmuramoyl-pentapeptide biosynthesis I (meso-diaminopimelate containing)|g__Clostridiales_noname.s__Clostridiales_genomosp_BVAB3	0.0033	0	0	0	0	0	0	0	0	0
PWY-7357: thiamin formation from pyrithiamine and oxythiamine (yeast)|g__Clostridiales_noname.s__Clostridiales_genomosp_BVAB3	0.0033	0	0	0	0	0	0	0	0	0
NONOXIPENT-PWY: pentose phosphate pathway (non-oxidative branch)|g__Mobiluncus.s__Mobiluncus_curtisii	0.0032	0	0	0	0	0	0	0	0	0
PWY-7228: superpathway of guanosine nucleotides de novo biosynthesis I|g__Mobiluncus.s__Mobiluncus_curtisii	0.0031	0	0	0	0	0	0	0	0	0
PWY-6386: UDP-N-acetylmuramoyl-pentapeptide biosynthesis II (lysine-containing)|g__Mobiluncus.s__Mobiluncus_curtisii	0.0031	0	0	0	0	0	0	0	0	0
PWY-6151: S-adenosyl-L-methionine cycle I|g__Prevotella.s__Prevotella_buccalis	0.0000	0	0	0	0	0	0	0	0	0.0030
NONMEVIPP-PWY: methylerythritol phosphate pathway I|g__Clostridiales_noname.s__Clostridiales_genomosp_BVAB3	0.0028	0	0	0	0	0	0	0	0	0
PWY-6387: UDP-N-acetylmuramoyl-pentapeptide biosynthesis I (meso-diaminopimelate containing)|g__Mobiluncus.s__Mobiluncus_curtisii	0.0028	0	0	0	0	0	0	0	0	0
NONMEVIPP-PWY: methylerythritol phosphate pathway I|g__Mobiluncus.s__Mobiluncus_curtisii	0.0028	0	0	0	0	0	0	0	0	0
PEPTIDOGLYCANSYN-PWY: peptidoglycan biosynthesis I (meso-diaminopimelate containing)|g__Mobiluncus.s__Mobiluncus_curtisii	0.0028	0	0	0	0	0	0	0	0	0
PWY-5686: UMP biosynthesis|g__Mobiluncus.s__Mobiluncus_curtisii	0.0027	0	0	0	0	0	0	0	0	0
PWY-3001: superpathway of L-isoleucine biosynthesis I|g__Mobiluncus.s__Mobiluncus_curtisii	0.0024	0	0	0	0	0	0	0	0	0

PWY-7111: pyruvate fermentation to isobutanol (engineered)|g__Megasphaera.s__Megasphaera_sp_UPII_135_E	0.0023	0	0	0	0	0	0	0	0	0
PWY-5695: urate biosynthesis/inosine 5'-phosphate degradation|g__Prevotella.s__Prevotella_buccalis	0.0000	0	0	0	0	0	0	0	0	0.0023
COA-PWY-1: coenzyme A biosynthesis II (mammalian)|g__Mobiluncus.s__Mobiluncus_curtisii	0.0023	0	0	0	0	0	0	0	0	0
PWY-1269: CMP-3-deoxy-D-manno-octulosonate biosynthesis I|g__Megasphaera.s__Megasphaera_sp_UPII_135_E	0.0023	0	0	0	0	0	0	0	0	0
PANTO-PWY: phosphopantothenate biosynthesis I|g__Megasphaera.s__Megasphaera_sp_UPII_135_E	0.0023	0	0	0	0	0	0	0	0	0
THRESYN-PWY: superpathway of L-threonine biosynthesis|g__Mobiluncus.s__Mobiluncus_curtisii	0.0022	0	0	0	0	0	0	0	0	0
PWY-7199: pyrimidine deoxyribonucleosides salvage|g__Clostridiales_noname.s__Clostridiales_genomosp_BVAB3	0.0021	0	0	0	0	0	0	0	0	0
HISTSYN-PWY: L-histidine biosynthesis|g__Mobiluncus.s__Mobiluncus_curtisii	0.0021	0	0	0	0	0	0	0	0	0
PWY-6163: chorismate biosynthesis from 3-dehydroquinate|g__Megasphaera.s__Megasphaera_sp_UPII_135_E	0.0020	0	0	0	0	0	0	0	0	0
ARO-PWY: chorismate biosynthesis I|g__Megasphaera.s__Megasphaera_sp_UPII_135_E	0.0020	0	0	0	0	0	0	0	0	0
SER-GLYSYN-PWY: superpathway of L-serine and glycine biosynthesis I|g__Mobiluncus.s__Mobiluncus_curtisii	0.0020	0	0	0	0	0	0	0	0	0
PWY-7219: adenosine ribonucleotides de novo biosynthesis|g__Megasphaera.s__Megasphaera_sp_UPII_135_E	0.0020	0	0	0	0	0	0	0	0	0
PWY-6125: superpathway of guanosine nucleotides de novo biosynthesis II|g__Mobiluncus.s__Mobiluncus_curtisii	0.0019	0	0	0	0	0	0	0	0	0
COA-PWY: coenzyme A biosynthesis I|g__Mobiluncus.s__Mobiluncus_curtisii	0.0018	0	0	0	0	0	0	0	0	0
THRESYN-PWY: superpathway of L-threonine biosynthesis|g__Megasphaera.s__Megasphaera_sp_UPII_135_E	0.0016	0	0	0	0	0	0	0	0	0
CALVIN-PWY: Calvin-Benson-Bassham cycle|g__Megasphaera.s__Megasphaera_sp_UPII_135_E	0.0015	0	0	0	0	0	0	0	0	0
PWY-7221: guanosine ribonucleotides de novo biosynthesis|g__Megasphaera.s__Megasphaera_sp_UPII_135_E	0.0013	0	0	0	0	0	0	0	0	0
PWY-5686: UMP biosynthesis|g__Megasphaera.s__Megasphaera_sp_UPII_135_E	0.0013	0	0	0	0	0	0	0	0	0
PWY-7220: adenosine deoxyribonucleotides de novo biosynthesis II|g__Mobiluncus.s__Mobiluncus_curtisii	0.0012	0	0	0	0	0	0	0	0	0
PWY-7222: guanosine deoxyribonucleotides de novo biosynthesis II|g__Mobiluncus.s__Mobiluncus_curtisii	0.0012	0	0	0	0	0	0	0	0	0
PWY-6121: 5-aminoimidazole ribonucleotide biosynthesis I|g__Megasphaera.s__Megasphaera_sp_UPII_135_E	0.0012	0	0	0	0	0	0	0	0	0
PEPTIDOGLYCANSYN-PWY: peptidoglycan biosynthesis I (meso-diaminopimelate containing)|g__Megasphaera.s__Megasphaera_sp_UPII_135_E	0.0011	0	0	0	0	0	0	0	0	0

PWY-6386: UDP-N-acetylmuramoyl-pentapeptide biosynthesis II (lysine-containing)|g__Megasphaera.s__Megasphaera_sp_UPII_135_E	0.0010	0	0	0	0	0	0	0	0	0
PWY0-1296: purine ribonucleosides degradation|g__Megasphaera.s__Megasphaera_sp_UPII_135_E	0.0010	0	0	0	0	0	0	0	0	0
PWY-6122: 5-aminoimidazole ribonucleotide biosynthesis II|g__Megasphaera.s__Megasphaera_sp_UPII_135_E	0.0010	0	0	0	0	0	0	0	0	0
PWY-6277: superpathway of 5-aminoimidazole ribonucleotide biosynthesis|g__Megasphaera.s__Megasphaera_sp_UPII_135_E	0.0010	0	0	0	0	0	0	0	0	0
PWY-6387: UDP-N-acetylmuramoyl-pentapeptide biosynthesis I (meso-diaminopimelate containing)|g__Megasphaera.s__Megasphaera_sp_UPII_135_E	0.0010	0	0	0	0	0	0	0	0	0
PWY-1042: glycolysis IV (plant cytosol)|g__Megasphaera.s__Megasphaera_sp_UPII_135_E	0.0010	0	0	0	0	0	0	0	0	0
PWY-724: superpathway of L-lysine, L-threonine and L-methionine biosynthesis II|g__Megasphaera.s__Megasphaera_sp_UPII_135_E	0.0009	0	0	0	0	0	0	0	0	0
PWY-5695: urate biosynthesis/inosine 5'-phosphate degradation|g__Megasphaera.s__Megasphaera_sp_UPII_135_E	0.0009	0	0	0	0	0	0	0	0	0
COA-PWY-1: coenzyme A biosynthesis II (mammalian)|g__Megasphaera.s__Megasphaera_sp_UPII_135_E	0.0008	0	0	0	0	0	0	0	0	0
PWY-5097: L-lysine biosynthesis VI|g__Megasphaera.s__Megasphaera_sp_UPII_135_E	0.0007	0	0	0	0	0	0	0	0	0
PWY-6609: adenine and adenosine salvage III|unclassified	0.0007	0	0	0	0	0	0	0	0	0
P163-PWY: L-lysine fermentation to acetate and butanoate	0.0007	0	0	0	0	0	0	0	0	0
PWY-6897: thiamin salvage II|g__Megasphaera.s__Megasphaera_sp_UPII_135_E	0.0007	0	0	0	0	0	0	0	0	0
PWY-6609: adenine and adenosine salvage III|g__Megasphaera.s__Megasphaera_sp_UPII_135_E	0.0007	0	0	0	0	0	0	0	0	0
PWY-7357: thiamin formation from pyrithiamine and oxythiamine (yeast)|g__Megasphaera.s__Megasphaera_sp_UPII_135_E	0.0007	0	0	0	0	0	0	0	0	0
NONOXIPENT-PWY: pentose phosphate pathway (non-oxidative branch)|g__Peptoniphilus.s__Peptoniphilus_lacrimalis	0.0006	0	0	0	0	0	0	0	0	0
VALSYN-PWY: L-valine biosynthesis|g__Megasphaera.s__Megasphaera_sp_UPII_135_E	0.0005	0	0	0	0	0	0	0	0	0
PANTO-PWY: phosphopantothenate biosynthesis I|g__Peptostreptococcus.s__Peptostreptococcus_anaerobius	0.0005	0	0	0	0	0	0	0	0	0
PWY-6936: seleno-amino acid biosynthesis|g__Peptoniphilus.s__Peptoniphilus_harei	0.0005	0	0	0	0	0	0	0	0	0
PWY-7357: thiamin formation from pyrithiamine and oxythiamine (yeast)|g__Anaerococcus.s__Anaerococcus_tetradius	0.0005	0	0	0	0	0	0	0	0	0
PWY-6737: starch degradation V|g__Fusobacterium.s__Fusobacterium_nucleatum	0.0004	0	0	0	0	0	0	0	0	0

COA-PWY: coenzyme A biosynthesis Ilg__Anaerococcus.s__Anaerococcus_tetradius	0.0003	0	0	0	0	0	0	0	0	0
UDPNAGSYN-PWY: UDP-N-acetyl-D-glucosamine biosynthesis Ilg__Fusobacterium.s__Fusobacterium_nucleatum	0.0002	0	0	0	0	0	0	0	0	0

Supplementary Table 4. Functional pathways unique to the rectal site identified by HUManN2 from the Nephele pipeline.

# Pathway	121R	30R	319R	35R	57R	107R	192R	362R	72R	98R
1CMET2-PWY: N10-formyl-tetrahydrofolate biosynthesis	0.0135041	0.00312075	0.00862199	0	0.00595099	0.00229949	0.00276297	0	0.00737026	0.00810707
1CMET2-PWY: N10-formyl-tetrahydrofolate biosynthesis|g__Akkermansia.s__Akkermansia_muciniphila	0	0	0.00471296	0	0	0	0	0	0	0
1CMET2-PWY: N10-formyl-tetrahydrofolate biosynthesis|g__Bacteroides.s__Bacteroides_fragilis	0.00675324	0	0	0	0	0	0	0	0	0.00327906
AEROBACTINSYN-PWY: aerobactin biosynthesis	0	0	0.00030935	0	0	0	0	0	0	0.00018154
AEROBACTINSYN-PWY: aerobactin biosynthesis|g__Escherichia.s__Escherichia_coli	0	0	0.00030935	0	0	0	0	0	0	0.00018154
ANAGLYCOLYSIS-PWY: glycolysis III (from glucose)|g__Escherichia.s__Escherichia_coli	0	0	0.00036501	0	0	0	0	0	0	0
ARG+POLYAMINE-SYN: superpathway of arginine and polyamine biosynthesis	0.00058599	0	0.00132786	0	0.00170885	0	0.00481406	0	0	0.0008298
ARGININE-SYN4-PWY: L-ornithine de novo biosynthesis	0.0111131	0	0.00259763	0	0	0	0	0	0.00089456	0.00621895
ARGININE-SYN4-PWY: L-ornithine de novo biosynthesis|g__Bacteroides.s__Bacteroides_fragilis	0.00768261	0	0.00113561	0	0	0	0	0	0.00037038	0.00447226
ARGORNPROST-PWY: arginine, ornithine and proline interconversion	0.00063482	0	0	0.0009993	0	0	0	0	0	0.00062416
ARGSYN-PWY: L-arginine biosynthesis I (via L-ornithine)	0.00223317	0	0.00672642	0.00588981	0.00445275	0	0.00548457	0	0	0.00152828
ARGSYN-PWY: L-arginine biosynthesis I (via L-ornithine)|g__Blautia.s__Ruminococcus_torques	0	0	0	0	0	0	0.00021568	0	0	0
ARGSYN-PWY: L-arginine biosynthesis I (via L-ornithine)|g__Escherichia.s__Escherichia_coli	0	0	0.00026996	0	0.00036678	0	0	0	0	0
ARGSYNBSUB-PWY: L-arginine biosynthesis II (acetyl cycle)	0.00094614	0	0.00288943	0.00360221	0.00217849	0	0.00260483	0	0	0.00064254
ARGSYNBSUB-PWY: L-arginine biosynthesis II (acetyl cycle)|g__Bifidobacterium.s__Bifidobacterium_longum	0	0	0	0	0.00031282	0	0	0	0	0
ARO-PWY: chorismate biosynthesis I|g__Akkermansia.s__Akkermansia_muciniphila	0	0	0.00458838	0	0	0	0	0	0	0
ARO-PWY: chorismate biosynthesis I|g__Blautia.s__Ruminococcus_gnavus	0.00449358	0	0.00147301	0	0	0	0	0	0	0
ARO-PWY: chorismate biosynthesis I|g__Catenibacterium.s__Catenibacterium_mitsuokai	0	0.00048183	0	0	0	0	0.00055982	0	0	0
ARO-PWY: chorismate biosynthesis I|g__Escherichia.s__Escherichia_coli	0	0	0.00039513	0	0	0	0	0	0	0
ARO-PWY: chorismate biosynthesis I|g__Eubacterium.s__Eubacterium_rectale	0.00033887	0	0	0.00085926	0	0	0	0	0.00165831	0
ARO-PWY: chorismate biosynthesis I|g__Megamonas.s__Megamonas_rupellensis	0	0	0	0	0	0	0.00087712	0	0	0
ARO-PWY: chorismate biosynthesis I|g__Mitsuokella.s__Mitsuokella_multacida	0	0	0	0	0	0	0.00148904	0	0	0
ARO-PWY: chorismate biosynthesis I|unclassified	0	0.00656988	0	0.00392534	0	0	0.00382633	0	0.00173094	0
ASPASN-PWY: superpathway of L-aspartate and L-asparagine biosynthesis|g__Escherichia.s__Escherichia_coli	0.00019708	0.0003288	0.00049187	0	0.00108784	0	0	0	0	0.0003756
ASPASN-PWY: superpathway of L-aspartate and L-asparagine biosynthesis|g__Faecalibacterium.s__Faecalibacterium_prausnitzii	0	0.00085539	0	0.00054135	0.00031227	0	0.00090476	0	0.00198309	0.00162117
ASPASN-PWY: superpathway of L-aspartate and L-asparagine biosynthesis|g__Fusobacterium.s__Fusobacterium_gonidiaformans	0	0	0	0	0	0	0	0	0	0.00038422
ASPASN-PWY: superpathway of L-aspartate and L-asparagine biosynthesis|g__Peptoniphilus.s__Peptoniphilus_duerdenii	0	0	0	0	0	0.00084536	0	0	0	0
ASPASN-PWY: superpathway of L-aspartate and L-asparagine biosynthesis|g__Prevotella.s__Prevotella_buccalis	0	0	0.00022204	0	0	0	0	0	0.00076999	0
AST-PWY: L-arginine degradation II (AST pathway)	0	0	0.00037541	0	0	0	0	0	0	0

AST-PWY: L-arginine degradation II (AST pathway)|g__Escherichia.s__Escherichia_coli	0	0	0.00037541	0	0	0	0	0	0	0
BIOTIN-BIOSYNTHESIS-PWY: biotin biosynthesis I	0.00086575	0.0014734	0.00250489	0	0.00162089	0	0	0	0	0.00072907
BRANCHED-CHAIN-AA-SYN-PWY: superpathway of branched amino acid biosynthesis|g__Akkermansia.s__Akkermansia_muciniphila	0	0	0.0063822	0	0	0	0	0	0	0
BRANCHED-CHAIN-AA-SYN-PWY: superpathway of branched amino acid biosynthesis|g__Bifidobacterium.s__Bifidobacterium_longum	0.00020621	0	0	0	0	0	0	0	0	0
BRANCHED-CHAIN-AA-SYN-PWY: superpathway of branched amino acid biosynthesis|g__Desulfovibrio.s__Desulfovibrio_piger	0	0	0.00024741	0	0	0	0	0	0	0
BRANCHED-CHAIN-AA-SYN-PWY: superpathway of branched amino acid biosynthesis|g__Escherichia.s__Escherichia_coli	0	0	0	0	0.00074771	0	0	0	0	0
BRANCHED-CHAIN-AA-SYN-PWY: superpathway of branched amino acid biosynthesis|g__Faecalibacterium.s__Faecalibacterium_prausnitzii	0.00019274	0.00286752	0	0.00081645	0.00064364	0	0.00089729	0	0.00324497	0.00222094
BRANCHED-CHAIN-AA-SYN-PWY: superpathway of branched amino acid biosynthesis|g__Megamonas.s__Megamonas_hypermegale	0	0	0	0	0	0	0.0003392	0	0	0
BRANCHED-CHAIN-AA-SYN-PWY: superpathway of branched amino acid biosynthesis|g__Megamonas.s__Megamonas_rupellensis	0	0	0	0	0	0	0.00037342	0	0	0
BRANCHED-CHAIN-AA-SYN-PWY: superpathway of branched amino acid biosynthesis|g__Mitsuokella.s__Mitsuokella_multacida	0	0	0	0	0	0	0.00103741	0	0	0
CALVIN-PWY: Calvin-Benson-Bassham cycle|g__Escherichia.s__Escherichia_coli	0	0	0	0	0.00046659	0	0	0	0	0
CALVIN-PWY: Calvin-Benson-Bassham cycle|g__Faecalibacterium.s__Faecalibacterium_prausnitzii	0	0.00057188	0	0	0	0	0.00082002	0	0.00122405	0.00049286
CALVIN-PWY: Calvin-Benson-Bassham cycle|g__Megamonas.s__Megamonas_hypermegale	0	0	0	0	0	0	0.00144366	0	0	0
CALVIN-PWY: Calvin-Benson-Bassham cycle|unclassified	0	0	0	0	0	0	0	0	0.00161553	0
COA-PWY-1: coenzyme A biosynthesis II (mammalian)|g__Actinomyces.s__Actinomyces_turicensis	0	0	0	0	0	0	0	0	0.00068217	0
COA-PWY-1: coenzyme A biosynthesis II (mammalian)|g__Akkermansia.s__Akkermansia_muciniphila	0	0	0.00406136	0	0	0	0	0	0	0
COA-PWY-1: coenzyme A biosynthesis II (mammalian)|g__Anaerococcus.s__Anaerococcus_vaginalis	0.00019688	0	0	0	0	0	0	0	0	0
COA-PWY-1: coenzyme A biosynthesis II (mammalian)|g__Bacteroides.s__Bacteroides_fragilis	0.00770051	0	0.00046795	0	0	0	0	0	0.00027621	0.00420497
COA-PWY-1: coenzyme A biosynthesis II (mammalian)|g__Bacteroides.s__Bacteroides_vulgatus	0	0	0.00066661	0	0	0	0	0	0	0
COA-PWY-1: coenzyme A biosynthesis II (mammalian)|g__Bifidobacterium.s__Bifidobacterium_adolescentis	0.00153643	0	0	0.00262402	0.00447831	0	0	0	0.0026254	0
COA-PWY-1: coenzyme A biosynthesis II (mammalian)|g__Blautia.s__Ruminococcus_gnavus	0.0029304	0	0.0015182	0	0	0	0	0	0	0
COA-PWY-1: coenzyme A biosynthesis II (mammalian)|g__Blautia.s__Ruminococcus_torques	0	0	0	0	0	0	0.00061975	0	0	0
COA-PWY-1: coenzyme A biosynthesis II (mammalian)|g__Campylobacter.s__Campylobacter_ureolyticus	0	0	0	0	0.00054045	0	0	0	0	0
COA-PWY-1: coenzyme A biosynthesis II (mammalian)|g__Clostridium.s__Clostridium_nexile	0	0	0	0	0	0	0	0	0	0.00065444
COA-PWY-1: coenzyme A biosynthesis II (mammalian)|g__Collinsella.s__Collinsella_aerofaciens	0	0	0	0.00062496	0.00051849	0	0.00098971	0	0	0
COA-PWY-1: coenzyme A biosynthesis II (mammalian)|g__Desulfovibrio.s__Desulfovibrio_piger	0	0	0.00026823	0	0	0	0	0	0	0

COA-PWY-1: coenzyme A biosynthesis II (mammalian)|g__Dialister.s__Dialister_micraerophilus	0	0	0	0	0	0	0	0.00072192	0	0
COA-PWY-1: coenzyme A biosynthesis II (mammalian)|g__Eggerthella.s__Eggerthella_lenta	0	0	0.00035589	0	0	0	0	0	0	0
COA-PWY-1: coenzyme A biosynthesis II (mammalian)|g__Escherichia.s__Escherichia_coli	0	0.00042189	0.00072815	0	0.00098068	0	0	0	0	0
COA-PWY-1: coenzyme A biosynthesis II (mammalian)|g__Eubacterium.s__Eubacterium_rectale	0.00049005	0	0	0	0	0	0	0	0.00146585	0
COA-PWY-1: coenzyme A biosynthesis II (mammalian)|g__Faecalibacterium.s__Faecalibacterium_prausnitzii	0	0.00253287	0	0	0.00057274	0	0	0	0.00065947	0.00086021
COA-PWY-1: coenzyme A biosynthesis II (mammalian)|g__Fusobacterium.s__Fusobacterium_gonidiaformans	0	0	0	0	0	0	0	0	0	0.00045039
COA-PWY-1: coenzyme A biosynthesis II (mammalian)|g__Lachnospiraceae_noname.s__Lachnospiraceae_bacterium_2_1_58FAA	0	0	0.00094206	0	0	0	0	0	0	0
COA-PWY-1: coenzyme A biosynthesis II (mammalian)|g__Lactobacillus.s__Lactobacillus_ruminis	0.00048984	0	0	0	0.00075058	0	0	0	0	0
COA-PWY-1: coenzyme A biosynthesis II (mammalian)|g__Megasphaera.s__Megasphaera_elsdenii	0	0	0	0	0.00054098	0	0	0	0	0
COA-PWY-1: coenzyme A biosynthesis II (mammalian)|g__Mitsuokella.s__Mitsuokella_multacida	0	0	0	0.00073685	0	0	0.00081449	0	0	0
COA-PWY-1: coenzyme A biosynthesis II (mammalian)|g__Phascolarctobacterium.s__Phascolarctobacterium_succinatutens	0	0.00053114	0	0	0	0	0	0	0	0
COA-PWY-1: coenzyme A biosynthesis II (mammalian)|g__Prevotella.s__Prevotella_disiens	0.00028779	0	0	0	0	0	0	0	0	0
COA-PWY-1: coenzyme A biosynthesis II (mammalian)|unclassified	0	0.00530561	0	0	0.00185357	0.0128915	0.00169496	0	0.00200114	0
COA-PWY: coenzyme A biosynthesis I|g__Akkermansia.s__Akkermansia_muciniphila	0	0	0.00425815	0	0	0	0	0	0	0
COA-PWY: coenzyme A biosynthesis I|g__Anaerococcus.s__Anaerococcus_vaginalis	0.00015749	0	0	0	0	0	0	0	0	0
COA-PWY: coenzyme A biosynthesis I|g__Blautia.s__Ruminococcus_torques	0	0	0	0	0	0	0.00059717	0	0	0
COA-PWY: coenzyme A biosynthesis I|g__Desulfovibrio.s__Desulfovibrio_piger	0	0	0.00031281	0	0	0	0	0	0	0
COA-PWY: coenzyme A biosynthesis I|g__Dialister.s__Dialister_micraerophilus	0	0	0	0	0	0	0	0.00064879	0	0
COA-PWY: coenzyme A biosynthesis I|g__Eggerthella.s__Eggerthella_lenta	0	0	0.00032025	0	0	0	0	0	0	0
COA-PWY: coenzyme A biosynthesis I|g__Escherichia.s__Escherichia_coli	0.00031201	0.00031338	0.00066294	0	0.00088949	0	0	0	0	0
COA-PWY: coenzyme A biosynthesis I|g__Faecalibacterium.s__Faecalibacterium_prausnitzii	0	0.000841	0	0	0.00028667	0	0	0	0.00047561	0.00069818
COA-PWY: coenzyme A biosynthesis I|g__Fusobacterium.s__Fusobacterium_gonidiaformans	0	0	0	0	0	0.00023442	0	0	0	0.00040312
COA-PWY: coenzyme A biosynthesis I|g__Phascolarctobacterium.s__Phascolarctobacterium_succinatutens	0	0.00082035	0	0	0	0	0	0	0	0
COA-PWY: coenzyme A biosynthesis I|g__Prevotella.s__Prevotella_disiens	0.00028779	0	0	0	0.00104254	0	0	0	0	0
COBALSYN-PWY: adenosylcobalamin salvage from cobinamide I	0.00128435	0.00181708	0.00125303	0	0.00348163	0	0.00260643	0	0.00422861	0.00190364
COBALSYN-PWY: adenosylcobalamin salvage from cobinamide I|g__Escherichia.s__Escherichia_coli	0	0	0	0	0.00039232	0	0	0	0	0
COBALSYN-PWY: adenosylcobalamin salvage from cobinamide I|g__Eubacterium.s__Eubacterium_rectale	0	0	0	0	0.00060994	0	0	0	0.00160417	0
COBALSYN-PWY: adenosylcobalamin salvage from cobinamide I|g__Faecalibacterium.s__Faecalibacterium_prausnitzii	0	0.00079393	0	0	0	0	0	0	0.00137786	0
COBALSYN-PWY: adenosylcobalamin salvage from cobinamide I|g__Fusobacterium.s__Fusobacterium_gonidiaformans	0.00019124	0	0	0	0	0	0	0	0	0

COBALSYN-PWY: adenosylcobalamin salvage from cobinamide|g__Megamonas.s__Megamonas_rupellensis	0	0	0	0	0	0	0.00128662	0	0	0
COLANSYN-PWY: colanic acid building blocks biosynthesis|g__Escherichia.s__Escherichia_coli	0	0	0	0	0	0	0	0	0	0.00018984
COMPLETE-ARO-PWY: superpathway of aromatic amino acid biosynthesis|g__Akkermansia.s__Akkermansia_muciniphila	0	0	0.00504372	0	0	0	0	0	0	0
COMPLETE-ARO-PWY: superpathway of aromatic amino acid biosynthesis|g__Blautia.s__Ruminococcus_gnavus	0.0044635	0	0.00153806	0	0	0	0	0	0	0
COMPLETE-ARO-PWY: superpathway of aromatic amino acid biosynthesis|g__Escherichia.s__Escherichia_coli	0	0	0.00042562	0	0	0	0	0	0	0
COMPLETE-ARO-PWY: superpathway of aromatic amino acid biosynthesis|g__Eubacterium.s__Eubacterium_rectale	0.00036801	0	0	0.00088815	0	0	0	0	0.00177161	0
COMPLETE-ARO-PWY: superpathway of aromatic amino acid biosynthesis|g__Megamonas.s__Megamonas_rupellensis	0	0	0	0	0	0	0.00091926	0	0	0
COMPLETE-ARO-PWY: superpathway of aromatic amino acid biosynthesis|g__Mitsuokella.s__Mitsuokella_multacida	0	0	0	0	0	0	0.00154096	0	0	0
COMPLETE-ARO-PWY: superpathway of aromatic amino acid biosynthesis|unclassified	0	0.00571172	0	0.00373739	0	0	0.00390005	0	0	0
CRNFORCAT-PWY: creatinine degradation I	0	0	0	0	0	0	0	0	0.00044072	0
DAPLYSINESYN-PWY: L-lysine biosynthesis I	0.0014041	0.0086482	0.0011166	0.00785853	0.00432963	0	0.00227913	0	0.00577585	0.00069916
DAPLYSINESYN-PWY: L-lysine biosynthesis I|unclassified	0	0.00456291	0	0	0	0	0	0	0	0
DENOVOPURINE2-PWY: superpathway of purine nucleotides de novo biosynthesis II	0.00208825	0	0.00076211	0	0.00217805	0.0026216	0	0	0.00368927	0.00170844
DTDPRHAMSYN-PWY: dTDP-L-rhamnose biosynthesis I|g__Bacteroides.s__Bacteroides_fragilis	0.00944625	0	0.00190223	0	0	0	0	0	0	0.00802023
DTDPRHAMSYN-PWY: dTDP-L-rhamnose biosynthesis I|g__Eubacterium.s__Eubacterium_rectale	0.00033829	0	0.00020129	0.00076146	0.00033718	0	0	0	0.00131194	0
DTDPRHAMSYN-PWY: dTDP-L-rhamnose biosynthesis I|g__Faecalibacterium.s__Faecalibacterium_prausnitzii	0.00018114	0.00247148	0	0.00063824	0.00066153	0	0.00076839	0	0.00258753	0.00115891
DTDPRHAMSYN-PWY: dTDP-L-rhamnose biosynthesis I|g__Fusobacterium.s__Fusobacterium_gonidiaformans	0	0	0	0	0	0	0	0	0	0.00036582
DTDPRHAMSYN-PWY: dTDP-L-rhamnose biosynthesis I|g__Methanobrevibacter.s__Methanobrevibacter_smithii	0.00037886	0	0	0	0	0	0	0	0	0
DTDPRHAMSYN-PWY: dTDP-L-rhamnose biosynthesis I|g__Prevotella.s__Prevotella_buccalis	0	0	0	0	0	0.00044951	0	0	0.00047895	0
DTDPRHAMSYN-PWY: dTDP-L-rhamnose biosynthesis I|g__Prevotella.s__Prevotella_disiens	0.00033835	0	0	0	0	0	0	0	0	0
DTDPRHAMSYN-PWY: dTDP-L-rhamnose biosynthesis I|unclassified	0	0.00272255	0	0.00213199	0	0	0.00132678	0	0.00181234	0.00068913
ECASYN-PWY: enterobacterial common antigen biosynthesis	0	0	0.00032616	0	0	0	0	0	0	0
ECASYN-PWY: enterobacterial common antigen biosynthesis|g__Escherichia.s__Escherichia_coli	0	0	0.00029471	0	0	0	0	0	0	0
ENTBACSYN-PWY: enterobactin biosynthesis	0.00082769	0	0.0020078	0	0	0	0	0	0	0
ENTBACSYN-PWY: enterobactin biosynthesis|g__Escherichia.s__Escherichia_coli	0	0	0.0011819	0	0	0	0	0	0	0
FAO-PWY: fatty acid β-oxidation I	0	0.00042208	0.00014997	0.00050968	0	0	0	0	0.00031832	0.00044935
FAO-PWY: fatty acid β-oxidation I|g__Escherichia.s__Escherichia_coli	0	0	0.00014872	0.0002887	0	0	0	0	0	0
FASYN-ELONG-PWY: fatty acid elongation -- saturated	0.00089815	0.00239542	0.00224442	0	0.00184857	0	0.00078369	0	0	0.0006724
FASYN-ELONG-PWY: fatty acid elongation -- saturated|g__Escherichia.s__Escherichia_coli	0	0	0	0	0.00060598	0	0	0	0	0

FASYN-INITIAL-PWY: superpathway of fatty acid biosynthesis initiation (E. coli)	0.00059246	0.00169766	0.00186439	0	0.00137186	0	0.00050869	0	0	0.00043755
FASYN-INITIAL-PWY: superpathway of fatty acid biosynthesis initiation (E. coli)|g__Escherichia.s__Escherichia_coli	0	0	0	0	0.00039331	0	0	0	0	0.0002842
FERMENTATION-PWY: mixed acid fermentation	0.00050661	0	0.0007643	0.00196272	0.00128418	0	0	0	0	0.00073783
FUC-RHAMCAT-PWY: superpathway of fucose and rhamnose degradation	0	0	0.00170764	0	0.00065112	0	0	0	0	0.00084488
FUC-RHAMCAT-PWY: superpathway of fucose and rhamnose degradation|g__Escherichia.s__Escherichia_coli	0	0	0.00078678	0	0.00047381	0	0	0	0	0
FUCCAT-PWY: fucose degradation	0	0.000867	0.00175171	0	0.00108493	0	0.00064461	0	0	0.0005536
FUCCAT-PWY: fucose degradation|g__Escherichia.s__Escherichia_coli	0	0.00025467	0.00067121	0	0.00050837	0	0	0	0	0.00018329
GALACT-GLUCUROCAT-PWY: superpathway of hexuronide and hexuronate degradation	0.00058267	0.00297671	0.00108696	0.00253505	0.00179554	0	0.00229413	0	0.00360389	0.00313967
GALACT-GLUCUROCAT-PWY: superpathway of hexuronide and hexuronate degradation|g__Escherichia.s__Escherichia_coli	0	0	0.00032426	0	0	0	0	0	0	0
GALACT-GLUCUROCAT-PWY: superpathway of hexuronide and hexuronate degradation|g__Faecalibacterium.s__Faecalibacterium_prausnitzii	0.00013458	0.00144592	0	0.00059656	0.0003108	0	0.00111516	0	0.00215212	0.00128881
GALACTARDEG-PWY: D-galactarate degradation I	0	0	0	0	0.00037613	0	0	0	0	0
GALACTARDEG-PWY: D-galactarate degradation I|g__Escherichia.s__Escherichia_coli	0	0	0	0	0.00037613	0	0	0	0	0
GALACTUROCAT-PWY: D-galacturonate degradation I	0.00063103	0.002863	0.00075764	0.00210835	0.00178874	0	0.00191147	0	0.00371749	0.00316809
GALACTUROCAT-PWY: D-galacturonate degradation I|g__Escherichia.s__Escherichia_coli	0	0	0.00049884	0	0.00029305	0	0	0	0	0
GALACTUROCAT-PWY: D-galacturonate degradation I|g__Faecalibacterium.s__Faecalibacterium_prausnitzii	0.00014993	0.00189211	0	0.00117181	0.0004449	0	0.00117009	0	0.00249199	0.00165598
GLCMANNANAUT-PWY: superpathway of N-acetylglucosamine, N-acetylmannosamine and N-acetylneuraminate degradation	0.00523191	0.00196756	0.00434661	0.00255832	0.00264081	0.00263715	0.0046028	0	0.00458441	0.00388843
GLCMANNANAUT-PWY: superpathway of N-acetylglucosamine, N-acetylmannosamine and N-acetylneuraminate degradation|g__Dorea.s__Dorea_longicatena	0	0	0	0	0	0	0	0	0.00020615	0
GLCMANNANAUT-PWY: superpathway of N-acetylglucosamine, N-acetylmannosamine and N-acetylneuraminate degradation|g__Escherichia.s__Escherichia_coli	0.00019336	0.00048732	0.00092954	0	0	0	0	0	0	0
GLCMANNANAUT-PWY: superpathway of N-acetylglucosamine, N-acetylmannosamine and N-acetylneuraminate degradation|g__Faecalibacterium.s__Faecalibacterium_prausnitzii	0	0.0008019	0	0	0.0006796	0	0	0	0.00196805	0.00143594
GLCMANNANAUT-PWY: superpathway of N-acetylglucosamine, N-acetylmannosamine and N-acetylneuraminate degradation|g__Megamonas.s__Megamonas_rupellensis	0	0	0	0	0	0	0.00080568	0	0	0
GLUCARDEG-PWY: D-glucarate degradation I	0	0	0.00049538	0	0.00032486	0	0	0	0	0
GLUCARDEG-PWY: D-glucarate degradation I|g__Escherichia.s__Escherichia_coli	0	0	0.00049538	0	0.00032486	0	0	0	0	0
GLUCARGALACTSUPER-PWY: superpathway of D-glucarate and D-galactarate degradation	0	0	0	0	0.00037613	0	0	0	0	0
GLUCARGALACTSUPER-PWY: superpathway of D-glucarate and D-galactarate degradation|g__Escherichia.s__Escherichia_coli	0	0	0	0	0.00037613	0	0	0	0	0
GLUCONEO-PWY: gluconeogenesis I	0	0.00240595	0	0.00164278	0.00122758	0	0	0	0.00214319	0.00124888
GLUCOSE1PMETAB-PWY: glucose and glucose-1-phosphate degradation	0.0002801	0.00038477	0.00057681	0	0.00090385	0	0	0	0	0.00077611
GLUCOSE1PMETAB-PWY: glucose and glucose-1-phosphate degradation|g__Escherichia.s__Escherichia_coli	0	0.00026006	0.00056477	0	0.00022758	0	0	0	0	0.00032863
GLUCUROCAT-PWY: superpathway of β-D-glucuronide and D-glucuronate degradation	0.00052771	0.00456324	0.00143709	0.00273915	0.00183771	0	0.00308455	0	0.00483037	0.00393205

GLUCUROCAT-PWY: superpathway of β-D-glucuronide and D-glucuronate degradation|g__Escherichia.s__Escherichia_coli	0	0	0.00052134	0	0	0	0	0	0	0
GLUCUROCAT-PWY: superpathway of β-D-glucuronide and D-glucuronate degradation|g__Faecalibacterium.s__Faecalibacterium_prausnitzii	0.00012098	0.0014079	0	0.00096822	0.00036176	0	0.00115866	0	0.00219152	0.00129142
GLUDEG-I-PWY: GABA shunt	0.00087321	0	0.00139195	0	0	0	0	0	0.00036399	0.00061103
GLUTORN-PWY: L-ornithine biosynthesis	0.00055721	0	0.00269525	0.00254735	0.0013476	0	0.00192253	0	0	0.00037563
GLUTORN-PWY: L-ornithine biosynthesis|g__Blautia.s__Ruminococcus_torques	0	0	0	0	0	0	0.00036663	0	0	0
GLUTORN-PWY: L-ornithine biosynthesis|g__Escherichia.s__Escherichia_coli	0	0	0.00034184	0	0.00049827	0	0	0	0	0.00021677
GLYCOCAT-PWY: glycogen degradation I (bacterial)	0.00039809	0	0.00100103	0	0.00151747	0	0.00172626	0	0	0.00103527
GLYCOGENSYNTH-PWY: glycogen biosynthesis I (from ADP-D-Glucose)|g__Erysipelotrichaceae_noname.s__Eubacterium_biforme	0	0.00030569	0	0	0	0	0.00020338	0	0	0
GLYCOGENSYNTH-PWY: glycogen biosynthesis I (from ADP-D-Glucose)|g__Escherichia.s__Escherichia_coli	0	0	0.00049321	0	0	0	0	0	0	0
GLYCOGENSYNTH-PWY: glycogen biosynthesis I (from ADP-D-Glucose)|g__Eubacterium.s__Eubacterium_rectale	0.00059784	0	0	0.00159442	0.00042185	0	0	0	0.00223412	0.00085612
GLYCOL-GLYOXDEG-PWY: superpathway of glycol metabolism and degradation	0.00018332	0	0.00123389	0	0.00028945	0	0	0	0	0.0004148
GLYCOL-GLYOXDEG-PWY: superpathway of glycol metabolism and degradation|g__Escherichia.s__Escherichia_coli	0.00013792	0	0.00066947	0	0.00028046	0	0	0	0	0.00038394
GLYCOLYSIS-E-D: superpathway of glycolysis and Entner-Doudoroff	0.0014944	0.00080466	0.00120203	0.00121635	0.00300006	0	0	0	0.00068315	0.00123657
GLYCOLYSIS-TCA-GLYOX-BYPASS: superpathway of glycolysis, pyruvate dehydrogenase, TCA, and glyoxylate bypass	0.00055882	0.00139608	0.00185399	0.0016567	0.00199836	0	0	0	0	0.00164861
GLYOXYLATE-BYPASS: glyoxylate cycle	0.00067097	0.00066165	0.00096277	0.00125181	0.00095182	0	0	0	0	0.00064056
GLYOXYLATE-BYPASS: glyoxylate cycle|g__Escherichia.s__Escherichia_coli	0	0	0.00078341	0.00046947	0.00081996	0	0	0	0	0.00028865
GOLPDLCAT-PWY: superpathway of glycerol degradation to 1,3-propanediol	0	0	0	0	0	0	0.0018609	0	0	0
GOLPDLCAT-PWY: superpathway of glycerol degradation to 1,3-propanediol|g__Megamonas.s__Megamonas_hypermegale	0	0	0	0	0	0	0.00037008	0	0	0
HEMESYN2-PWY: heme biosynthesis II (anaerobic)|g__Escherichia.s__Escherichia_coli	0.00036543	0	0.00057303	0.00042459	0	0	0	0	0	0
HEXITOLDEGSUPER-PWY: superpathway of hexitol degradation (bacteria)	0.00118494	0.00192383	0.00243495	0.00104834	0.00282796	0	0.00047618	0	0	0.00336562
HISDEG-PWY: L-histidine degradation I|g__Alistipes.s__Alistipes_shahii	0	0	0	0	0.00021712	0	0	0	0	0
HISDEG-PWY: L-histidine degradation I|g__Clostridium.s__Clostridium_symbiosum	0	0	0.00012707	0	0	0	0	0	0	0
HISDEG-PWY: L-histidine degradation I|g__Fusobacterium.s__Fusobacterium_gonidiaformans	0.00020779	0	0	0.00027853	0.00015424	0.00042744	0	0	0	0.00100516
HISTSYN-PWY: L-histidine biosynthesis|g__Bifidobacterium.s__Bifidobacterium_adolescentis	0.00137361	0	0	0.00170803	0.00338166	0	0	0	0.00155695	0
HISTSYN-PWY: L-histidine biosynthesis|g__Escherichia.s__Escherichia_coli	0	0	0.00031386	0	0.00045073	0	0	0	0	0
HOMOSER-METSYN-PWY: L-methionine biosynthesis I	0.00456963	0.00143335	0.00350752	0.00634855	0.00463854	0	0.00342551	0	0.00478345	0.00242179
HOMOSER-METSYN-PWY: L-methionine biosynthesis I|g__Escherichia.s__Escherichia_coli	0	0	0.00068309	0	0.00022343	0	0	0	0	0
HSERMETANA-PWY: L-methionine biosynthesis III|g__Methanobrevibacter.s__Methanobrevibacter_smithii	0	0	0.00039448	0	0	0	0	0	0	0
ILEUSYN-PWY: L-isoleucine biosynthesis I (from threonine)|g__Akkermansia.s__Akkermansia_muciniphila	0	0	0.00660927	0	0	0	0	0	0	0
ILEUSYN-PWY: L-isoleucine biosynthesis I (from threonine)|g__Bifidobacterium.s__Bifidobacterium_longum	0.00025461	0	0	0	0	0	0	0	0	0

ILEUSYN-PWY: L-isoleucine biosynthesis I (from threonine)lg__Desulfovibrio.s__Desulfovibrio_piger	0	0	0.00024669	0	0	0	0	0	0	0
ILEUSYN-PWY: L-isoleucine biosynthesis I (from threonine)lg__Escherichia.s__Escherichia_coli	0	0	0	0	0.00082344	0	0	0	0	0
ILEUSYN-PWY: L-isoleucine biosynthesis I (from threonine)lg__Faecalibacterium.s__Faecalibacterium_prausnitzii	0.0002131	0.00303228	0	0.00093488	0.00074141	0	0.00093202	0	0.00365944	0.00251559
ILEUSYN-PWY: L-isoleucine biosynthesis I (from threonine)lg__Megamonas.s__Megamonas_hypermegale	0	0	0	0	0	0	0.00040219	0	0	0
ILEUSYN-PWY: L-isoleucine biosynthesis I (from threonine)lg__Megamonas.s__Megamonas_rupellensis	0	0	0	0	0	0	0.0004512	0	0	0
ILEUSYN-PWY: L-isoleucine biosynthesis I (from threonine)lg__Mitsuokella.s__Mitsuokella_multacida	0	0	0	0	0	0	0.00111117	0	0	0
KDO-NAGLIPASYN-PWY: superpathway of (Kdo)2-lipid A biosynthesis	0	0	0.00048885	0	0.00050682	0	0	0	0	0
KETOGLUCONMET-PWY: ketogluconate metabolism	0.00027962	0	0.00160783	0	0	0	0	0	0	0
LACTOSECAT-PWY: lactose and galactose degradation Ilg__Enterococcus.s__Enterococcus_faecium	0.0003013	0	0	0	0	0	0	0	0	0
LACTOSECAT-PWY: lactose and galactose degradation Ilg__Lactobacillus.s__Lactobacillus_ruminis	0.0007878	0	0	0	0.00025265	0	0	0	0	0
LPSSYN-PWY: superpathway of lipopolysaccharide biosynthesis	0	0	0.00070076	0	0.00074844	0	0	0	0	0
MET-SAM-PWY: superpathway of S-adenosyl-L-methionine biosynthesis	0.00777562	0.00268502	0.00517178	0.00886157	0.00735626	0	0.00589929	0	0.00800013	0.00413439
MET-SAM-PWY: superpathway of S-adenosyl-L-methionine biosynthesislg__Escherichia.s__Escherichia_coli	0	0	0.00023501	0	0	0	0	0	0	0
METHANOGENESIS-PWY: methanogenesis from H2 and CO2	0.00029415	0	0.0003282	0	0	0	0	0	0	0
METHANOGENESIS-PWY: methanogenesis from H2 and CO2lg__Methanobrevibacter.s__Methanobrevibacter_smithii	0.00029415	0	0.0003282	0	0	0	0	0	0	0
METHGLYUT-PWY: superpathway of methylglyoxal degradation	0.00016428	0	0	0	0.00029093	0	0	0	0	0.00043556
METSYN-PWY: L-homoserine and L-methionine biosynthesis	0.00715558	0.00254562	0.00563796	0.00798672	0.0065679	0	0.00567222	0	0.00721265	0.00390901
METSYN-PWY: L-homoserine and L-methionine biosynthesislg__Escherichia.s__Escherichia_coli	0	0	0.00027091	0	0.00030951	0	0	0	0	0
NAD-BIOSYNTHESIS-II: NAD salvage pathway II	0.00031957	0.00078687	0.00080024	0.00107152	0	0	0	0	0.00025655	0
NAD-BIOSYNTHESIS-II: NAD salvage pathway IIlg__Escherichia.s__Escherichia_coli	0.00029108	0.00055904	0.00076419	0.00095531	0	0	0	0	0	0
NAGLIPASYN-PWY: lipid IVA biosynthesis	0	0.00069437	0.00157316	0.00225361	0.00142742	0	0	0	0	0
NAGLIPASYN-PWY: lipid IVA biosynthesislg__Escherichia.s__Escherichia_coli	0	0	0	0.00053494	0.00060855	0	0	0	0	0
NONMEVIPP-PWY: methylerythritol phosphate pathway Ilg__Actinomyces.s__Actinomyces_turicensis	0	0	0	0	0	0.00176492	0	0	0.0006065	0
NONMEVIPP-PWY: methylerythritol phosphate pathway Ilg__Akkermansia.s__Akkermansia_muciniphila	0	0	0.00477298	0	0	0	0	0	0	0
NONMEVIPP-PWY: methylerythritol phosphate pathway Ilg__Bacteroides.s__Bacteroides_vulgatus	0	0	0.00084709	0	0.00096859	0	0	0	0	0
NONMEVIPP-PWY: methylerythritol phosphate pathway Ilg__Bifidobacterium.s__Bifidobacterium_adolescentis	0.00196472	0	0.00029778	0.00406025	0.00740285	0	0.00102968	0	0.00317838	0
NONMEVIPP-PWY: methylerythritol phosphate pathway Ilg__Blautia.s__Ruminococcus_gnavus	0.00412535	0	0	0	0	0	0	0	0	0
NONMEVIPP-PWY: methylerythritol phosphate pathway Ilg__Catenibacterium.s__Catenibacterium_mitsuokai	0.00036393	0	0	0	0.00065862	0	0	0	0	0
NONMEVIPP-PWY: methylerythritol phosphate pathway Ilg__Eubacterium.s__Eubacterium_rectale	0.00031201	0	0	0	0	0	0	0	0.00135739	0.00034414
NONMEVIPP-PWY: methylerythritol phosphate pathway Ilg__Lachnospiraceae_noname.s__Lachnospiraceae_bacterium_2_1_58FAA	0	0	0.00121802	0	0	0	0	0	0	0

NONMEVIPP-PWY: methylerythritol phosphate pathway I|g__Mitsuokella.s__Mitsuokella_multacida	0	0	0	0	0	0	0.00194919	0	0	0
NONMEVIPP-PWY: methylerythritol phosphate pathway I|g__Peptostreptococcus.s__Peptostreptococcus_anaerobius	0	0	0	0	0	0	0	0	0	0.00042475
NONMEVIPP-PWY: methylerythritol phosphate pathway I|unclassified	0	0.0063914	0	0	0	0	0.00366086	0	0	0
NONOXIPENT-PWY: pentose phosphate pathway (non-oxidative branch)|g__Blautia.s__Ruminococcus_gnavus	0.00455828	0	0.00114391	0	0	0	0	0	0	0
NONOXIPENT-PWY: pentose phosphate pathway (non-oxidative branch)|g__Blautia.s__Ruminococcus_torques	0	0	0	0	0	0	0.00102212	0	0	0
NONOXIPENT-PWY: pentose phosphate pathway (non-oxidative branch)|g__Campylobacter.s__Campylobacter_hominis	0	0	0	0.00040516	0	0	0	0	0	0
NONOXIPENT-PWY: pentose phosphate pathway (non-oxidative branch)|g__Escherichia.s__Escherichia_coli	0	0	0	0	0.00063767	0	0	0	0	0
NONOXIPENT-PWY: pentose phosphate pathway (non-oxidative branch)|g__Eubacterium.s__Eubacterium_siraeum	0	0	0	0	0	0	0	0	0.00020663	0
NONOXIPENT-PWY: pentose phosphate pathway (non-oxidative branch)|g__Faecalibacterium.s__Faecalibacterium_prausnitzii	0	0	0	0	0	0	0	0	0.00298405	0.00182316
NONOXIPENT-PWY: pentose phosphate pathway (non-oxidative branch)|g__Finegoldia.s__Finegoldia_magna	0	0.00019769	0	0	0	0	0	0	0	0
NONOXIPENT-PWY: pentose phosphate pathway (non-oxidative branch)|g__Megamonas.s__Megamonas_hypermegale	0	0	0	0	0	0	0.00075171	0	0	0
NONOXIPENT-PWY: pentose phosphate pathway (non-oxidative branch)|g__Mitsuokella.s__Mitsuokella_multacida	0	0.00048382	0	0	0.00078878	0	0.00089557	0	0	0
NONOXIPENT-PWY: pentose phosphate pathway (non-oxidative branch)|g__Peptoniphilus.s__Peptoniphilus_duerdenii	0	0	0	0	0	0.0011218	0	0	0	0
NONOXIPENT-PWY: pentose phosphate pathway (non-oxidative branch)|unclassified	0	0.00400688	0	0.00210279	0	0	0	0	0.00114051	0
P105-PWY: TCA cycle IV (2-oxoglutarate decarboxylase)	0.00055431	0.00083489	0.00125147	0.00204927	0.00122944	0	0	0	0	0.0008501
P105-PWY: TCA cycle IV (2-oxoglutarate decarboxylase)|g__Escherichia.s__Escherichia_coli	0	0	0.00090186	0.00067823	0.00053488	0	0	0	0	0
P108-PWY: pyruvate fermentation to propanoate I	0	0	0.00195139	0	0	0	0	0	0	0
P122-PWY: heterolactic fermentation	0	0	0	0.00080281	0	0	0	0	0	0
P162-PWY: L-glutamate degradation V (via hydroxyglutarate)	0.00067967	0	0	0	0.00191505	0.00069989	0	0	0.00105208	0.0024441
P164-PWY: purine nucleobases degradation I (anaerobic)	0.00108536	0	0	0	0	0.00475364	0.0013976	0	0.00103358	0.00139731
P4-PWY: superpathway of L-lysine, L-threonine and L-methionine biosynthesis I	0.00200046	0.00233265	0.00150648	0.00737515	0.00331637	0	0.00282423	0	0.00481177	0.00104024
P42-PWY: incomplete reductive TCA cycle	0.00110883	0	0.00047817	0.00112424	0.00060761	0	0	0	0	0.00138453
P441-PWY: superpathway of N-acetylneuraminate degradation	0.00257025	0.00072089	0.00163816	0.00207615	0.00211606	0.00167549	0	0	0.00152054	0.00421351
P461-PWY: hexitol fermentation to lactate, formate, ethanol and acetate	0.00046629	0.00124974	0.00166875	0.00081244	0.00136823	0	0.00164457	0	0	0.00111662
P461-PWY: hexitol fermentation to lactate, formate, ethanol and acetate|g__Escherichia.s__Escherichia_coli	0.00038902	0.00057091	0.00093029	0	0.0006391	0	0	0	0	0.00064284
P461-PWY: hexitol fermentation to lactate, formate, ethanol and acetate|g__Megamonas.s__Megamonas_hypermegale	0	0	0	0	0	0	0.00078066	0	0	0
P461-PWY: hexitol fermentation to lactate, formate, ethanol and acetate|g__Megamonas.s__Megamonas_rupellensis	0	0	0	0	0	0	0.00065603	0	0	0
PANTO-PWY: phosphopantothenate biosynthesis I|g__Actinomyces.s__Actinomyces_turicensis	0	0	0	0	0	0.00066473	0	0	0.00075783	0
PANTO-PWY: phosphopantothenate biosynthesis I|g__Akkermansia.s__Akkermansia_muciniphila	0	0	0.00502441	0	0	0	0	0	0	0

PANTO-PWY: phosphopantothenate biosynthesis Ilg__Bacteroides.s__Bacteroides_fragilis	0.00738504	0	0.00129214	0	0	0	0	0	0	0.00502106
PANTO-PWY: phosphopantothenate biosynthesis Ilg__Bacteroides.s__Bacteroides_vulgatus	0	0	0.00093062	0	0.00138023	0.00059759	0	0	0	0
PANTO-PWY: phosphopantothenate biosynthesis Ilg__Blautia.s__Ruminococcus_gnavus	0	0	0.000609	0	0	0	0	0	0	0
PANTO-PWY: phosphopantothenate biosynthesis Ilg__Blautia.s__Ruminococcus_torques	0	0	0	0	0	0	0.00064895	0	0	0
PANTO-PWY: phosphopantothenate biosynthesis Ilg__Desulfovibrio.s__Desulfovibrio_piger	0	0	0.00027007	0	0	0	0	0	0	0
PANTO-PWY: phosphopantothenate biosynthesis Ilg__Dorea.s__Dorea_longicatena	0.00025344	0	0	0	0	0	0	0	0	0
PANTO-PWY: phosphopantothenate biosynthesis Ilg__Eggerthella.s__Eggerthella_lenta	0	0	0.00051613	0	0	0	0	0	0	0
PANTO-PWY: phosphopantothenate biosynthesis Ilg__Escherichia.s__Escherichia_coli	0.00020198	0	0.00033588	0.00052269	0	0	0	0	0	0
PANTO-PWY: phosphopantothenate biosynthesis Ilg__Eubacterium.s__Eubacterium_rectale	0.00026041	0	0	0.00094496	0.00099613	0	0	0	0.00131445	0.00042151
PANTO-PWY: phosphopantothenate biosynthesis Ilg__Lachnospiraceae_noname.s__Lachnospiraceae_bacterium_2_1_58FAA	0	0	0.00088362	0	0	0	0	0	0	0
PANTO-PWY: phosphopantothenate biosynthesis Ilg__Megamonas.s__Megamonas_rupellensis	0	0	0	0	0	0	0.00116195	0	0	0
PANTO-PWY: phosphopantothenate biosynthesis Ilg__Megasphaera.s__Megasphaera_elsdenii	0	0	0	0.00110358	0.00036055	0	0	0	0	0
PANTO-PWY: phosphopantothenate biosynthesis Ilg__Phascolarctobacterium.s__Phascolarctobacterium_succinatutens	0	0.00043156	0	0	0	0	0	0	0	0
PANTO-PWY: phosphopantothenate biosynthesis Ilunclassified	0	0.00767098	0	0.00286482	0	0	0.00265933	0	0	0
PANTOSYN-PWY: pantothenate and coenzyme A biosynthesis Ilg__Akkermansia.s__Akkermansia_muciniphila	0	0	0.00473084	0	0	0	0	0	0	0
PANTOSYN-PWY: pantothenate and coenzyme A biosynthesis Ilg__Escherichia.s__Escherichia_coli	0	0	0.0004323	0	0	0	0	0	0	0
PEPTIDOGLYCANSYN-PWY: peptidoglycan biosynthesis I (meso-diaminopimelate containing)lg__Actinomyces.s__Actinomyces_turicensis	0	0	0	0	0	0.00123961	0	0	0.00043823	0
PEPTIDOGLYCANSYN-PWY: peptidoglycan biosynthesis I (meso-diaminopimelate containing)lg__Bacteroides.s__Bacteroides_fragilis	0.00835622	0	0.00099367	0	0	0	0	0	0.00035804	0.00418328
PEPTIDOGLYCANSYN-PWY: peptidoglycan biosynthesis I (meso-diaminopimelate containing)lg__Bacteroides.s__Bacteroides_vulgatus	0	0	0.00079061	0	0.00069595	0.00052618	0	0	0	0
PEPTIDOGLYCANSYN-PWY: peptidoglycan biosynthesis I (meso-diaminopimelate containing)lg__Bifidobacterium.s__Bifidobacterium_adolescentis	0.00215392	0	0	0.00202407	0.00641785	0	0.00095298	0	0.00227002	0
PEPTIDOGLYCANSYN-PWY: peptidoglycan biosynthesis I (meso-diaminopimelate containing)lg__Blautia.s__Ruminococcus_gnavus	0.003927	0	0.0015116	0	0	0	0	0	0	0
PEPTIDOGLYCANSYN-PWY: peptidoglycan biosynthesis I (meso-diaminopimelate containing)lg__Blautia.s__Ruminococcus_torques	0	0	0	0	0	0	0.00054469	0	0	0
PEPTIDOGLYCANSYN-PWY: peptidoglycan biosynthesis I (meso-diaminopimelate containing)lg__Collinsella.s__Collinsella_aerofaciens	0	0	0	0.00052464	0	0	0	0	0.00159924	0
PEPTIDOGLYCANSYN-PWY: peptidoglycan biosynthesis I (meso-diaminopimelate containing)lg__Escherichia.s__Escherichia_coli	0	0	0.00045548	0	0	0	0	0	0	0
PEPTIDOGLYCANSYN-PWY: peptidoglycan biosynthesis I (meso-diaminopimelate containing)lg__Faecalibacterium.s__Faecalibacterium_prausnitzii	0	0	0	0	0	0	0.00040233	0	0.00204134	0.00132649
PEPTIDOGLYCANSYN-PWY: peptidoglycan biosynthesis I (meso-diaminopimelate containing)lg__Fusobacterium.s__Fusobacterium_gonidiaformans	0	0	0	0	0	0	0	0	0	0.00041077
PEPTIDOGLYCANSYN-PWY: peptidoglycan biosynthesis I (meso-diaminopimelate containing)lg__Lachnospiraceae_noname.s__Lachnospiraceae_bacterium_2_1_58FAA	0	0	0.00114574	0	0	0	0	0	0	0
PEPTIDOGLYCANSYN-PWY: peptidoglycan biosynthesis I (meso-diaminopimelate	0.0005632	0	0	0	0.00051224	0	0	0	0	0

containing)|g__Lactobacillus.s__Lactobacillus_ruminis										
PEPTIDOGLYCANSYN-PWY: peptidoglycan biosynthesis I (meso-diaminopimelate containing)|g__Megasphaera.s__Megasphaera_elsdenii	0	0	0	0	0.00049308	0	0	0	0	0
PEPTIDOGLYCANSYN-PWY: peptidoglycan biosynthesis I (meso-diaminopimelate containing)|g__Mitsuokella.s__Mitsuokella_multacida	0	0	0	0.00054605	0.00071485	0	0.00094336	0	0	0
PEPTIDOGLYCANSYN-PWY: peptidoglycan biosynthesis I (meso-diaminopimelate containing)|g__Peptoniphilus.s__Peptoniphilus_duerdenii	0	0	0	0	0	0.00064633	0	0	0	0
PEPTIDOGLYCANSYN-PWY: peptidoglycan biosynthesis I (meso-diaminopimelate containing)|g__Peptoniphilus.s__Peptoniphilus_lacrimalis	0	0	0	0	0	0	0	0	0	0.00022725
PEPTIDOGLYCANSYN-PWY: peptidoglycan biosynthesis I (meso-diaminopimelate containing)|g__Porphyromonas.s__Porphyromonas_asaccharolytica	0	0	0	0	0	0.00078865	0	0	0.00051962	0
PEPTIDOGLYCANSYN-PWY: peptidoglycan biosynthesis I (meso-diaminopimelate containing)|unclassified	0	0.00673556	0	0.00283636	0	0	0.00248362	0	0.00185135	0
POLYAMINSYN3-PWY: superpathway of polyamine biosynthesis II	0.00028521	0.0004805	0.00049533	0	0	0	0	0	0	0.00025886
POLYAMSYN-PWY: superpathway of polyamine biosynthesis I	0.00038725	0.00077359	0.00074646	0	0.00112979	0	0.00451063	0	0.00047828	0.0014146
POLYAMSYN-PWY: superpathway of polyamine biosynthesis I|g__Escherichia.s__Escherichia_coli	0	0	0.00035325	0	0	0	0	0	0	0
POLYAMSYN-PWY: superpathway of polyamine biosynthesis I|g__Megamonas.s__Megamonas_hypermegale	0	0	0	0	0	0	0.00040459	0	0	0
POLYAMSYN-PWY: superpathway of polyamine biosynthesis I|g__Megamonas.s__Megamonas_rupellensis	0	0	0	0	0	0	0.0005747	0	0	0
POLYISOPRENSYN-PWY: polyisoprenoid biosynthesis (E. coli)	0	0.00049692	0.00048684	0.00056567	0.00086666	0	0	0.0022152	0	0.00048573
POLYISOPRENSYN-PWY: polyisoprenoid biosynthesis (E. coli)|g__Escherichia.s__Escherichia_coli	0	0	0.00044125	0.00045921	0	0	0	0	0	0.00032858
PPGPPMET-PWY: ppGpp biosynthesis	0.00043658	0.00036504	0	0	0.00126844	0	0	0	0	0
PRPP-PWY: superpathway of histidine, purine, and pyrimidine biosynthesis	0	0	0.00076252	0	0	0	0	0	0	0
PWY-1042: glycolysis IV (plant cytosol)|g__Akkermansia.s__Akkermansia_muciniphila	0	0	0.00602186	0	0	0	0	0	0	0
PWY-1042: glycolysis IV (plant cytosol)|g__Blautia.s__Ruminococcus_torques	0	0	0	0	0	0	0.00069293	0	0	0
PWY-1042: glycolysis IV (plant cytosol)|g__Escherichia.s__Escherichia_coli	0	0	0.00115987	0.00053762	0.0012274	0	0	0	0	0.00037704
PWY-1042: glycolysis IV (plant cytosol)|g__Faecalibacterium.s__Faecalibacterium_prausnitzii	0	0.00151697	0	0	0	0	0.00159303	0	0.00234924	0.00191565
PWY-1042: glycolysis IV (plant cytosol)|g__Fusobacterium.s__Fusobacterium_gonidiaformans	0.00018205	0	0	0	0	0	0	0	0	0.00068793
PWY-1042: glycolysis IV (plant cytosol)|g__Peptoniphilus.s__Peptoniphilus_lacrimalis	0	0	0	0	0	0.00046399	0	0	0	0
PWY-1042: glycolysis IV (plant cytosol)|unclassified	0	0	0	0	0	0.00136129	0.00329472	0	0.00257054	0
PWY-1269: CMP-3-deoxy-D-manno-octulosonate biosynthesis I|g__Akkermansia.s__Akkermansia_muciniphila	0	0	0.00517292	0	0	0	0	0	0	0
PWY-1269: CMP-3-deoxy-D-manno-octulosonate biosynthesis I|g__Escherichia.s__Escherichia_coli	0	0	0.0009113	0	0	0	0	0	0	0
PWY-1269: CMP-3-deoxy-D-manno-octulosonate biosynthesis I|g__Megamonas.s__Megamonas_rupellensis	0	0	0	0	0	0	0.00080345	0	0	0
PWY-1269: CMP-3-deoxy-D-manno-octulosonate biosynthesis I|unclassified	0	0.0045253	0	0.00268023	0	0	0.00286357	0	0	0
PWY-2723: trehalose degradation V	0.00032073	0	0.00051265	0	0.00094591	0	0	0	0	0.00090753
PWY-2723: trehalose degradation V|g__Escherichia.s__Escherichia_coli	0	0	0.00025306	0	0	0	0	0	0	0

PWY-2941: L-lysine biosynthesis II|g__Catenibacterium.s__Catenibacterium_mitsuokai	0	0	0	0	0.00060352	0	0	0	0	0
PWY-2941: L-lysine biosynthesis II|g__Lactobacillus.s__Lactobacillus_ruminis	0.00081487	0	0	0	0.00063558	0	0	0	0	0
PWY-2941: L-lysine biosynthesis II|unclassified	0	0	0	0	0	0.0103553	0	0	0	0
PWY-2942: L-lysine biosynthesis III|g__Actinomyces.s__Actinomyces_turicensis	0	0	0	0	0	0.00133863	0	0	0.0005955	0
PWY-2942: L-lysine biosynthesis III|g__Akkermansia.s__Akkermansia_muciniphila	0	0	0.00504931	0	0	0	0	0	0	0
PWY-2942: L-lysine biosynthesis III|g__Bacteroides.s__Bacteroides_fragilis	0.00687204	0	0.0011163	0	0	0	0	0	0.00025889	0.0040964
PWY-2942: L-lysine biosynthesis III|g__Bacteroides.s__Bacteroides_vulgatus	0	0	0.00083407	0	0	0.00035787	0	0	0	0
PWY-2942: L-lysine biosynthesis III|g__Blautia.s__Ruminococcus_gnavus	0.00331157	0	0.00166555	0	0	0	0	0	0	0
PWY-2942: L-lysine biosynthesis III|g__Blautia.s__Ruminococcus_torques	0	0	0	0	0	0	0.0011023	0	0	0
PWY-2942: L-lysine biosynthesis III|g__Catenibacterium.s__Catenibacterium_mitsuokai	0	0.00076926	0	0	0.00055579	0	0.00029025	0	0.00025189	0
PWY-2942: L-lysine biosynthesis III|g__Clostridium.s__Clostridium_hathewayi	0	0	0	0	0	0	0	0	0	0.00107497
PWY-2942: L-lysine biosynthesis III|g__Erysipelotrichaceae_noname.s__Erysipelotrichaceae_bacterium_6_1_45	0	0	0.00020739	0	0	0	0	0	0	0
PWY-2942: L-lysine biosynthesis III|g__Faecalibacterium.s__Faecalibacterium_prausnitzii	0	0	0	0	0	0	0.0006851	0	0.00057372	0.00085855
PWY-2942: L-lysine biosynthesis III|g__Megamonas.s__Megamonas_rupellensis	0	0	0	0	0	0	0.00051961	0	0	0
PWY-2942: L-lysine biosynthesis III|g__Mitsuokella.s__Mitsuokella_multacida	0	0	0	0	0.00086509	0	0.00078802	0	0	0
PWY-2942: L-lysine biosynthesis III|g__Prevotella.s__Prevotella_copri	0	0.00717084	0	0.00374684	0	0	0.00799333	0	0.00324871	0.00094189
PWY-2942: L-lysine biosynthesis III|g__Prevotella.s__Prevotella_stercorea	0	0	0	0	0	0	0	0	0.00046116	0
PWY-2942: L-lysine biosynthesis III|g__Prevotella.s__Prevotella_timonensis	0	0	0	0	0	0.00234421	0	0	0	0
PWY-2942: L-lysine biosynthesis III|unclassified	0	0.00484423	0	0	0	0.0136038	0.00374821	0	0.00139291	0
PWY-3001: superpathway of L-isoleucine biosynthesis I|g__Escherichia.s__Escherichia_coli	0	0	0.00025236	0	0.00044971	0	0	0	0	0
PWY-3001: superpathway of L-isoleucine biosynthesis I|g__Faecalibacterium.s__Faecalibacterium_prausnitzii	0.00017881	0.00159123	0	0.00064824	0.00035375	0	0.00085224	0	0.00221281	0.00154216
PWY-3001: superpathway of L-isoleucine biosynthesis I|g__Mitsuokella.s__Mitsuokella_multacida	0	0	0	0	0	0	0.00092789	0	0	0
PWY-3781: aerobic respiration I (cytochrome c)	0	0	0	0.00041234	0	0	0.00228558	0	0	0
PWY-3841: folate transformations II|g__Akkermansia.s__Akkermansia_muciniphila	0	0	0.00525817	0	0	0	0	0	0	0
PWY-3841: folate transformations II|g__Bacteroides.s__Bacteroides_fragilis	0.00715557	0	0.00111591	0	0	0	0	0	0	0.00331296
PWY-3841: folate transformations II|g__Bacteroides.s__Bacteroides_vulgatus	0	0	0.00035117	0	0.00060289	0	0	0	0	0
PWY-4041: γ-glutamyl cycle	0.00029861	0	0.00131841	0.00108452	0.00067551	0	0	0	0	0.00022961
PWY-4041: γ-glutamyl cycle|g__Escherichia.s__Escherichia_coli	0.000217	0	0.00121983	0.00051613	0.00059391	0	0	0	0	0.00022961
PWY-4242: pantothenate and coenzyme A biosynthesis III|g__Akkermansia.s__Akkermansia_muciniphila	0	0	0.00409003	0	0	0	0	0	0	0
PWY-4242: pantothenate and coenzyme A biosynthesis III|g__Escherichia.s__Escherichia_coli	0	0	0.00052254	0	0	0	0	0	0	0
PWY-4242: pantothenate and coenzyme A biosynthesis III|g__Faecalibacterium.s__Faecalibacterium_prausnitzii	0	0.00051801	0	0	0.00031195	0	0	0	0	0.00065783
PWY-4242: pantothenate and coenzyme A biosynthesis III|g__Fusobacterium.s__Fusobacterium_gonidiaformans	0	0	0	0	0	0	0	0	0	0.00041621
PWY-4702: phytate degradation I	0.00022882	0.0002158	0.00763077	0.00029381	0.00035661	0	0	0	0	0.00034862

PWY-4702: phytate degradation I|g__Akkermansia.s__Akkermansia_muciniphila	0	0	0.0068 6735	0	0	0	0	0	0	0
PWY-4702: phytate degradation I|g__Escherichia.s__Escherichia_coli	0.0002288 2	0	0.0007 6053	0.0002 9381	0	0	0	0	0	0.0003 4862
PWY-4981: L-proline biosynthesis II (from arginine)|g__Actinomyces.s__Actinomyces_turicensis	0.0001582 5	0	0	0	0	0.0008 186	0	0	0.0010 3526	0
PWY-4981: L-proline biosynthesis II (from arginine)|g__Anaerococcus.s__Anaerococcus_vaginalis	0	0	0	0	0	0	0	0	0	0.0002 4906
PWY-4981: L-proline biosynthesis II (from arginine)|g__Collinsella.s__Collinsella_aerofaciens	0	0.0009 5822	0	0	0.0004 025	0	0.0009 7648	0	0.0016 9819	0.0011 6984
PWY-4981: L-proline biosynthesis II (from arginine)|g__Eggerthella.s__Eggerthella_lenta	0	0	0.0003 0566	0	0	0	0	0	0	0
PWY-4981: L-proline biosynthesis II (from arginine)|g__Peptoniphilus.s__Peptoniphilus_duerdenii	0.0002120 7	0	0	0	0	0.0005 8322	0	0	0	0
PWY-5005: biotin biosynthesis II	0	0.0008 3723	0	0	0.0016 3663	0	0.0013 7957	0	0	0
PWY-5022: 4-aminobutanoate degradation V	0.0008511 4	0.0006 6137	0.0012 2126	0	0.0012 9896	0	0	0	0.0003 8486	0.0005 8905
PWY-5083: NAD/NADH phosphorylation and dephosphorylation	0.0003186 8	0	0.0015 4851	0.0010 6991	0.0008 8655	0	0.0002 8532	0	0	0
PWY-5083: NAD/NADH phosphorylation and dephosphorylation|g__Escherichia.s__Escherichia_coli	0	0	0.0008 6484	0	0.0004 1527	0	0	0	0	0
PWY-5097: L-lysine biosynthesis VI|g__Actinomyces.s__Actinomyces_turicensis	0	0	0	0	0	0.0014 1893	0	0	0.0006 3704	0
PWY-5097: L-lysine biosynthesis VI|g__Akkermansia.s__Akkermansia_muciniphila	0	0	0.0041 6693	0	0	0	0	0	0	0
PWY-5097: L-lysine biosynthesis VI|g__Bacteroides.s__Bacteroides_fragilis	0.0063065 7	0	0.0010 1094	0	0	0	0	0	0.0002 3395	0.0036 405
PWY-5097: L-lysine biosynthesis VI|g__Bacteroides.s__Bacteroides_vulgatus	0	0	0.0008 2819	0	0	0.0003 9678	0	0	0	0
PWY-5097: L-lysine biosynthesis VI|g__Blautia.s__Ruminococcus_gnavus	0.0038353 5	0	0.0017 5818	0	0	0	0	0	0	0
PWY-5097: L-lysine biosynthesis VI|g__Blautia.s__Ruminococcus_torques	0	0	0	0	0	0	0.0006 2933	0	0	0
PWY-5097: L-lysine biosynthesis VI|g__Catenibacterium.s__Catenibacterium_mitsuokai	0	0	0	0	0.0005 6521	0	0.0002 9151	0	0.0002 4356	0
PWY-5097: L-lysine biosynthesis VI|g__Clostridium.s__Clostridium_hathewayi	0	0	0	0	0	0	0	0	0	0.0007 4412
PWY-5097: L-lysine biosynthesis VI|g__Erysipelotrichaceae_noname.s__Erysipelotrichaceae_bacterium_6_1_45	0	0	0.0002 1068	0	0	0	0	0	0	0
PWY-5097: L-lysine biosynthesis VI|g__Eubacterium.s__Eubacterium_rectale	0.0002505 5	0	0	0	0.0003 4337	0	0	0	0.0012 8209	0.0003 7764
PWY-5097: L-lysine biosynthesis VI|g__Faecalibacterium.s__Faecalibacterium_prausnitzii	0	0.0006 7113	0	0	0	0	0	0	0.0016 1483	0.0008 4879
PWY-5097: L-lysine biosynthesis VI|g__Lachnospiraceae_noname.s__Lachnospiraceae_bacterium_2_1_58FAA	0	0	0.0013 4178	0	0	0	0	0	0	0
PWY-5097: L-lysine biosynthesis VI|g__Megamonas.s__Megamonas_rupellensis	0	0	0	0	0	0	0.0007 5755	0	0	0
PWY-5097: L-lysine biosynthesis VI|g__Mitsuokella.s__Mitsuokella_multacida	0	0	0	0	0.0008 8334	0	0.0008 3765	0	0	0
PWY-5097: L-lysine biosynthesis VI|g__Prevotella.s__Prevotella_copri	0	0.0073 9393	0	0.0034 5944	0	0	0.0081 0419	0	0.0031 5883	0.0010 3719
PWY-5097: L-lysine biosynthesis VI|g__Prevotella.s__Prevotella_stercorea	0	0	0	0	0	0	0	0	0.0005 2616	0
PWY-5097: L-lysine biosynthesis VI|unclassified	0	0.0045 2013	0	0	0	0.0143 11	0.0037 2561	0	0.0014 5768	0
PWY-5100: pyruvate fermentation to acetate and lactate II|g__Anaerococcus.s__Anaerococcus_lactolyticus	0	0	0	0	0	0.0005 7137	0	0	0	0
PWY-5100: pyruvate fermentation to acetate and lactate II|g__Anaerococcus.s__Anaerococcus_vaginalis	0	0	0	0	0	0	0	0	0	0.0002 0271
PWY-5100: pyruvate fermentation to acetate and lactate II|g__Eggerthella.s__Eggerthella_lenta	0	0	0.0001 6581	0	0	0	0	0	0	0
PWY-5100: pyruvate fermentation to acetate and lactate II|g__Erysipelotrichaceae_noname.s__Eubacterium_biforme	0	0.0005 0469	0	0.0004 0557	0.0002 7155	0	0.0005 5826	0	0.0001 6934	0.0002 4915

PWY-5100: pyruvate fermentation to acetate and lactate II|g__Fusobacterium.s__Fusobacterium_gonidiaformans	0.00015749	0	0	0	0	0	0	0	0	0.00065658
PWY-5100: pyruvate fermentation to acetate and lactate II|g__Peptoniphilus.s__Peptoniphilus_duerdenii	0	0	0	0	0	0.00038427	0	0	0	0
PWY-5100: pyruvate fermentation to acetate and lactate II|unclassified	0	0	0	0	0	0	0	0	0.00071238	0
PWY-5101: L-isoleucine biosynthesis II	0.00068383	0	0.00058239	0	0	0	0	0	0.00025451	0
PWY-5101: L-isoleucine biosynthesis II|g__Methanobrevibacter.s__Methanobrevibacter_smithii	0	0	0.00017708	0	0	0	0	0	0	0
PWY-5103: L-isoleucine biosynthesis III|g__Akkermansia.s__Akkermansia_muciniphila	0	0	0.00627132	0	0	0	0	0	0	0
PWY-5103: L-isoleucine biosynthesis III|g__Bifidobacterium.s__Bifidobacterium_longum	0.00020621	0	0	0	0	0	0	0	0	0
PWY-5103: L-isoleucine biosynthesis III|g__Desulfovibrio.s__Desulfovibrio_piger	0	0	0.00022722	0	0	0	0	0	0	0
PWY-5103: L-isoleucine biosynthesis III|g__Escherichia.s__Escherichia_coli	0	0	0	0	0.00093014	0	0	0	0	0
PWY-5103: L-isoleucine biosynthesis III|g__Faecalibacterium.s__Faecalibacterium_prausnitzii	0.00018047	0.00279315	0	0.00063301	0.00064364	0	0.00084393	0	0.00324497	0.00205951
PWY-5103: L-isoleucine biosynthesis III|g__Megamonas.s__Megamonas_hypermegale	0	0	0	0	0	0	0.00023457	0	0	0
PWY-5103: L-isoleucine biosynthesis III|g__Megamonas.s__Megamonas_rupellensis	0	0	0	0	0	0	0.00033072	0	0	0
PWY-5103: L-isoleucine biosynthesis III|g__Mitsuokella.s__Mitsuokella_multacida	0	0	0	0	0	0	0.00096419	0	0	0
PWY-5104: L-isoleucine biosynthesis IV	0.00233312	0	0.00039539	0	0	0	0.0005121	0	0	0.00153225
PWY-5104: L-isoleucine biosynthesis IV|g__Methanobrevibacter.s__Methanobrevibacter_smithii	0	0	0.0001652	0	0	0	0	0	0	0
PWY-5136: fatty acid β-oxidation II (peroxisome)	0	0.00036336	0.00012677	0.00033165	0	0	0	0	0.00027292	0.0003563
PWY-5136: fatty acid β-oxidation II (peroxisome)|g__Escherichia.s__Escherichia_coli	0	0	0.0001257	0.00025289	0	0	0	0	0	0
PWY-5138: unsaturated, even numbered fatty acid β-oxidation	0	0	8.05E-05	0.0001701	0	0	0	0	0	0
PWY-5138: unsaturated, even numbered fatty acid β-oxidation|g__Escherichia.s__Escherichia_coli	0	0	8.05E-05	0.0001701	0	0	0	0	0	0
PWY-5154: L-arginine biosynthesis III (via N-acetyl-L-citrulline)	0.00095187	0	0.00206106	0.00079144	0.00095489	0	0.00164231	0	0	0
PWY-5173: superpathway of acetyl-CoA biosynthesis	0	0.00039191	0.00047222	0	0.00100468	0	0	0	0	0.0002858
PWY-5173: superpathway of acetyl-CoA biosynthesis|g__Escherichia.s__Escherichia_coli	0	0	0.00038652	0	0.00047704	0	0	0	0	0.00024656
PWY-5177: glutaryl-CoA degradation	0.00048051	0.00192168	0.00015537	0.00107263	0.00095968	0	0.00087775	0	0.00227699	0.00124332
PWY-5177: glutaryl-CoA degradation|g__Faecalibacterium.s__Faecalibacterium_prausnitzii	0	0.00067348	0	0.00065913	0.0005552	0	0.00080533	0	0.0018984	0.00093799
PWY-5188: tetrapyrrole biosynthesis I (from glutamate)|g__Actinomyces.s__Actinomyces_turicensis	0.00018923	0	0	0	0	0.00105412	0	0	0.00071692	0
PWY-5188: tetrapyrrole biosynthesis I (from glutamate)|g__Akkermansia.s__Akkermansia_muciniphila	0	0	0.00398303	0	0	0	0	0	0	0
PWY-5188: tetrapyrrole biosynthesis I (from glutamate)|g__Blautia.s__Ruminococcus_obeum	0	0	0	0	0	0	0	0	0.00014776	0
PWY-5188: tetrapyrrole biosynthesis I (from glutamate)|g__Escherichia.s__Escherichia_coli	0.00015288	0	0.00056154	0	0	0	0	0	0	0.00029473
PWY-5188: tetrapyrrole biosynthesis I (from glutamate)|g__Fusobacterium.s__Fusobacterium_nucleatum	0	0	0	0	0	0.00027533	0	0	0	0
PWY-5188: tetrapyrrole biosynthesis I (from glutamate)|g__Megamonas.s__Megamonas_rupellensis	0	0	0	0	0	0	0.00034814	0	0	0
PWY-5188: tetrapyrrole biosynthesis I (from glutamate)|g__Megasphaera.s__Megasphaera_elsdenii	0	0	0	0	0.00040346	0	0	0	0	0
PWY-5188: tetrapyrrole biosynthesis I (from glutamate)|g__Mitsuokella.s__Mitsuokella_multacida	0	0.00034712	0	0	0.00047857	0	0.00106706	0	0	0

PWY-5188: tetrapyrrole biosynthesis I (from glutamate)|g__Ruminococcus.s__Ruminococcus_bromii	0	0	0	0	0	0	0	0	0	0.00028148
PWY-5189: tetrapyrrole biosynthesis II (from glycine)|g__Escherichia.s__Escherichia_coli	0	0	0.00055881	0	0	0	0	0	0	0
PWY-5304: superpathway of sulfur oxidation (Acidianus ambivalens)	0	0	0	0.00022137	0	0	0	0	0	0
PWY-5304: superpathway of sulfur oxidation (Acidianus ambivalens)|g__Ruminococcus.s__Ruminococcus_bromii	0	0	0	0.00022137	0	0	0	0	0	0
PWY-5345: superpathway of L-methionine biosynthesis (by sulfhydrylation)	0.00063524	0	0.00461591	0.00258525	0.00132286	0	0	0	0.00119082	0.0025655
PWY-5347: superpathway of L-methionine biosynthesis (transsulfuration)	0.00575199	0.00231801	0.0042884	0.00789722	0.0068418	0	0.00510763	0	0.00700243	0.0035608
PWY-5347: superpathway of L-methionine biosynthesis (transsulfuration)|g__Escherichia.s__Escherichia_coli	0	0	0.00030542	0	0.00034833	0	0	0	0	0
PWY-5367: petroselinate biosynthesis	0	0	0	0.00196324	0.00191014	0	0	0	0.00211236	0.00177444
PWY-5367: petroselinate biosynthesis|g__Eubacterium.s__Eubacterium_rectale	0	0	0	0.00113886	0.00054254	0	0	0	0.00132569	0.00036398
PWY-5384: sucrose degradation IV (sucrose phosphorylase)	0.00035261	0	0.00038874	0.0004113	0.00109551	0	0	0	0	0.00119425
PWY-5384: sucrose degradation IV (sucrose phosphorylase)|g__Escherichia.s__Escherichia_coli	0	0	0.0002828	0	0	0	0	0	0	0.0001573
PWY-5505: L-glutamate and L-glutamine biosynthesis	0.00030213	0	0.00100646	0	0	0	0	0	0	0.00121133
PWY-561: superpathway of glyoxylate cycle and fatty acid degradation	0	0.00051823	0.00026754	0.00062966	0	0	0	0	0	0.00052698
PWY-561: superpathway of glyoxylate cycle and fatty acid degradation|g__Escherichia.s__Escherichia_coli	0	0	0.00024541	0.0003792	0	0	0	0	0	0
PWY-5656: mannosylglycerate biosynthesis I	0	0	0.00028914	0	0	0	0	0	0	0
PWY-5659: GDP-mannose biosynthesis|g__Escherichia.s__Escherichia_coli	0	0	0.00030252	0	0.00062281	0	0	0	0	0.00015645
PWY-5659: GDP-mannose biosynthesis|g__Eubacterium.s__Eubacterium_siraeum	0	0.00020326	0	0	0	0	0	0	0	0
PWY-5659: GDP-mannose biosynthesis|g__Faecalibacterium.s__Faecalibacterium_prausnitzii	0	0	0	0	0	0	0.00065067	0	0.00074171	0.00137848
PWY-5667: CDP-diacylglycerol biosynthesis I|g__Anaerococcus.s__Anaerococcus_vaginalis	0	0.0003813	0	0	0	0	0	0	0	0
PWY-5667: CDP-diacylglycerol biosynthesis I|g__Bacteroides.s__Bacteroides_fragilis	0	0	0.00033904	0	0	0	0	0	0	0.00388397
PWY-5667: CDP-diacylglycerol biosynthesis I|g__Blautia.s__Ruminococcus_gnavus	0.00400588	0	0.00207134	0	0	0	0	0	0	0
PWY-5667: CDP-diacylglycerol biosynthesis I|g__Blautia.s__Ruminococcus_torques	0	0	0	0	0	0	0.0006713	0	0	0
PWY-5667: CDP-diacylglycerol biosynthesis I|g__Clostridium.s__Clostridium_nexile	0	0	0	0	0	0	0	0	0	0.00036513
PWY-5667: CDP-diacylglycerol biosynthesis I|g__Dorea.s__Dorea_formicigenerans	0	0	0	0.00043313	0	0	0	0	0.00018517	0
PWY-5667: CDP-diacylglycerol biosynthesis I|g__Dorea.s__Dorea_longicatena	0	0	0	0	0.00034403	0	0	0	0	0
PWY-5667: CDP-diacylglycerol biosynthesis I|g__Escherichia.s__Escherichia_coli	0	0	0.00035616	0	0.00035519	0	0	0	0	0
PWY-5667: CDP-diacylglycerol biosynthesis I|g__Eubacterium.s__Eubacterium_rectale	0.00026523	0	0	0.00064232	0	0	0	0	0.0005902	0.00032202
PWY-5667: CDP-diacylglycerol biosynthesis I|g__Faecalibacterium.s__Faecalibacterium_prausnitzii	0.00026746	0.00065111	0	0.00090178	0.00037193	0	0.00044973	0	0.00187009	0.00130843
PWY-5667: CDP-diacylglycerol biosynthesis I|g__Megamonas.s__Megamonas_hypermegale	0	0	0	0	0	0	0.00048918	0	0	0
PWY-5667: CDP-diacylglycerol biosynthesis I|g__Mitsuokella.s__Mitsuokella_multacida	0	0	0	0	0	0	0.00174695	0	0	0
PWY-5667: CDP-diacylglycerol biosynthesis I|g__Peptoniphilus.s__Peptoniphilus_lacrimalis	0	0	0	0	0	0.00042239	0	0	0	0
PWY-5667: CDP-diacylglycerol biosynthesis I|g__Porphyromonas.s__Porphyromonas_asaccharolytica	0	0	0	0	0	0.00035547	0	0	0	0
PWY-5667: CDP-diacylglycerol biosynthesis I|g__Prevotella.s__Prevotella_stercorea	0	0	0	0	0	0	0	0	0.00052378	0.00068208
PWY-5667: CDP-diacylglycerol biosynthesis I|g__Roseburia.s__Roseburia_inulinivorans	0	0	0	0	0.00039173	0	0	0	0	0

PWY-5686: UMP biosynthesis|g__Actinomyces.s__Actinomyces_turicensis	0.00018768	0	0	0	0	0.00130175	0	0	0.00080602	0
PWY-5686: UMP biosynthesis|g__Akkermansia.s__Akkermansia_muciniphila	0	0	0.00602971	0	0	0	0	0	0	0
PWY-5686: UMP biosynthesis|g__Bacteroides.s__Bacteroides_fragilis	0.00538875	0	0.00052711	0	0	0	0	0	0.00025341	0.00374143
PWY-5686: UMP biosynthesis|g__Bifidobacterium.s__Bifidobacterium_catenulatum	0	0	0	0	0	0	0	0	0.00032385	0
PWY-5686: UMP biosynthesis|g__Blautia.s__Ruminococcus_gnavus	0.00301456	0	0.00086298	0	0	0	0	0	0	0
PWY-5686: UMP biosynthesis|g__Catenibacterium.s__Catenibacterium_mitsuokai	0	0	0	0	0.00049154	0	0	0	0	0
PWY-5686: UMP biosynthesis|g__Clostridium.s__Clostridium_hathewayi	0	0	0	0	0	0	0	0	0	0.00054705
PWY-5686: UMP biosynthesis|g__Clostridium.s__Clostridium_nexile	0	0	0	0	0	0	0	0	0	0.00093006
PWY-5686: UMP biosynthesis|g__Collinsella.s__Collinsella_aerofaciens	0	0	0	0	0	0	0.00045085	0	0.00144971	0.00056934
PWY-5686: UMP biosynthesis|g__Erysipelotrichaceae_noname.s__Eubacterium_biforme	0	0	0	0	0	0	0.00036869	0	0	0
PWY-5686: UMP biosynthesis|g__Escherichia.s__Escherichia_coli	0	0	0	0	0.00054809	0	0	0	0	0
PWY-5686: UMP biosynthesis|g__Eubacterium.s__Eubacterium_rectale	0	0	0	0.00107049	0.00056995	0	0	0	0.00135516	0.00029466
PWY-5686: UMP biosynthesis|g__Faecalibacterium.s__Faecalibacterium_prausnitzii	0	0.0013455	0	0	0	0	0.00045007	0	0.00208756	0.00145402
PWY-5686: UMP biosynthesis|g__Lachnospiraceae_noname.s__Lachnospiraceae_bacterium_2_1_58FAA	0	0	0.00147419	0	0	0	0	0	0	0
PWY-5686: UMP biosynthesis|g__Megamonas.s__Megamonas_rupellensis	0	0	0	0	0	0	0.00043011	0	0	0
PWY-5686: UMP biosynthesis|g__Mitsuokella.s__Mitsuokella_multacida	0	0	0	0	0	0	0.00144998	0	0	0
PWY-5686: UMP biosynthesis|g__Peptoniphilus.s__Peptoniphilus_duerdenii	0	0	0	0	0	0.00048115	0	0	0	0
PWY-5686: UMP biosynthesis|g__Porphyromonas.s__Porphyromonas_asaccharolytica	0	0	0	0	0	0.00050144	0	0	0.000244	0
PWY-5686: UMP biosynthesis|g__Prevotella.s__Prevotella_buccalis	0	0	0	0	0	0	0	0	0.00054947	0
PWY-5686: UMP biosynthesis|g__Prevotella.s__Prevotella_copri	0.00025669	0.0105527	0	0.004461	0	0	0.00792682	0	0.00403184	0.00217922
PWY-5686: UMP biosynthesis|g__Prevotella.s__Prevotella_disiens	0	0	0	0	0	0.00035136	0	0	0	0
PWY-5686: UMP biosynthesis|g__Prevotella.s__Prevotella_stercorea	0	0	0	0	0	0	0	0	0.00033056	0
PWY-5686: UMP biosynthesis|g__Prevotella.s__Prevotella_timonensis	0	0	0	0	0	0.00265883	0	0	0	0.00029632
PWY-5686: UMP biosynthesis|g__Ruminococcus.s__Ruminococcus_bromii	0	0	0	0.0003736	0	0	0	0	0	0
PWY-5686: UMP biosynthesis|unclassified	0	0.00835421	0	0	0	0	0	0	0.0012169	0
PWY-5690: TCA cycle II (plants and fungi)|g__Escherichia.s__Escherichia_coli	0	0	0.00084602	0.00059061	0.0004088	0	0	0	0	0
PWY-5692: allantoin degradation to glyoxylate II	0	0	0	0	0.0002545	0	0	0	0	0
PWY-5695: urate biosynthesis/inosine 5'-phosphate degradation|g__Anaerococcus.s__Anaerococcus_vaginalis	0	0.00054851	0	0	0	0	0	0	0	0.00023706
PWY-5695: urate biosynthesis/inosine 5'-phosphate degradation|g__Bacteroides.s__Bacteroides_fragilis	0.00641594	0	0.0008237	0	0	0	0	0	0.00024742	0.00385261
PWY-5695: urate biosynthesis/inosine 5'-phosphate degradation|g__Bacteroides.s__Bacteroides_vulgatus	0.00013472	0	0.00057262	0	0.00084762	0.00044509	0.00021382	0	0	0
PWY-5695: urate biosynthesis/inosine 5'-phosphate degradation|g__Blautia.s__Ruminococcus_torques	0	0	0	0	0	0	0.00060476	0	0	0

PWY-5695: urate biosynthesis/inosine 5'-phosphate degradation|g__Escherichia.s__Escherichia_coli	0.00025695	0.00052141	0.00084517	0.00039401	0.00071619	0	0	0	0	0
PWY-5695: urate biosynthesis/inosine 5'-phosphate degradation|g__Faecalibacterium.s__Faecalibacterium_prausnitzii	0.00035753	0.00078501	0	0	0.00043631	0	0	0	0.00208274	0
PWY-5695: urate biosynthesis/inosine 5'-phosphate degradation|g__Megasphaera.s__Megasphaera_elsdenii	0	0	0	0.00068564	0.00055781	0	0	0	0	0
PWY-5695: urate biosynthesis/inosine 5'-phosphate degradation|g__Peptoniphilus.s__Peptoniphilus_duerdenii	0	0	0	0	0	0.00108197	0	0	0	0
PWY-5695: urate biosynthesis/inosine 5'-phosphate degradation|g__Peptoniphilus.s__Peptoniphilus_lacrimalis	0	0	0	0	0	0.00043744	0	0	0	0.00043578
PWY-5695: urate biosynthesis/inosine 5'-phosphate degradation|g__Porphyromonas.s__Porphyromonas_asaccharolytica	0.00014487	0	0	0	0	0.0007022	0	0	0.00035523	0
PWY-5695: urate biosynthesis/inosine 5'-phosphate degradation|g__Prevotella.s__Prevotella_disiens	0	0	0	0	0	0	0	0	0	0.00022256
PWY-5695: urate biosynthesis/inosine 5'-phosphate degradation|g__Prevotella.s__Prevotella_timonensis	0	0	0	0	0	0.00286139	0.00022294	0	0	0
PWY-5695: urate biosynthesis/inosine 5'-phosphate degradation|g__Roseburia.s__Roseburia_inulinivorans	0	0.00095219	0	0	0.00050442	0	0	0	0	0
PWY-5695: urate biosynthesis/inosine 5'-phosphate degradation|unclassified	0	0.00226862	0	0	0	0	0.00374865	0	0.00234314	0
PWY-5705: allantoin degradation to glyoxylate III	0	0	0.00031697	0	0.00039295	0	0	0	0	0
PWY-5705: allantoin degradation to glyoxylate III|g__Escherichia.s__Escherichia_coli	0	0	0.00031697	0	0.00039295	0	0	0	0	0
PWY-5723: Rubisco shunt	0	0	0.00071988	0.00142703	0.0017649	0	0	0	0	0.00085145
PWY-5747: 2-methylcitrate cycle II	0	0	0	0.00044968	0	0	0	0	0	0
PWY-5791: 1,4-dihydroxy-2-naphthoate biosynthesis II (plants)	0	0	0.00160091	0.00158681	0.00038867	0	0	0	0	0
PWY-5791: 1,4-dihydroxy-2-naphthoate biosynthesis II (plants)|g__Escherichia.s__Escherichia_coli	0	0	0.00078571	0	0	0	0	0	0	0
PWY-5837: 1,4-dihydroxy-2-naphthoate biosynthesis I	0	0	0.00160091	0.00158681	0.00038867	0	0	0	0	0
PWY-5837: 1,4-dihydroxy-2-naphthoate biosynthesis I|g__Escherichia.s__Escherichia_coli	0	0	0.00078571	0	0	0	0	0	0	0
PWY-5838: superpathway of menaquinol-8 biosynthesis I	0	0	0.00292829	0.00242085	0.00102825	0	0	0	0	0
PWY-5838: superpathway of menaquinol-8 biosynthesis I|g__Escherichia.s__Escherichia_coli	0	0	0.00036476	0	0	0	0	0	0	0
PWY-5840: superpathway of menaquinol-7 biosynthesis	0	0	0.00292829	0	0.00092456	0	0	0	0	0
PWY-5840: superpathway of menaquinol-7 biosynthesis|g__Escherichia.s__Escherichia_coli	0	0	0.00036476	0	0	0	0	0	0	0
PWY-5845: superpathway of menaquinol-9 biosynthesis	0	0	0.00292829	0	0	0	0	0	0	0
PWY-5845: superpathway of menaquinol-9 biosynthesis|g__Escherichia.s__Escherichia_coli	0	0	0.00036476	0	0	0	0	0	0	0
PWY-5850: superpathway of menaquinol-6 biosynthesis I	0	0	0.00292829	0	0	0	0	0	0	0
PWY-5850: superpathway of menaquinol-6 biosynthesis I|g__Escherichia.s__Escherichia_coli	0	0	0.00036476	0	0	0	0	0	0	0
PWY-5855: ubiquinol-7 biosynthesis (prokaryotic)	0	0	0.00079682	0	0.00057287	0	0	0	0	0
PWY-5855: ubiquinol-7 biosynthesis (prokaryotic)|g__Escherichia.s__Escherichia_coli	0	0	0.0004846	0	0.0004504	0	0	0	0	0
PWY-5856: ubiquinol-9 biosynthesis (prokaryotic)	0	0	0.00079682	0	0.00057287	0	0	0	0	0
PWY-5856: ubiquinol-9 biosynthesis (prokaryotic)|g__Escherichia.s__Escherichia_coli	0	0	0.0004846	0	0.0004504	0	0	0	0	0
PWY-5857: ubiquinol-10 biosynthesis (prokaryotic)	0	0	0.00079682	0	0.00057287	0	0	0	0	0
PWY-5857: ubiquinol-10 biosynthesis (prokaryotic)|g__Escherichia.s__Escherichia_coli	0	0	0.0004846	0	0.0004504	0	0	0	0	0
PWY-5860: superpathway of demethylmenaquinol-6 biosynthesis I	0	0	0.00230041	0	0	0	0	0	0	0
PWY-5860: superpathway of demethylmenaquinol-6 biosynthesis I|g__Escherichia.s__Escherichia_coli	0	0	0.00044924	0	0	0	0	0	0	0
PWY-5861: superpathway of demethylmenaquinol-8 biosynthesis	0	0	0.00230041	0.001811	0.00074927	0	0	0	0	0

PWY-5861: superpathway of demethylmenaquinol-8 biosynthesis|g__Escherichia.s__Escherichia_coli	0	0	0.00044924	0	0	0	0	0	0	0
PWY-5862: superpathway of demethylmenaquinol-9 biosynthesis	0	0	0.00230041	0	0	0	0	0	0	0
PWY-5862: superpathway of demethylmenaquinol-9 biosynthesis|g__Escherichia.s__Escherichia_coli	0	0	0.00044924	0	0	0	0	0	0	0
PWY-5863: superpathway of phylloquinol biosynthesis	0	0	0.00134901	0	0	0	0	0	0	0
PWY-5863: superpathway of phylloquinol biosynthesis|g__Escherichia.s__Escherichia_coli	0	0	0.00049182	0	0	0	0	0	0	0
PWY-5896: superpathway of menaquinol-10 biosynthesis	0	0	0.00292829	0	0	0	0	0	0	0
PWY-5896: superpathway of menaquinol-10 biosynthesis|g__Escherichia.s__Escherichia_coli	0	0	0.00036476	0	0	0	0	0	0	0
PWY-5897: superpathway of menaquinol-11 biosynthesis	0	0	0.00330451	0.00356514	0.00097525	0	0	0	0	0
PWY-5897: superpathway of menaquinol-11 biosynthesis|g__Escherichia.s__Escherichia_coli	0	0	0.00038576	0	0	0	0	0	0	0
PWY-5898: superpathway of menaquinol-12 biosynthesis	0	0	0.00330451	0.00356514	0.00097525	0	0	0	0	0
PWY-5898: superpathway of menaquinol-12 biosynthesis|g__Escherichia.s__Escherichia_coli	0	0	0.00038576	0	0	0	0	0	0	0
PWY-5899: superpathway of menaquinol-13 biosynthesis	0	0	0.00330451	0.00356514	0.00097525	0	0	0	0	0
PWY-5899: superpathway of menaquinol-13 biosynthesis|g__Escherichia.s__Escherichia_coli	0	0	0.00038576	0	0	0	0	0	0	0
PWY-5913: TCA cycle VI (obligate autotrophs)	0.00021254	0	0.00099501	0.0010525	0.00035665	0	0	0	0	0.00041065
PWY-5913: TCA cycle VI (obligate autotrophs)|g__Escherichia.s__Escherichia_coli	0	0	0.00061074	0	0.00032222	0	0	0	0	0
PWY-5920: superpathway of heme biosynthesis from glycine	0.000447	0	0	0	0	0	0	0	0	0.00046785
PWY-5971: palmitate biosynthesis II (bacteria and plants)	0	0	0	0	0.0020462	0	0	0	0	0.00078605
PWY-5973: cis-vaccenate biosynthesis	0.00842286	0.01061	0.00429459	0.00745819	0.00532268	0.00304855	0.00950974	0	0.00952749	0.00868679
PWY-5973: cis-vaccenate biosynthesis|g__Campylobacter.s__Campylobacter_hominis	0	0	0	0	0	0	0.00034468	0	0	0
PWY-5973: cis-vaccenate biosynthesis|g__Eubacterium.s__Eubacterium_rectale	0	0	0	0	0.00063662	0	0	0	0.00145846	0
PWY-5973: cis-vaccenate biosynthesis|g__Prevotella.s__Prevotella_buccalis	0	0	0	0	0	0	0	0	0.00030318	0
PWY-5973: cis-vaccenate biosynthesis|g__Prevotella.s__Prevotella_disiens	0	0.00033962	0	0	0	0	0	0	0	0.00022498
PWY-5989: stearate biosynthesis II (bacteria and plants)	0.00062966	0.0013391	0.00090149	0.00196746	0.00192513	0	0.00057213	0	0.00267821	0.00225291
PWY-5989: stearate biosynthesis II (bacteria and plants)|g__Escherichia.s__Escherichia_coli	0	0	0	0	0.00042883	0	0	0	0	0
PWY-6113: superpathway of mycolate biosynthesis	0	0	0	0	0.00199056	0	0	0	0	0.00080709
PWY-6121: 5-aminoimidazole ribonucleotide biosynthesis |g__Actinomyces.s__Actinomyces_turicensis	0	0	0	0	0	0.00181138	0	0	0.00121232	0
PWY-6121: 5-aminoimidazole ribonucleotide biosynthesis |g__Akkermansia.s__Akkermansia_muciniphila	0	0	0.0061348	0	0	0	0	0	0	0
PWY-6121: 5-aminoimidazole ribonucleotide biosynthesis |g__Bifidobacterium.s__Bifidobacterium_longum	0.00032833	0	0	0	0	0	0	0	0	0
PWY-6121: 5-aminoimidazole ribonucleotide biosynthesis |g__Blautia.s__Ruminococcus_gnavus	0.00405401	0	0.00100337	0	0	0	0	0	0	0
PWY-6121: 5-aminoimidazole ribonucleotide biosynthesis |g__Catenibacterium.s__Catenibacterium_mitsuokai	0	0	0	0	0.00068219	0	0	0	0	0
PWY-6121: 5-aminoimidazole ribonucleotide biosynthesis |g__Clostridium.s__Clostridium_hathewayi	0	0	0	0	0	0	0	0	0	0.00057415
PWY-6121: 5-aminoimidazole ribonucleotide biosynthesis |g__Clostridium.s__Clostridium_nexile	0	0	0	0	0	0	0	0	0	0.00071788
PWY-6121: 5-aminoimidazole ribonucleotide biosynthesis |g__Collinsella.s__Collinsella_aerofaciens	0	0.00048487	0	0	0	0	0	0	0.0021591	0.0009234
PWY-6121: 5-aminoimidazole ribonucleotide biosynthesis |g__Desulfovibrio.s__Desulfovibrio_piger	0	0	0.00023922	0	0	0	0	0	0	0

PWY-6121: 5-aminoimidazole ribonucleotide biosynthesis Ilg__Eggerthella.s__Eggerthella_lenta	0	0	0.00018417	0	0	0	0	0	0	0
PWY-6121: 5-aminoimidazole ribonucleotide biosynthesis Ilg__Erysipelotrichaceae_noname.s__Eubacterium_biforme	0	0.00046865	0	0.00043572	0	0	0.00032361	0	0	0.00035084
PWY-6121: 5-aminoimidazole ribonucleotide biosynthesis Ilg__Escherichia.s__Escherichia_coli	0	0	0.00020975	0	0	0	0	0	0	0
PWY-6121: 5-aminoimidazole ribonucleotide biosynthesis Ilg__Faecalibacterium.s__Faecalibacterium_prausnitzii	0	0.00074452	0	0.00046297	0.0004658	0	0.00099682	0	0.00128493	0.00064624
PWY-6121: 5-aminoimidazole ribonucleotide biosynthesis Ilg__Fusobacterium.s__Fusobacterium_gonidiaformans	0	0	0	0	0	0	0	0	0	0.00059015
PWY-6121: 5-aminoimidazole ribonucleotide biosynthesis Ilg__Fusobacterium.s__Fusobacterium_nucleatum	0	0	0	0	0	0.00021698	0	0	0	0
PWY-6121: 5-aminoimidazole ribonucleotide biosynthesis Ilg__Lachnospiraceae_noname.s__Lachnospiraceae_bacterium_2_1_58FAA	0	0	0.00142261	0	0	0	0	0	0	0
PWY-6121: 5-aminoimidazole ribonucleotide biosynthesis Ilg__Lactobacillus.s__Lactobacillus_ruminis	0.00084399	0	0	0	0.00066674	0	0	0	0	0
PWY-6121: 5-aminoimidazole ribonucleotide biosynthesis Ilg__Megamonas.s__Megamonas_rupellensis	0	0	0	0	0	0	0.00063277	0	0	0
PWY-6121: 5-aminoimidazole ribonucleotide biosynthesis Ilg__Megasphaera.s__Megasphaera_elsdenii	0	0	0	0	0.00052827	0	0	0	0	0
PWY-6121: 5-aminoimidazole ribonucleotide biosynthesis Ilg__Methanobrevibacter.s__Methanobrevibacter_smithii	0	0	0.00021575	0	0	0	0	0	0	0
PWY-6121: 5-aminoimidazole ribonucleotide biosynthesis Ilg__Mitsuokella.s__Mitsuokella_multacida	0	0	0	0.00056949	0.00035708	0	0.00114236	0	0	0
PWY-6121: 5-aminoimidazole ribonucleotide biosynthesis Ilg__Peptostreptococcus.s__Peptostreptococcus_anaerobius	0	0	0	0	0	0	0	0	0	0.00020837
PWY-6121: 5-aminoimidazole ribonucleotide biosynthesis Ilg__Phascolarctobacterium.s__Phascolarctobacterium_succinatutens	0	0.00061173	0	0	0	0	0	0	0	0
PWY-6121: 5-aminoimidazole ribonucleotide biosynthesis I|unclassified	0	0.00380519	0	0.00245877	0	0.0101193	0	0	0.00082143	0
PWY-6122: 5-aminoimidazole ribonucleotide biosynthesis IIlg__Actinomyces.s__Actinomyces_turicensis	0.00013412	0	0	0	0	0.00209665	0	0	0.0014086	0
PWY-6122: 5-aminoimidazole ribonucleotide biosynthesis IIlg__Akkermansia.s__Akkermansia_muciniphila	0	0	0.00610046	0	0	0	0	0	0	0
PWY-6122: 5-aminoimidazole ribonucleotide biosynthesis IIlg__Alistipes.s__Alistipes_finegoldii	0	0	0	0	0.00020829	0	0	0	0	0
PWY-6122: 5-aminoimidazole ribonucleotide biosynthesis IIlg__Anaerococcus.s__Anaerococcus_vaginalis	0.00015278	0.00032371	0	0	0	0.00019661	0	0	0	0
PWY-6122: 5-aminoimidazole ribonucleotide biosynthesis IIlg__Bifidobacterium.s__Bifidobacterium_adolescentis	0.0019934	0	0.00016896	0.00344682	0.00726268	0	0.00061537	0	0.00355656	0
PWY-6122: 5-aminoimidazole ribonucleotide biosynthesis IIlg__Bifidobacterium.s__Bifidobacterium_longum	0.00041764	0	0	0	0	0	0	0	5.93E-05	0
PWY-6122: 5-aminoimidazole ribonucleotide biosynthesis IIlg__Blautia.s__Ruminococcus_gnavus	0.00401506	0	0.00094744	0	0	0	0	0	0	0
PWY-6122: 5-aminoimidazole ribonucleotide biosynthesis IIlg__Butyrivibrio.s__Butyrivibrio_crossotus	0	0.00031522	0	0	0	0	0	0	0	0
PWY-6122: 5-aminoimidazole ribonucleotide biosynthesis IIlg__Campylobacter.s__Campylobacter_hominis	0	0	0	0.0003102	0	0	0.00023079	0	0	0

PWY-6122: 5-aminoimidazole ribonucleotide biosynthesis IIlg__Campylobacter.s__Campylobacter_ureolyticus	0	0	0	0	0.00022305	0	0	0	0	0
PWY-6122: 5-aminoimidazole ribonucleotide biosynthesis IIlg__Catenibacterium.s__Catenibacterium_mitsuokai	0	0	0	0	0.00060259	0	0.00065286	0	0	0
PWY-6122: 5-aminoimidazole ribonucleotide biosynthesis IIlg__Clostridium.s__Clostridium_hathewayi	0	0	0	0	0	0	0	0	0	0.00053267
PWY-6122: 5-aminoimidazole ribonucleotide biosynthesis IIlg__Clostridium.s__Clostridium_nexile	0	0	0	0	0	0.00042037	0	0	0	0.00149618
PWY-6122: 5-aminoimidazole ribonucleotide biosynthesis IIlg__Collinsella.s__Collinsella_aerofaciens	0	0.00048608	0.00019296	0	0.00097806	0	0.00130658	0	0.00215006	0.00085971
PWY-6122: 5-aminoimidazole ribonucleotide biosynthesis IIlg__Desulfovibrio.s__Desulfovibrio_piger	0	0	0.00022694	0	0	0	0	0	0	0
PWY-6122: 5-aminoimidazole ribonucleotide biosynthesis IIlg__Dorea.s__Dorea_formicigenerans	0	0	0	0	0	0	0	0	0.00014133	0
PWY-6122: 5-aminoimidazole ribonucleotide biosynthesis IIlg__Dorea.s__Dorea_longicatena	0	0	0	0	0	0	0.00019357	0	0	0
PWY-6122: 5-aminoimidazole ribonucleotide biosynthesis IIlg__Eggerthella.s__Eggerthella_lenta	0	0	0.00015679	0	0	0	0	0	0	0
PWY-6122: 5-aminoimidazole ribonucleotide biosynthesis IIlg__Erysipelotrichaceae_noname.s__Erysipelotrichaceae_bacterium_6_1_45	0	0	0.00028929	0	0	0	0	0	0	0
PWY-6122: 5-aminoimidazole ribonucleotide biosynthesis IIlg__Erysipelotrichaceae_noname.s__Eubacterium_biforme	0	0.0004431	0	0.00036919	0	0	0.00030528	0	0.00013907	0.00033434
PWY-6122: 5-aminoimidazole ribonucleotide biosynthesis IIlg__Escherichia.s__Escherichia_coli	0	0	0.00019637	0	0	0	0	0	0	0
PWY-6122: 5-aminoimidazole ribonucleotide biosynthesis IIlg__Faecalibacterium.s__Faecalibacterium_prausnitzii	0.00017789	0.00065294	0	0.00042314	0.00046102	0	0.00105239	0	0.00119965	0.0005505
PWY-6122: 5-aminoimidazole ribonucleotide biosynthesis IIlg__Fusobacterium.s__Fusobacterium_gonidiaformans	0	0	0	0	0.00030056	0	0	0	0	0.00054106
PWY-6122: 5-aminoimidazole ribonucleotide biosynthesis IIlg__Fusobacterium.s__Fusobacterium_nucleatum	0	0	0	0	0	0.00023312	0	0	0	0
PWY-6122: 5-aminoimidazole ribonucleotide biosynthesis IIlg__Lachnospiraceae_noname.s__Lachnospiraceae_bacterium_2_1_58FAA	0	0	0.00130132	0	0	0	0	0	0	0
PWY-6122: 5-aminoimidazole ribonucleotide biosynthesis IIlg__Lactobacillus.s__Lactobacillus_ruminis	0.0008361	0	0	0	0.00107642	0	0	0	0	0
PWY-6122: 5-aminoimidazole ribonucleotide biosynthesis IIlg__Megamonas.s__Megamonas_rupellensis	0	0	0	0	0	0	0.0008295	0	0	0
PWY-6122: 5-aminoimidazole ribonucleotide biosynthesis IIlg__Megasphaera.s__Megasphaera_elsdenii	0	0	0	0	0.00055859	0	0	0	0	0
PWY-6122: 5-aminoimidazole ribonucleotide biosynthesis IIlg__Methanobrevibacter.s__Methanobrevibacter_smithii	0.00019668	0	0.00026597	0	0	0	0	0	0	0
PWY-6122: 5-aminoimidazole ribonucleotide biosynthesis IIlg__Mitsuokella.s__Mitsuokella_multacida	0	0	0	0.00054113	0.00033946	0	0.00100325	0	0	0
PWY-6122: 5-aminoimidazole ribonucleotide biosynthesis IIlg__Phascolarctobacterium.s__Phascolarctobacterium_succinatutens	0	0.00063927	0	0	0	0	0	0	0	0
PWY-6122: 5-aminoimidazole ribonucleotide biosynthesis IIlunclassified	0	0.00631364	0	0.00247606	0	0.0135543	0.00256962	0	0.00115296	0
PWY-6123: inosine-5'-phosphate biosynthesis I	0.0103889	0.00269944	0.00377047	0.00501027	0.00405633	0.00249713	0.0041813	0	0.00789914	0.00866349
PWY-6123: inosine-5'-phosphate biosynthesis Ilg__Bacteroides.s__Bacteroides_fragilis	0.00685626	0	0.00080128	0	0	0	0	0	0	0.00455042
PWY-6123: inosine-5'-phosphate biosynthesis Ilg__Escherichia.s__Escherichia_coli	0	0	0.00031565	0	0.00038304	0	0	0	0	0.00047545

PWY-6123: inosine-5'-phosphate biosynthesis I|g__Faecalibacterium.s__Faecalibacterium_prausnitzii	0.00018952	0.00067531	0	0.00071568	0.00022866	0	0.00052963	0	0.00220165	0.00050001
PWY-6123: inosine-5'-phosphate biosynthesis I|unclassified	0	0	0	0	0	0.00037853	0.00141332	0	0.00163281	0
PWY-6124: inosine-5'-phosphate biosynthesis II	0.00934679	0.002289	0.00332422	0.00363042	0.00262827	0.00178838	0.00284296	0	0.00562424	0.00771503
PWY-6124: inosine-5'-phosphate biosynthesis II|g__Bacteroides.s__Bacteroides_fragilis	0.00651409	0	0.00078728	0	0	0	0	0	0	0.00414783
PWY-6124: inosine-5'-phosphate biosynthesis II|g__Escherichia.s__Escherichia_coli	0	0	0.00047208	0	0.00085192	0	0	0	0	0.00047218
PWY-6124: inosine-5'-phosphate biosynthesis II|g__Faecalibacterium.s__Faecalibacterium_prausnitzii	0.0001648	0.00061017	0	0.00069426	0.00020309	0	0.00053279	0	0.00208717	0.00045851
PWY-6124: inosine-5'-phosphate biosynthesis II|g__Fusobacterium.s__Fusobacterium_nucleatum	0	0	0	0	0	0.00046473	0	0	0	0
PWY-6124: inosine-5'-phosphate biosynthesis II|unclassified	0	0	0	0	0	0.00031768	0.00128036	0	0	0
PWY-6125: superpathway of guanosine nucleotides de novo biosynthesis II|g__Bacteroides.s__Bacteroides_vulgatus	0	0	0	0	0.00069594	0	0	0	0	0
PWY-6125: superpathway of guanosine nucleotides de novo biosynthesis II|unclassified	0	0	0	0	0	0	0	0	0.00124992	0
PWY-6126: superpathway of adenosine nucleotides de novo biosynthesis II|g__Bacteroides.s__Bacteroides_vulgatus	0	0	0	0	0.00091182	0.00063496	0	0	0	0
PWY-6126: superpathway of adenosine nucleotides de novo biosynthesis II|g__Mitsuokella.s__Mitsuokella_multacida	0	0	0	0	0	0	0.00222758	0	0	0
PWY-6126: superpathway of adenosine nucleotides de novo biosynthesis II|unclassified	0	0	0	0	0.00209318	0	0	0	0.00141726	0
PWY-6147: 6-hydroxymethyl-dihydropterin diphosphate biosynthesis I|g__Collinsella.s__Collinsella_aerofaciens	0	0	0	0.0008624	0	0	0	0	0.00057957	0
PWY-6147: 6-hydroxymethyl-dihydropterin diphosphate biosynthesis I|g__Mitsuokella.s__Mitsuokella_multacida	0	0	0	0	0	0	0.00071397	0	0	0
PWY-6147: 6-hydroxymethyl-dihydropterin diphosphate biosynthesis I|g__Prevotella.s__Prevotella_buccalis	0	0	0	0	0	0	0	0	0.00065325	0
PWY-6147: 6-hydroxymethyl-dihydropterin diphosphate biosynthesis I|g__Prevotella.s__Prevotella_timonensis	0	0	0	0	0	0.00170308	0	0	0	0
PWY-6151: S-adenosyl-L-methionine cycle I|g__Actinomyces.s__Actinomyces_turicensis	0	0	0	0	0	0	0	0	0.00059924	0
PWY-6151: S-adenosyl-L-methionine cycle I|g__Bacteroides.s__Bacteroides_vulgatus	0	0	0.00043047	0	0.0012713	0.00072737	0	0	0	0
PWY-6151: S-adenosyl-L-methionine cycle I|g__Bifidobacterium.s__Bifidobacterium_adolescentis	0.00188761	0	0.00037291	0.00143527	0.00765476	0	0	0	0.0016704	0
PWY-6151: S-adenosyl-L-methionine cycle I|g__Blautia.s__Ruminococcus_gnavus	0.00491483	0	0.00182836	0	0	0	0	0	0	0
PWY-6151: S-adenosyl-L-methionine cycle I|g__Catenibacterium.s__Catenibacterium_mitsuokai	0	0.00089299	0	0	0	0	0	0	0	0
PWY-6151: S-adenosyl-L-methionine cycle I|g__Clostridium.s__Clostridium_nexile	0	0	0	0	0	0.00047455	0	0	0	0.00082117
PWY-6151: S-adenosyl-L-methionine cycle I|g__Collinsella.s__Collinsella_aerofaciens	0	0	0	0	0.00112673	0	0.00028594	0	0.00104258	0
PWY-6151: S-adenosyl-L-methionine cycle I|g__Dorea.s__Dorea_longicatena	0	0.00060234	0	0	0	0	0	0	0	0
PWY-6151: S-adenosyl-L-methionine cycle I|g__Erysipelotrichaceae_noname.s__Eubacterium_biforme	0.00019138	0	0	0.00073282	0.00045453	0	0	0	0.00020711	0.00032786
PWY-6151: S-adenosyl-L-methionine cycle I|g__Escherichia.s__Escherichia_coli	0	0	0.00081387	0	0	0	0	0	0	0.00024373
PWY-6151: S-adenosyl-L-methionine cycle I|g__Eubacterium.s__Eubacterium_rectale	0	0	0	0.00125723	0.00032973	0	0	0	0.00122755	0.00078375
PWY-6151: S-adenosyl-L-methionine cycle I|g__Faecalibacterium.s__Faecalibacterium_prausnitzii	0.0001865	0.0024811	0	0	0	0	0.00099514	0	0.00116867	0.00083163
PWY-6151: S-adenosyl-L-methionine cycle I|g__Lachnospiraceae_noname.s__Lachnospiraceae_bacterium_2_1_58FAA	0	0	0.0018995	0	0	0	0	0	0	0
PWY-6151: S-adenosyl-L-methionine cycle I|g__Lactobacillus.s__Lactobacillus_ruminis	0.00081929	0	0	0	0.00083204	0	0	0	0	0

PWY-6151: S-adenosyl-L-methionine cycle I|g__Prevotella.s__Prevotella_copri	0	0.00618435	0	0.0016856	0	0	0.00684887	0	0.00338895	0.00172976
PWY-6151: S-adenosyl-L-methionine cycle I|g__Prevotella.s__Prevotella_timonensis	0	0	0	0	0	0.0039928	0	0	0	0
PWY-6151: S-adenosyl-L-methionine cycle I|g__Roseburia.s__Roseburia_inulinivorans	0	0	0	0	0.00100471	0	0	0	0	0
PWY-6151: S-adenosyl-L-methionine cycle I|unclassified	0	0.00778663	0.00093302	0.0023226	0	0.00690925	0.00408666	0	0.00217713	0
PWY-6163: chorismate biosynthesis from 3-dehydroquinate|g__Akkermansia.s__Akkermansia_muciniphila	0	0	0.00387486	0	0	0	0	0	0	0
PWY-6163: chorismate biosynthesis from 3-dehydroquinate|g__Bacteroides.s__Bacteroides_fragilis	0.00773001	0	0	0	0	0	0	0	0	0
PWY-6163: chorismate biosynthesis from 3-dehydroquinate|g__Blautia.s__Ruminococcus_gnavus	0.0042981	0	0.00142586	0	0	0	0	0	0	0
PWY-6163: chorismate biosynthesis from 3-dehydroquinate|g__Catenibacterium.s__Catenibacterium_mitsuokai	0	0.00041786	0	0	0.00066063	0	0.00048628	0	0	0
PWY-6163: chorismate biosynthesis from 3-dehydroquinate|g__Escherichia.s__Escherichia_coli	0	0	0.00037483	0	0	0	0	0	0	0
PWY-6163: chorismate biosynthesis from 3-dehydroquinate|g__Eubacterium.s__Eubacterium_rectale	0.00032194	0	0	0.00094492	0	0	0	0	0.00171834	0
PWY-6163: chorismate biosynthesis from 3-dehydroquinate|g__Lactobacillus.s__Lactobacillus_ruminis	0.00074228	0	0	0	0.00153113	0	0	0	0	0
PWY-6163: chorismate biosynthesis from 3-dehydroquinate|g__Megamonas.s__Megamonas_rupellensis	0	0	0	0	0	0	0.00097093	0	0	0
PWY-6163: chorismate biosynthesis from 3-dehydroquinate|g__Mitsuokella.s__Mitsuokella_multacida	0	0	0	0	0	0	0.00133048	0	0	0
PWY-6163: chorismate biosynthesis from 3-dehydroquinate|g__Roseburia.s__Roseburia_inulinivorans	0	0	0	0	0.00122951	0	0	0	0	0
PWY-6163: chorismate biosynthesis from 3-dehydroquinate|unclassified	0	0.00742814	0	0.00387066	0	0	0.00388698	0	0.00267331	0
PWY-6168: flavin biosynthesis III (fungi)|unclassified	0	0.00277638	0	0	0	0	0	0	0	0
PWY-621: sucrose degradation III (sucrose invertase)	0.00546207	0.00409742	0.00095599	0.00548333	0.00739459	0	0.00709401	0	0.00538427	0.00352729
PWY-621: sucrose degradation III (sucrose invertase)|g__Blautia.s__Ruminococcus_torques	0	0	0	0	0	0	0.00178696	0	0	0
PWY-621: sucrose degradation III (sucrose invertase)|g__Eubacterium.s__Eubacterium_rectale	0.0011384	0	0	0	0.00085013	0	0	0	0.00143645	0.00063276
PWY-621: sucrose degradation III (sucrose invertase)|g__Faecalibacterium.s__Faecalibacterium_prausnitzii	0.00036893	0.00202365	0	0.00125833	0.0004297	0	0.00146977	0	0.00254004	0.00270018
PWY-621: sucrose degradation III (sucrose invertase)|g__Klebsiella.s__Klebsiella_pneumoniae	0	0	0	0.00033566	0	0	0	0	0	0
PWY-6263: superpathway of menaquinol-8 biosynthesis II	0	0.00110375	0	0	0.00081218	0	0	0	0	0
PWY-6277: superpathway of 5-aminoimidazole ribonucleotide biosynthesis|g__Actinomyces.s__Actinomyces_turicensis	0.00013412	0	0	0	0	0.00209665	0	0	0.0014086	0
PWY-6277: superpathway of 5-aminoimidazole ribonucleotide biosynthesis|g__Akkermansia.s__Akkermansia_muciniphila	0	0	0.00610046	0	0	0	0	0	0	0
PWY-6277: superpathway of 5-aminoimidazole ribonucleotide biosynthesis|g__Alistipes.s__Alistipes_finegoldii	0	0	0	0	0.00020829	0	0	0	0	0
PWY-6277: superpathway of 5-aminoimidazole ribonucleotide biosynthesis|g__Anaerococcus.s__Anaerococcus_vaginalis	0.00015278	0.00032371	0	0	0	0.00019661	0	0	0	0
PWY-6277: superpathway of 5-aminoimidazole ribonucleotide biosynthesis|g__Bifidobacterium.s__Bifidobacterium_adolescentis	0.0019934	0	0.00016896	0.00344682	0.00726268	0	0.00061537	0	0.00355656	0
PWY-6277: superpathway of 5-aminoimidazole ribonucleotide biosynthesis|g__Bifidobacterium.s__Bifidobacterium_longum	0.00041764	0	0	0	0	0	0	0	5.93E-05	0

PWY-6277: superpathway of 5-aminoimidazole ribonucleotide biosynthesis|g__Blautia.s__Ruminococcus_gnavus	0.00401506	0	0.00094744	0	0	0	0	0	0	0
PWY-6277: superpathway of 5-aminoimidazole ribonucleotide biosynthesis|g__Butyrivibrio.s__Butyrivibrio_crossotus	0	0.00031522	0	0	0	0	0	0	0	0
PWY-6277: superpathway of 5-aminoimidazole ribonucleotide biosynthesis|g__Campylobacter.s__Campylobacter_hominis	0	0	0	0.0003102	0	0	0.00023079	0	0	0
PWY-6277: superpathway of 5-aminoimidazole ribonucleotide biosynthesis|g__Campylobacter.s__Campylobacter_ureolyticus	0	0	0	0	0.00022305	0	0	0	0	0
PWY-6277: superpathway of 5-aminoimidazole ribonucleotide biosynthesis|g__Catenibacterium.s__Catenibacterium_mitsuokai	0	0	0	0	0.00060259	0	0.00065286	0	0	0
PWY-6277: superpathway of 5-aminoimidazole ribonucleotide biosynthesis|g__Clostridium.s__Clostridium_hathewayi	0	0	0	0	0	0	0	0	0	0.00053267
PWY-6277: superpathway of 5-aminoimidazole ribonucleotide biosynthesis|g__Clostridium.s__Clostridium_nexile	0	0	0	0	0	0.00042037	0	0	0	0.00149618
PWY-6277: superpathway of 5-aminoimidazole ribonucleotide biosynthesis|g__Collinsella.s__Collinsella_aerofaciens	0	0.00048608	0.00019296	0	0.00097806	0	0.00130658	0	0.00215006	0.00085971
PWY-6277: superpathway of 5-aminoimidazole ribonucleotide biosynthesis|g__Desulfovibrio.s__Desulfovibrio_piger	0	0	0.00022694	0	0	0	0	0	0	0
PWY-6277: superpathway of 5-aminoimidazole ribonucleotide biosynthesis|g__Dorea.s__Dorea_formicigenerans	0	0	0	0	0	0	0	0	0.00014133	0
PWY-6277: superpathway of 5-aminoimidazole ribonucleotide biosynthesis|g__Dorea.s__Dorea_longicatena	0	0	0	0	0	0	0.00019357	0	0	0
PWY-6277: superpathway of 5-aminoimidazole ribonucleotide biosynthesis|g__Eggerthella.s__Eggerthella_lenta	0	0	0.00015679	0	0	0	0	0	0	0
PWY-6277: superpathway of 5-aminoimidazole ribonucleotide biosynthesis|g__Erysipelotrichaceae_noname.s__Erysipelotrichaceae_bacterium_6_1_45	0	0	0.00028929	0	0	0	0	0	0	0
PWY-6277: superpathway of 5-aminoimidazole ribonucleotide biosynthesis|g__Erysipelotrichaceae_noname.s__Eubacterium_biforme	0	0.0004431	0	0.00036919	0	0	0.00030528	0	0.00013907	0.00033434
PWY-6277: superpathway of 5-aminoimidazole ribonucleotide biosynthesis|g__Escherichia.s__Escherichia_coli	0	0	0.00019637	0	0	0	0	0	0	0
PWY-6277: superpathway of 5-aminoimidazole ribonucleotide biosynthesis|g__Faecalibacterium.s__Faecalibacterium_prausnitzii	0.00017789	0.00065294	0	0.00042314	0.00046102	0	0.00105239	0	0.00119965	0.0005505
PWY-6277: superpathway of 5-aminoimidazole ribonucleotide biosynthesis|g__Fusobacterium.s__Fusobacterium_gonidiaformans	0	0	0	0	0.00030056	0	0	0	0	0.00054106
PWY-6277: superpathway of 5-aminoimidazole ribonucleotide biosynthesis|g__Fusobacterium.s__Fusobacterium_nucleatum	0	0	0	0	0	0.00023312	0	0	0	0
PWY-6277: superpathway of 5-aminoimidazole ribonucleotide biosynthesis|g__Lachnospiraceae_noname.s__Lachnospiraceae_bacterium_2_1_58FAA	0	0	0.00130132	0	0	0	0	0	0	0
PWY-6277: superpathway of 5-aminoimidazole ribonucleotide biosynthesis|g__Lactobacillus.s__Lactobacillus_ruminis	0.0008361	0	0	0	0.00107642	0	0	0	0	0
PWY-6277: superpathway of 5-aminoimidazole ribonucleotide biosynthesis|g__Megamonas.s__Megamonas_rupellensis	0	0	0	0	0	0	0.0008295	0	0	0

PWY-6277: superpathway of 5-aminoimidazole ribonucleotide biosynthesis|g__Megasphaera.s__Megasphaera_elsdenii	0	0	0	0	0.00055859	0	0	0	0	0
PWY-6277: superpathway of 5-aminoimidazole ribonucleotide biosynthesis|g__Methanobrevibacter.s__Methanobrevibacter_smithii	0.00019668	0	0.00026597	0	0	0	0	0	0	0
PWY-6277: superpathway of 5-aminoimidazole ribonucleotide biosynthesis|g__Mitsuokella.s__Mitsuokella_multacida	0	0	0	0.00054113	0.00033946	0	0.00100325	0	0	0
PWY-6277: superpathway of 5-aminoimidazole ribonucleotide biosynthesis|g__Phascolarctobacterium.s__Phascolarctobacterium_succinatutens	0	0.00063927	0	0	0	0	0	0	0	0
PWY-6277: superpathway of 5-aminoimidazole ribonucleotide biosynthesis|unclassified	0	0.00631364	0	0.00247606	0	0.0135543	0.00256962	0	0.00115296	0
PWY-6282: palmitoleate biosynthesis I (from (5Z)-dodec-5-enoate)	0.00069059	0.00190708	0.00191634	0	0.00171023	0	0.00059946	0	0	0.00069548
PWY-6282: palmitoleate biosynthesis I (from (5Z)-dodec-5-enoate)|g__Escherichia.s__Escherichia_coli	0	0	0	0	0.0005575	0	0	0	0	0
PWY-6284: superpathway of unsaturated fatty acids biosynthesis (E. coli)	0	0	0	0	0.0018687	0	0	0	0	0.00076796
PWY-6305: putrescine biosynthesis IV	0.00040671	0.00243572	0.00266542	0.0014791	0.0018229	0	0.00463784	0	0.00021404	0.00144209
PWY-6305: putrescine biosynthesis IV|g__Blautia.s__Ruminococcus_obeum	0	0	0	0	0	0	0	0	0	0.0005457
PWY-6305: putrescine biosynthesis IV|g__Blautia.s__Ruminococcus_torques	0	0	0	0	0	0	0.00044619	0	0	0
PWY-6305: putrescine biosynthesis IV|g__Desulfovibrio.s__Desulfovibrio_piger	0	0	0.000512	0	0	0	0	0	0	0
PWY-6305: putrescine biosynthesis IV|g__Escherichia.s__Escherichia_coli	0.00012888	0	0.00049289	0	0.00083633	0	0	0	0	0
PWY-6305: putrescine biosynthesis IV|g__Faecalibacterium.s__Faecalibacterium_prausnitzii	0	0.00060288	0	0	0.0002881	0	0.00025557	0	0	0.00037907
PWY-6305: putrescine biosynthesis IV|g__Megamonas.s__Megamonas_hypermegale	0	0	0	0	0	0	0.0005947	0	0	0
PWY-6305: putrescine biosynthesis IV|g__Megamonas.s__Megamonas_rupellensis	0	0	0	0	0	0	0.00053182	0	0	0
PWY-6317: galactose degradation I (Leloir pathway)	0.00400042	0.00506569	0.00475258	0.00576129	0.00517392	0.00482933	0.00735485	0	0.00543454	0.00681829
PWY-6317: galactose degradation I (Leloir pathway)|g__Escherichia.s__Escherichia_coli	0	0	0.00063194	0	0	0	0	0	0	0
PWY-6317: galactose degradation I (Leloir pathway)|g__Faecalibacterium.s__Faecalibacterium_prausnitzii	0.00024187	0.00125034	0	0.00074654	0.00051221	0	0.00063836	0	0.00171059	0.00135029
PWY-6317: galactose degradation I (Leloir pathway)|g__Fusobacterium.s__Fusobacterium_gonidiaformans	0	0	0	0	0	0	0	0	0	0.00056366
PWY-6317: galactose degradation I (Leloir pathway)|g__Megamonas.s__Megamonas_rupellensis	0	0	0	0	0	0	0.00046486	0	0	0
PWY-6317: galactose degradation I (Leloir pathway)|g__Mitsuokella.s__Mitsuokella_multacida	0	0	0	0.00077125	0.00107337	0	0.00166227	0	0	0
PWY-6353: purine nucleotides degradation II (aerobic)	0.00203752	0.00148394	0.0018321	0.00166951	0.00096591	0.0025525	0.00160233	0	0.00136743	0
PWY-6353: purine nucleotides degradation II (aerobic)|g__Escherichia.s__Escherichia_coli	0	0	0	0	0.00048602	0	0	0	0	0
PWY-6385: peptidoglycan biosynthesis III (mycobacteria)|g__Bacteroides.s__Bacteroides_fragilis	0.00774288	0	0.00076517	0	0	0	0	0	0.00035136	0.00418328
PWY-6385: peptidoglycan biosynthesis III (mycobacteria)|g__Escherichia.s__Escherichia_coli	0	0	0.00043693	0	0	0	0	0	0	0
PWY-6385: peptidoglycan biosynthesis III (mycobacteria)|g__Faecalibacterium.s__Faecalibacterium_prausnitzii	0	0.00084832	0	0	0.00035351	0	0.00042815	0	0.00204134	0.00133991
PWY-6385: peptidoglycan biosynthesis III (mycobacteria)|g__Peptoniphilus.s__Peptoniphilus_duerdenii	0	0	0	0	0	0.00064633	0	0	0	0
PWY-6385: peptidoglycan biosynthesis III (mycobacteria)|g__Porphyromonas.s__Porphyromonas_asaccharolytica	0	0	0	0	0	0.00084653	0	0	0.00055839	0
PWY-6386: UDP-N-acetylmuramoyl-pentapeptide biosynthesis II (lysine-	0	0	0	0	0	0.00118189	0	0	0.00074485	0

containing)|g__Actinomyces.s__Actinomyces_turicensis										
PWY-6386: UDP-N-acetylmuramoyl-pentapeptide biosynthesis II (lysine-containing)|g__Bacteroides.s__Bacteroides_fragilis	0.00751482	0	0.00085397	0	0	0	0	0	0.00034687	0.00417847
PWY-6386: UDP-N-acetylmuramoyl-pentapeptide biosynthesis II (lysine-containing)|g__Bifidobacterium.s__Bifidobacterium_adolescentis	0.00216798	0	0	0.00219143	0.00650525	0	0.0010415	0	0.00226692	0
PWY-6386: UDP-N-acetylmuramoyl-pentapeptide biosynthesis II (lysine-containing)|g__Blautia.s__Ruminococcus_gnavus	0.00409531	0	0.00174963	0	0	0	0	0	0	0
PWY-6386: UDP-N-acetylmuramoyl-pentapeptide biosynthesis II (lysine-containing)|g__Clostridium.s__Clostridium_hathewayi	0	0	0	0	0	0	0	0	0	0.00049256
PWY-6386: UDP-N-acetylmuramoyl-pentapeptide biosynthesis II (lysine-containing)|g__Collinsella.s__Collinsella_aerofaciens	0	0	0	0.00080317	0	0	0	0	0.00185149	0
PWY-6386: UDP-N-acetylmuramoyl-pentapeptide biosynthesis II (lysine-containing)|g__Erysipelotrichaceae_noname.s__Eubacterium_biforme	0	0	0	0	0	0	0.00025715	0	0	0
PWY-6386: UDP-N-acetylmuramoyl-pentapeptide biosynthesis II (lysine-containing)|g__Eubacterium.s__Eubacterium_rectale	0.00035472	0	0	0.00094026	0.00088228	0	0	0	0.00130859	0.00044108
PWY-6386: UDP-N-acetylmuramoyl-pentapeptide biosynthesis II (lysine-containing)|g__Faecalibacterium.s__Faecalibacterium_prausnitzii	0	0	0	0	0	0	0.00045933	0	0.00204499	0.00150202
PWY-6386: UDP-N-acetylmuramoyl-pentapeptide biosynthesis II (lysine-containing)|g__Fusobacterium.s__Fusobacterium_gonidiaformans	0	0	0	0	0	0	0	0	0	0.00036703
PWY-6386: UDP-N-acetylmuramoyl-pentapeptide biosynthesis II (lysine-containing)|g__Lachnospiraceae_noname.s__Lachnospiraceae_bacterium_2_1_58FAA	0	0	0.00121738	0	0	0	0	0	0	0
PWY-6386: UDP-N-acetylmuramoyl-pentapeptide biosynthesis II (lysine-containing)|g__Lactobacillus.s__Lactobacillus_ruminis	0.00057964	0	0	0	0.00057623	0	0	0	0	0
PWY-6386: UDP-N-acetylmuramoyl-pentapeptide biosynthesis II (lysine-containing)|g__Megasphaera.s__Megasphaera_elsdenii	0	0	0	0	0.00049429	0	0	0	0	0
PWY-6386: UDP-N-acetylmuramoyl-pentapeptide biosynthesis II (lysine-containing)|g__Mitsuokella.s__Mitsuokella_multacida	0	0	0	0.00056916	0.00074661	0	0.00139534	0	0	0
PWY-6386: UDP-N-acetylmuramoyl-pentapeptide biosynthesis II (lysine-containing)|g__Peptoniphilus.s__Peptoniphilus_duerdenii	0	0	0	0	0	0.00063668	0	0	0	0
PWY-6386: UDP-N-acetylmuramoyl-pentapeptide biosynthesis II (lysine-containing)|g__Peptoniphilus.s__Peptoniphilus_lacrimalis	0	0	0	0	0	0.00034555	0	0	0	0.00025053
PWY-6386: UDP-N-acetylmuramoyl-pentapeptide biosynthesis II (lysine-containing)|g__Porphyromonas.s__Porphyromonas_asaccharolytica	0	0	0	0	0.00023514	0.00090866	0	0	0.00050443	0
PWY-6386: UDP-N-acetylmuramoyl-pentapeptide biosynthesis II (lysine-containing)|unclassified	0	0.00716181	0	0.00326293	0	0	0.0024018	0	0.00193634	0
PWY-6387: UDP-N-acetylmuramoyl-pentapeptide biosynthesis I (meso-diaminopimelate containing)|g__Actinomyces.s__Actinomyces_turicensis	0	0	0	0	0	0.00115714	0	0	0.00076633	0
PWY-6387: UDP-N-acetylmuramoyl-pentapeptide biosynthesis I (meso-diaminopimelate containing)|g__Anaerococcus.s__Anaerococcus_vaginalis	0	0	0	0	0	0	0	0	0	0.00021874
PWY-6387: UDP-N-acetylmuramoyl-pentapeptide biosynthesis I (meso-diaminopimelate containing)|g__Bacteroides.s__Bacteroides_fragilis	0.00828094	0	0.00100845	0	0	0	0	0	0.00037233	0.00435418

PWY-6387: UDP-N-acetylmuramoyl-pentapeptide biosynthesis I (meso-diaminopimelate containing)|g__Bacteroides.s__Bacteroides_vulgatus	0	0	0.00084804	0	0.00083483	0.00047762	0	0	0	0
PWY-6387: UDP-N-acetylmuramoyl-pentapeptide biosynthesis I (meso-diaminopimelate containing)|g__Bifidobacterium.s__Bifidobacterium_adolescentis	0.0021002	0	0	0.00185019	0.00627904	0	0.00094334	0	0.00209424	0
PWY-6387: UDP-N-acetylmuramoyl-pentapeptide biosynthesis I (meso-diaminopimelate containing)|g__Blautia.s__Ruminococcus_gnavus	0.00400022	0	0.00145784	0	0	0	0	0	0	0
PWY-6387: UDP-N-acetylmuramoyl-pentapeptide biosynthesis I (meso-diaminopimelate containing)|g__Blautia.s__Ruminococcus_torques	0	0	0	0	0	0	0.00056322	0	0	0
PWY-6387: UDP-N-acetylmuramoyl-pentapeptide biosynthesis I (meso-diaminopimelate containing)|g__Clostridium.s__Clostridium_hathewayi	0	0	0	0	0	0	0	0	0	0.00045334
PWY-6387: UDP-N-acetylmuramoyl-pentapeptide biosynthesis I (meso-diaminopimelate containing)|g__Collinsella.s__Collinsella_aerofaciens	0	0	0	0.0005957	0	0	0	0	0.00163503	0
PWY-6387: UDP-N-acetylmuramoyl-pentapeptide biosynthesis I (meso-diaminopimelate containing)|g__Erysipelotrichaceae_noname.s__Eubacterium_biforme	0	0	0	0	0	0	0.00026231	0	0	0
PWY-6387: UDP-N-acetylmuramoyl-pentapeptide biosynthesis I (meso-diaminopimelate containing)|g__Escherichia.s__Escherichia_coli	0	0	0.00041025	0	0	0	0	0	0	0
PWY-6387: UDP-N-acetylmuramoyl-pentapeptide biosynthesis I (meso-diaminopimelate containing)|g__Eubacterium.s__Eubacterium_rectale	0.0003373	0	0	0.00083566	0.00069389	0	0	0	0.00116487	0.00037126
PWY-6387: UDP-N-acetylmuramoyl-pentapeptide biosynthesis I (meso-diaminopimelate containing)|g__Faecalibacterium.s__Faecalibacterium_prausnitzii	0.00017417	0.00119902	0	0	0.00040196	0	0.00049807	0	0.00224122	0.0013779
PWY-6387: UDP-N-acetylmuramoyl-pentapeptide biosynthesis I (meso-diaminopimelate containing)|g__Fusobacterium.s__Fusobacterium_gonidiaformans	0	0	0	0	0	0	0	0	0	0.00036181
PWY-6387: UDP-N-acetylmuramoyl-pentapeptide biosynthesis I (meso-diaminopimelate containing)|g__Lachnospiraceae_noname.s__Lachnospiraceae_bacterium_2_1_58FAA	0	0	0.00108847	0	0	0	0	0	0	0
PWY-6387: UDP-N-acetylmuramoyl-pentapeptide biosynthesis I (meso-diaminopimelate containing)|g__Lactobacillus.s__Lactobacillus_ruminis	0.0005256	0	0	0	0.00048969	0	0	0	0	0
PWY-6387: UDP-N-acetylmuramoyl-pentapeptide biosynthesis I (meso-diaminopimelate containing)|g__Megasphaera.s__Megasphaera_elsdenii	0	0	0	0	0.00044078	0	0	0	0	0
PWY-6387: UDP-N-acetylmuramoyl-pentapeptide biosynthesis I (meso-diaminopimelate containing)|g__Mitsuokella.s__Mitsuokella_multacida	0	0	0	0.00048889	0.00068402	0	0.00133491	0	0	0
PWY-6387: UDP-N-acetylmuramoyl-pentapeptide biosynthesis I (meso-diaminopimelate containing)|g__Peptoniphilus.s__Peptoniphilus_duerdenii	0	0	0	0	0	0.00087182	0	0	0	0
PWY-6387: UDP-N-acetylmuramoyl-pentapeptide biosynthesis I (meso-diaminopimelate containing)|g__Peptoniphilus.s__Peptoniphilus_lacrimalis	0	0	0	0	0	0.00035474	0	0	0	0.00023225
PWY-6387: UDP-N-acetylmuramoyl-pentapeptide biosynthesis I (meso-diaminopimelate containing)|g__Phascolarctobacterium.s__Phascolarctobacterium_succinatutens	0	0.00034062	0	0	0	0	0	0	0	0
PWY-6387: UDP-N-acetylmuramoyl-pentapeptide biosynthesis I (meso-diaminopimelate containing)|g__Porphyromonas.s__Porphyromonas_asaccharolytica	0	0	0	0	0.00022009	0.00074972	0	0	0.00051544	0
PWY-6387: UDP-N-acetylmuramoyl-pentapeptide biosynthesis I (meso-diaminopimelate containing)|unclassified	0	0.00706032	0	0.00310033	0	0	0.00227762	0	0.00184034	0
PWY-6471: peptidoglycan biosynthesis IV (Enterococcus faecium)	0.00204115	0	0	0	0	0	0	0	0	0
PWY-6507: 4-deoxy-L-threo-hex-4-enopyranuronate degradation	0.00027783	0.0035522	0.00137237	0.00215198	0.00181592	0	0.00235849	0	0.00500719	0.00364322

PWY-6507: 4-deoxy-L-threo-hex-4-enopyranuronate degradation|g__Escherichia.s__Escherichia_coli	0	0	0.0006698	0	0.00047328	0	0	0	0	0
PWY-6507: 4-deoxy-L-threo-hex-4-enopyranuronate degradation|g__Faecalibacterium.s__Faecalibacterium_prausnitzii	0	0.00243977	0	0.00074364	0.00106733	0	0.00129296	0	0.00309454	0.00176746
PWY-6519: 8-amino-7-oxononanoate biosynthesis I	0.00072933	0.00136786	0.00212141	0	0.00147411	0	0	0	0	0.0006002
PWY-6527: stachyose degradation|g__Dorea.s__Dorea_longicatena	0	0	0	0	0	0	0	0	0.0001179	0
PWY-6527: stachyose degradation|g__Escherichia.s__Escherichia_coli	0	0	0.00064846	0	0.00059346	0	0	0	0	0
PWY-6527: stachyose degradation|g__Faecalibacterium.s__Faecalibacterium_prausnitzii	0	0.00032602	0	0	0	0	0	0	0	0
PWY-6527: stachyose degradation|g__Megamonas.s__Megamonas_hypermegale	0	0	0	0	0	0	0.00096845	0	0	0
PWY-6527: stachyose degradation|g__Megamonas.s__Megamonas_rupellensis	0	0	0	0	0	0	0.0004363	0	0	0
PWY-6545: pyrimidine deoxyribonucleotides de novo biosynthesis III|g__Mitsuokella.s__Mitsuokella_multacida	0	0	0	0	0	0	0.00223634	0	0	0
PWY-6588: pyruvate fermentation to acetone	0.00068747	0	0.00027223	0	0	0	0	0	0	0.00111535
PWY-6595: superpathway of guanosine nucleotides degradation (plants)	0	0.00056571	0	0	0	0.00059955	0.00078005	0	0.00031322	0.00061627
PWY-6595: superpathway of guanosine nucleotides degradation (plants)|g__Anaerococcus.s__Anaerococcus_vaginalis	0	0.00032247	0	0	0	0	0	0	0	0
PWY-6606: guanosine nucleotides degradation II	0.00021754	0.00071384	0.00070507	0.00090402	0.0003482	0.00145256	0.00089573	0	0.00061866	0.00068141
PWY-6606: guanosine nucleotides degradation II|g__Anaerococcus.s__Anaerococcus_vaginalis	0	0.00031196	0	0	0	0	0	0	0	0
PWY-6608: guanosine nucleotides degradation III|g__Anaerococcus.s__Anaerococcus_vaginalis	0	0.00036805	0	0	0	0	0	0	0	0
PWY-6608: guanosine nucleotides degradation III|g__Blautia.s__Ruminococcus_torques	0	0	0	0	0	0	0.00030585	0	0	0
PWY-6608: guanosine nucleotides degradation III|g__Escherichia.s__Escherichia_coli	0	0.00019967	0.00084517	0	0.00071619	0	0	0	0	0
PWY-6608: guanosine nucleotides degradation III|g__Prevotella.s__Prevotella_timonensis	0	0	0	0	0	0.00253712	0	0	0	0
PWY-6609: adenine and adenosine salvage III|g__Bacteroides.s__Bacteroides_fragilis	0.00638918	0	0.00079657	0	0	0	0	0	0.00021801	0.00405124
PWY-6609: adenine and adenosine salvage III|g__Blautia.s__Ruminococcus_gnavus	0.00367564	0	0.00135822	0	0	0	0	0	0	0
PWY-6609: adenine and adenosine salvage III|g__Blautia.s__Ruminococcus_torques	0	0	0	0	0	0	0.00138126	0	0	0
PWY-6609: adenine and adenosine salvage III|g__Erysipelotrichaceae_noname.s__Eubacterium_biforme	0	0	0	0	0	0	0	0	0	0.00039846
PWY-6609: adenine and adenosine salvage III|g__Escherichia.s__Escherichia_coli	0	0	0	0	0.00034729	0	0	0	0	0
PWY-6609: adenine and adenosine salvage III|g__Faecalibacterium.s__Faecalibacterium_prausnitzii	0	0.00232477	0	0	0	0	0.00037511	0	0.00237953	0.00137714
PWY-6609: adenine and adenosine salvage III|g__Fusobacterium.s__Fusobacterium_gonidiaformans	0	0	0	0	0	0.00078538	0	0	0	0.00070382
PWY-6609: adenine and adenosine salvage III|g__Lachnospiraceae_noname.s__Lachnospiraceae_bacterium_2_1_58FAA	0	0	0.00030057	0	0	0	0	0	0	0
PWY-6609: adenine and adenosine salvage III|g__Megamonas.s__Megamonas_rupellensis	0	0	0	0	0	0	0.00193601	0	0	0
PWY-6609: adenine and adenosine salvage III|g__Mitsuokella.s__Mitsuokella_multacida	0	0	0	0	0	0	0.001484	0	0	0
PWY-6609: adenine and adenosine salvage III|g__Mobiluncus.s__Mobiluncus_curtisii	0	0	0	0	0	0	0	0	0.00046622	0
PWY-6609: adenine and adenosine salvage III|g__Peptoniphilus.s__Peptoniphilus_duerdenii	0	0	0	0	0	0.00141902	0	0	0	0
PWY-6609: adenine and adenosine salvage III|g__Peptoniphilus.s__Peptoniphilus_lacrimalis	0.00021336	0	0	0	0	0.00036393	0	0	0	0.00062696
PWY-6609: adenine and adenosine salvage III|g__Prevotella.s__Prevotella_timonensis	0	0	0	0	0	0.00236357	0.00048958	0	0	0.00043392
PWY-6628: superpathway of L-phenylalanine biosynthesis|g__Escherichia.s__Escherichia_coli	0	0	0.00046193	0	0	0	0	0	0	0

PWY-6630: superpathway of L-tyrosine biosynthesis	0.00105851	0	0.00426117	0.00430466	0.00145679	0	0	0	0	0
PWY-6630: superpathway of L-tyrosine biosynthesis|g__Escherichia.s__Escherichia_coli	0	0	0.00038303	0	0	0	0	0	0	0
PWY-6700: queuosine biosynthesis|g__Akkermansia.s__Akkermansia_muciniphila	0	0	0.00576294	0	0	0	0	0	0	0
PWY-6700: queuosine biosynthesis|g__Bacteroides.s__Bacteroides_fragilis	0.00704633	0	0.00152225	0	0	0	0	0	0	0.00651143
PWY-6700: queuosine biosynthesis|g__Bacteroides.s__Bacteroides_vulgatus	0	0	0.00053356	0	0.00065833	0	0	0	0	0
PWY-6700: queuosine biosynthesis|g__Butyrivibrio.s__Butyrivibrio_crossotus	0	0.00033022	0	0	0	0	0	0	0	0
PWY-6700: queuosine biosynthesis|g__Campylobacter.s__Campylobacter_hominis	0	0	0	0	0	0	0.00057143	0	0	0
PWY-6700: queuosine biosynthesis|g__Desulfovibrio.s__Desulfovibrio_piger	0	0	0.00021659	0	0	0	0	0	0	0
PWY-6700: queuosine biosynthesis|g__Escherichia.s__Escherichia_coli	0	0	0.00024362	0.00041232	0.00067412	0	0	0	0	0
PWY-6700: queuosine biosynthesis|g__Fusobacterium.s__Fusobacterium_gonidiaformans	0	0	0	0	0	0	0	0	0	0.00072269
PWY-6700: queuosine biosynthesis|g__Megamonas.s__Megamonas_rupellensis	0	0	0	0	0	0	0.00068863	0	0	0
PWY-6700: queuosine biosynthesis|g__Megasphaera.s__Megasphaera_elsdenii	0	0	0	0.0005631	0.00060866	0	0	0	0	0
PWY-6700: queuosine biosynthesis|g__Mitsuokella.s__Mitsuokella_multacida	0	0	0	0	0.00055677	0	0.00128305	0	0	0
PWY-6700: queuosine biosynthesis|g__Prevotella.s__Prevotella_copri	0	0.00751446	0	0.00329373	0	0	0.00549195	0	0.00417265	0.00103789
PWY-6700: queuosine biosynthesis|g__Prevotella.s__Prevotella_disiens	0	0	0	0	0	0.00057995	0	0	0	0
PWY-6700: queuosine biosynthesis|g__Prevotella.s__Prevotella_timonensis	0	0	0	0	0	0.00305057	0	0	0	0
PWY-6700: queuosine biosynthesis|unclassified	0	0.009656	0	0.00324657	0	0.00421195	0.00825358	0	0.00314587	0.00122445
PWY-6703: preQ0 biosynthesis|g__Akkermansia.s__Akkermansia_muciniphila	0	0	0.00509134	0	0	0	0	0	0	0
PWY-6703: preQ0 biosynthesis|g__Bacteroides.s__Bacteroides_fragilis	0.00691859	0	0.00144042	0	0	0	0	0	0	0.00334206
PWY-6703: preQ0 biosynthesis|g__Bacteroides.s__Bacteroides_vulgatus	0	0	0.00043067	0	0	0	0	0	0	0
PWY-6703: preQ0 biosynthesis|g__Blautia.s__Ruminococcus_torques	0	0	0	0	0	0	0.0004746	0	0	0
PWY-6703: preQ0 biosynthesis|g__Desulfovibrio.s__Desulfovibrio_piger	0	0	0.00033055	0	0	0	0	0	0	0
PWY-6703: preQ0 biosynthesis|g__Escherichia.s__Escherichia_coli	0	0	0.00090339	0	0	0	0	0	0	0
PWY-6703: preQ0 biosynthesis|g__Fusobacterium.s__Fusobacterium_gonidiaformans	0	0	0	0	0.00051564	0	0	0	0	0
PWY-6703: preQ0 biosynthesis|g__Megamonas.s__Megamonas_rupellensis	0	0	0	0	0	0	0.00046382	0	0	0
PWY-6703: preQ0 biosynthesis|g__Megasphaera.s__Megasphaera_elsdenii	0	0	0	0.00076523	0	0	0	0	0	0
PWY-6703: preQ0 biosynthesis|g__Mitsuokella.s__Mitsuokella_multacida	0	0	0	0	0.00041263	0	0.00137825	0	0	0
PWY-6703: preQ0 biosynthesis|g__Porphyromonas.s__Porphyromonas_asaccharolytica	0	0	0	0	0	0.00068048	0	0	0.00061429	0
PWY-6703: preQ0 biosynthesis|unclassified	0	0.00416609	0	0.00379603	0	0	0.00301395	0	0	0
PWY-6708: ubiquinol-8 biosynthesis (prokaryotic)	0	0	0.00079682	0	0.00057287	0	0	0	0	0
PWY-6708: ubiquinol-8 biosynthesis (prokaryotic)|g__Escherichia.s__Escherichia_coli	0	0	0.0004846	0	0.0004504	0	0	0	0	0

PWY-6731: starch degradation III	0.00034321	0	0.00056919	0	0.00116495	0	0	0	0	0
PWY-6737: starch degradation V|g__Akkermansia.s__Akkermansia_muciniphila	0	0	0.00483426	0	0	0	0	0	0	0
PWY-6737: starch degradation V|g__Blautia.s__Ruminococcus_gnavus	0.00349496	0	0.00200663	0	0	0	0	0	0	0
PWY-6737: starch degradation V|g__Blautia.s__Ruminococcus_torques	0	0	0	0	0	0	0.00060984	0	0	0
PWY-6737: starch degradation V|g__Butyrivibrio.s__Butyrivibrio_crossotus	0	0.00034238	0	0	0	0	0	0	0	0
PWY-6737: starch degradation V|g__Catenibacterium.s__Catenibacterium_mitsuokai	0.00071992	0.00097779	0	0	0.00106039	0	0.00057293	0	0.00015735	0.00020793
PWY-6737: starch degradation V|g__Clostridium.s__Clostridium_hathewayi	0	0	0	0	0	0	0	0	0	0.00058716
PWY-6737: starch degradation V|g__Clostridium.s__Clostridium_nexile	0	0	0	0	0	0.00049113	0	0	0	0.0015043
PWY-6737: starch degradation V|g__Collinsella.s__Collinsella_aerofaciens	0	0	0	0.00029677	0	0	0.00064432	0	0.00094329	0.0008282
PWY-6737: starch degradation V|g__Coprococcus.s__Coprococcus_catus	0	0.00032849	0	0	0	0	0	0	0	0
PWY-6737: starch degradation V|g__Dorea.s__Dorea_formicigenerans	0	0	0	0	0	0	0.00023017	0	0	0.00023977
PWY-6737: starch degradation V|g__Dorea.s__Dorea_longicatena	0	0.00052075	0	0	0.00043707	0	0.00030496	0	0	0
PWY-6737: starch degradation V|g__Erysipelotrichaceae_noname.s__Eubacterium_biforme	0	0	0	0	0	0	0.00019258	0	0.00018165	0.00017454
PWY-6737: starch degradation V|g__Escherichia.s__Escherichia_coli	0	0	0.00027976	0	0.00015368	0	0	0	0	0
PWY-6737: starch degradation V|g__Eubacterium.s__Eubacterium_rectale	0.00023009	0	0	0.0016504	0.00159293	0	0	0	0.00078592	0.00065257
PWY-6737: starch degradation V|g__Eubacterium.s__Eubacterium_siraeum	0	0	0	0	0	0	0	0	0.00012391	0
PWY-6737: starch degradation V|g__Faecalibacterium.s__Faecalibacterium_prausnitzii	0.00021581	0.00193108	0	0.00102277	0.00092741	0	0.0009232	0	0.00277027	0.0015566
PWY-6737: starch degradation V|g__Fusobacterium.s__Fusobacterium_gonidiaformans	0.00021123	0	0	0	0	0	0	0	0	0.00113645
PWY-6737: starch degradation V|g__Lachnospiraceae_noname.s__Lachnospiraceae_bacterium_2_1_58FAA	0	0	0.00173906	0	0	0	0	0	0	0
PWY-6737: starch degradation V|g__Megamonas.s__Megamonas_rupellensis	0	0	0	0	0	0	0.00085616	0	0	0
PWY-6737: starch degradation V|g__Roseburia.s__Roseburia_inulinivorans	0	0.00128104	0	0	0.00102129	0	0	0	0.00012056	0
PWY-6737: starch degradation V|g__Ruminococcus.s__Ruminococcus_bromii	0.00023672	0	0.00013363	0	0	0	0	0	0	0.00033293
PWY-6737: starch degradation V|unclassified	0	0.00482272	0	0.00280673	0	0	0	0	0.00330839	0
PWY-6803: phosphatidylcholine acyl editing	0	0	0.00028905	0	0	0	0	0	0	0
PWY-6803: phosphatidylcholine acyl editing|g__Escherichia.s__Escherichia_coli	0	0	0.00025701	0	0	0	0	0	0	0
PWY-6823: molybdenum cofactor biosynthesis	0	0	0.00061513	0	0	0	0	0	0	0
PWY-6823: molybdenum cofactor biosynthesis|g__Escherichia.s__Escherichia_coli	0	0	0.00042348	0	0	0	0	0	0	0
PWY-6859: all-trans-farnesol biosynthesis|g__Escherichia.s__Escherichia_coli	0.00020895	0	0.00052334	0	0	0	0	0	0	0.0002878
PWY-6891: thiazole biosynthesis II (Bacillus)	0.00146299	0	0.0043448	0	0	0	0	0	0	0
PWY-6892: thiazole biosynthesis I (E. coli)	0.00179006	0	0.00495122	0.00093225	0.00406278	0	0	0	0	0.00054425
PWY-6892: thiazole biosynthesis I (E. coli)|g__Escherichia.s__Escherichia_coli	0.00036574	0	0.000366	0	0	0	0	0	0	0
PWY-6895: superpathway of thiamin diphosphate biosynthesis II	0.00462344	0	0.00249709	0	0	0	0	0	0	0
PWY-6897: thiamin salvage II|g__Escherichia.s__Escherichia_coli	0	0	0.00027011	0	0	0	0	0	0	0.00027437
PWY-6901: superpathway of glucose and xylose degradation	0.0007346	0.00067085	0.00263448	0.00215779	0.00308535	0	0	0	0	0
PWY-6936: seleno-amino acid biosynthesis|g__Erysipelotrichaceae_noname.s__Eubacterium_biforme	0	0	0	0	0	0	0.00053208	0	0.00020415	0

PWY-6936: seleno-amino acid biosynthesis|g__Escherichia.s__Escherichia_coli	0	0	0.00087837	0	0.00039009	0	0	0	0	0
PWY-6936: seleno-amino acid biosynthesis|g__Mitsuokella.s__Mitsuokella_multacida	0	0	0	0.00205955	0.00034186	0	0.00222243	0	0	0
PWY-6936: seleno-amino acid biosynthesis|g__Peptoniphilus.s__Peptoniphilus_lacrimalis	0	0	0	0	0	0.00071943	0	0	0	0
PWY-6969: TCA cycle V (2-oxoglutarate:ferredoxin oxidoreductase)|g__Escherichia.s__Escherichia_coli	0	0	0.00090186	0.00067823	0.00053488	0	0	0	0	0
PWY-7003: glycerol degradation to butanol	0.00090679	0	0	0	0	0	0	0	0	0
PWY-7013: L-1,2-propanediol degradation	0.00017225	0	0	0.00200373	0.00181713	0	0	0	0	0
PWY-7013: L-1,2-propanediol degradation|g__Escherichia.s__Escherichia_coli	0.00013382	0	0	0	0.00081311	0	0	0	0	0
PWY-7111: pyruvate fermentation to isobutanol (engineered)|g__Actinomyces.s__Actinomyces_turicensis	0	0	0	0	0	0.00119669	0	0	0.00073136	0
PWY-7111: pyruvate fermentation to isobutanol (engineered)|g__Akkermansia.s__Akkermansia_muciniphila	0	0	0.00615921	0	0	0	0	0	0	0
PWY-7111: pyruvate fermentation to isobutanol (engineered)|g__Bacteroides.s__Bacteroides_vulgatus	0	0	0.00045913	0.00027739	0.00071489	0.00060547	0	0	9.72E-05	0
PWY-7111: pyruvate fermentation to isobutanol (engineered)|g__Bifidobacterium.s__Bifidobacterium_longum	0.00048928	0	0	0	0.00038062	0	0	0	0	0
PWY-7111: pyruvate fermentation to isobutanol (engineered)|g__Blautia.s__Ruminococcus_torques	0	0	0	0	0	0	0.00115201	0	0	0
PWY-7111: pyruvate fermentation to isobutanol (engineered)|g__Butyrivibrio.s__Butyrivibrio_crossotus	0	0.00039988	0	0	0	0	0	0	0	0
PWY-7111: pyruvate fermentation to isobutanol (engineered)|g__Campylobacter.s__Campylobacter_hominis	0	0	0	0	0	0	0.00015107	0	0	0
PWY-7111: pyruvate fermentation to isobutanol (engineered)|g__Catenibacterium.s__Catenibacterium_mitsuokai	0.00050043	0.0005732	0	0	0.00074546	0	0.00084998	0	0	0
PWY-7111: pyruvate fermentation to isobutanol (engineered)|g__Clostridium.s__Clostridium_hathewayi	0	0	0	0	0	0	0	0	0	0.00061863
PWY-7111: pyruvate fermentation to isobutanol (engineered)|g__Clostridium.s__Clostridium_nexile	0	0	0	0	0	0.00045781	0	0	0	0.00147809
PWY-7111: pyruvate fermentation to isobutanol (engineered)|g__Desulfovibrio.s__Desulfovibrio_piger	0	0	0.00032687	0	0	0	0	0	0	0
PWY-7111: pyruvate fermentation to isobutanol (engineered)|g__Dorea.s__Dorea_formicigenerans	0.00021691	0	0	0	0.00019152	0	0	0	0.00016854	0
PWY-7111: pyruvate fermentation to isobutanol (engineered)|g__Dorea.s__Dorea_longicatena	0.00011058	0	0	0	0	0	0.00017284	0	0	0
PWY-7111: pyruvate fermentation to isobutanol (engineered)|g__Erysipelotrichaceae_noname.s__Erysipelotrichaceae_bacterium_6_1_45	0	0	0.00023405	0	0	0	0	0	0	0
PWY-7111: pyruvate fermentation to isobutanol (engineered)|g__Escherichia.s__Escherichia_coli	0.00025852	0	0.0007213	0	0.00097134	0	0	0	0	0.00033839
PWY-7111: pyruvate fermentation to isobutanol (engineered)|g__Eubacterium.s__Eubacterium_rectale	0.00024424	0	0.00013449	0.00164565	0.00036744	0	0	0	0.00115241	0
PWY-7111: pyruvate fermentation to isobutanol (engineered)|g__Faecalibacterium.s__Faecalibacterium_prausnitzii	0.0002131	0.00273149	0	0.00093227	0.00116735	0	0.00093202	0	0.00365944	0.00195109
PWY-7111: pyruvate fermentation to isobutanol (engineered)|g__Megamonas.s__Megamonas_hypermegale	0	0	0	0	0	0	0.00019332	0	0	0
PWY-7111: pyruvate fermentation to isobutanol (engineered)|g__Megamonas.s__Megamonas_rupellensis	0	0	0	0	0	0	0.0004512	0	0	0
PWY-7111: pyruvate fermentation to isobutanol (engineered)|g__Mitsuokella.s__Mitsuokella_multacida	0	0.00107085	0	0.00158344	0.00156486	0	0.00169618	0	0	0
PWY-7111: pyruvate fermentation to isobutanol (engineered)|g__Phascolarctobacterium.s__Phascolarctobacterium_succinatutens	0	0	0	0	0	0	0	0	0	0.00014095
PWY-7111: pyruvate fermentation to isobutanol (engineered)|g__Ruminococcus.s__Ruminococcus_bromii	0	0	0	0.00044185	0	0	0	0	0	0.00040023

PWY-7111: pyruvate fermentation to isobutanol (engineered)|unclassified	0	0.00822518	0	0.00483489	0	0.0131769	0.00337359	0	0.00147156	0
PWY-7184: pyrimidine deoxyribonucleotides de novo biosynthesis I|unclassified	0	0	0	0	0	0	0	0	0.00112624	0
PWY-7187: pyrimidine deoxyribonucleotides de novo biosynthesis II|unclassified	0	0	0	0	0	0	0	0	0.00130205	0
PWY-7196: superpathway of pyrimidine ribonucleosides salvage	0	0	0	0	0	0	0.00653788	0	0	0
PWY-7197: pyrimidine deoxyribonucleotide phosphorylation|g__Akkermansia.s__Akkermansia_muciniphila	0	0	0.00491596	0	0	0	0	0	0	0
PWY-7197: pyrimidine deoxyribonucleotide phosphorylation|g__Dialister.s__Dialister_micraerophilus	0	0	0	0	0	0	0	0.00139529	0	0
PWY-7197: pyrimidine deoxyribonucleotide phosphorylation|g__Mitsuokella.s__Mitsuokella_multacida	0	0	0	0	0	0	0.00197629	0	0	0
PWY-7197: pyrimidine deoxyribonucleotide phosphorylation|unclassified	0	0	0	0	0	0	0.00122054	0	0.0011745	0
PWY-7198: pyrimidine deoxyribonucleotides de novo biosynthesis IV	0	0	0	0	0	0.00050299	0	0	0	0.00025564
PWY-7199: pyrimidine deoxyribonucleosides salvage|g__Alistipes.s__Alistipes_finegoldii	0	0	0	0	0.00049146	0	0	0	0	0
PWY-7199: pyrimidine deoxyribonucleosides salvage|g__Anaerococcus.s__Anaerococcus_vaginalis	0	0.00066003	0	0	0	0	0	0	0	0
PWY-7199: pyrimidine deoxyribonucleosides salvage|unclassified	0	0	0	0	0	0	0	0	0.0011835	0
PWY-7200: superpathway of pyrimidine deoxyribonucleoside salvage	0.0010516	0	0	0	0	0	0	0	0	0
PWY-7204: pyridoxal 5'-phosphate salvage II (plants)	0.00139129	0.00268902	0.00127836	0.00134442	0.00144482	0	0	0	0	0.0050077
PWY-7208: superpathway of pyrimidine nucleobases salvage|g__Akkermansia.s__Akkermansia_muciniphila	0	0	0.00506027	0	0	0	0	0	0	0
PWY-7208: superpathway of pyrimidine nucleobases salvage|g__Blautia.s__Ruminococcus_torques	0	0	0	0	0	0	0.00080682	0	0	0
PWY-7208: superpathway of pyrimidine nucleobases salvage|g__Megamonas.s__Megamonas_hypermegale	0	0	0	0	0	0	0.00148634	0	0	0
PWY-7208: superpathway of pyrimidine nucleobases salvage|g__Mitsuokella.s__Mitsuokella_multacida	0	0	0	0	0	0	0.00148419	0	0	0
PWY-7208: superpathway of pyrimidine nucleobases salvage|unclassified	0	0	0	0	0.00165479	0	0	0	0.0014008	0
PWY-7209: superpathway of pyrimidine ribonucleosides degradation	0.00040842	0	0.0002475	0.00109408	0.00041453	0	0.00098989	0	0	0.00043702
PWY-7210: pyrimidine deoxyribonucleotides biosynthesis from CTP	0	0	0	0	0	0.00057047	0	0	0	0.00031272
PWY-7211: superpathway of pyrimidine deoxyribonucleotides de novo biosynthesis	0	0	0	0	0	0.00097915	0.00354951	0	0	0.00055533
PWY-7219: adenosine ribonucleotides de novo biosynthesis|g__Actinomyces.s__Actinomyces_turicensis	0.00017072	0	0	0	0	0.00175169	0	0	0.00051649	0
PWY-7219: adenosine ribonucleotides de novo biosynthesis|g__Akkermansia.s__Akkermansia_muciniphila	0	0	0.0052111	0	0	0	0	0	0	0
PWY-7219: adenosine ribonucleotides de novo biosynthesis|g__Alistipes.s__Alistipes_indistinctus	0	0	0	0	0.00025095	0	0	0	0	0
PWY-7219: adenosine ribonucleotides de novo biosynthesis|g__Bacteroides.s__Bacteroides_fragilis	0.00772289	0.0003215	0.00130378	0	0	0	0	0	0.00034475	0.00531547
PWY-7219: adenosine ribonucleotides de novo biosynthesis|g__Bacteroides.s__Bacteroides_uniformis	0	0	0	0	0	0	0	0	0.00014957	0
PWY-7219: adenosine ribonucleotides de novo biosynthesis|g__Bacteroides.s__Bacteroides_vulgatus	0	0	0.00090448	0	0.00152813	0.0007926	0	0	0	0
PWY-7219: adenosine ribonucleotides de novo biosynthesis|g__Bifidobacterium.s__Bifidobacterium_adolescentis	0.00209473	0	0.00044055	0.00508522	0.0104542	0	0.00180996	0	0.00431788	0
PWY-7219: adenosine ribonucleotides de novo biosynthesis|g__Bifidobacterium.s__Bifidobacterium_longum	0	0	0	0	0.0002864	0	0	0	0	0
PWY-7219: adenosine ribonucleotides de novo biosynthesis|g__Blautia.s__Ruminococcus_gnavus	0.00441181	0	0.00196193	0	0	0	0	0	0	0
PWY-7219: adenosine ribonucleotides de novo biosynthesis|g__Blautia.s__Ruminococcus_torques	0	0	0	0	0	0	0.00115286	0	0	0
PWY-7219: adenosine ribonucleotides de novo biosynthesis|g__Campylobacter.s__Campylobacter_hominis	0	0	0	0	0	0	0.00024202	0	0	0

PWY-7219: adenosine ribonucleotides de novo biosynthesis|g__Catenibacterium.s__Catenibacterium_mitsuokai	0.00020188	0.00124357	0	0.0003978	0.00164705	0	0.00076476	0	0	0
PWY-7219: adenosine ribonucleotides de novo biosynthesis|g__Clostridium.s__Clostridium_hathewayi	0	0	0	0	0	0	0	0	0	0.00026626
PWY-7219: adenosine ribonucleotides de novo biosynthesis|g__Clostridium.s__Clostridium_nexile	0	0	0	0	0	0.00117126	0	0	0	0.00187719
PWY-7219: adenosine ribonucleotides de novo biosynthesis|g__Clostridium.s__Clostridium_symbiosum	0	0	0.00019378	0	0	0	0	0	0	0
PWY-7219: adenosine ribonucleotides de novo biosynthesis|g__Collinsella.s__Collinsella_aerofaciens	0.00033677	0.00085584	0.00019657	0.00046044	0.00081788	0	0.00104318	0	0.00243414	0.00105279
PWY-7219: adenosine ribonucleotides de novo biosynthesis|g__Coprococcus.s__Coprococcus_comes	0	0	0	0	0	0	0	0	0.0002188	0
PWY-7219: adenosine ribonucleotides de novo biosynthesis|g__Dorea.s__Dorea_formicigenerans	0.00014595	0.00035217	0	0.0004298	0	0	0	0	0.00028642	0
PWY-7219: adenosine ribonucleotides de novo biosynthesis|g__Dorea.s__Dorea_longicatena	0	0	0	0	0.0002195	0	0.00027017	0	0	0
PWY-7219: adenosine ribonucleotides de novo biosynthesis|g__Eggerthella.s__Eggerthella_lenta	0	0	0.00018244	0	0	0	0	0	0	0
PWY-7219: adenosine ribonucleotides de novo biosynthesis|g__Erysipelotrichaceae_noname.s__Eubacterium_biforme	0.00015553	0.00074969	0	0.00040774	0	0	0.00048719	0	0.00018667	0.00045567
PWY-7219: adenosine ribonucleotides de novo biosynthesis|g__Escherichia.s__Escherichia_coli	0	0	0.00096945	0	0.00022599	0	0	0	0	0.0005718
PWY-7219: adenosine ribonucleotides de novo biosynthesis|g__Eubacterium.s__Eubacterium_rectale	0.00050024	0	0	0.00141545	0.00146906	0	0	0	0.00311599	0.001669
PWY-7219: adenosine ribonucleotides de novo biosynthesis|g__Faecalibacterium.s__Faecalibacterium_prausnitzii	0.00044958	0.00265147	0	0.00181964	0.00047976	0	0.00143073	0	0.00472285	0.00217988
PWY-7219: adenosine ribonucleotides de novo biosynthesis|g__Finegoldia.s__Finegoldia_magna	0.00027393	0.00024478	0	0.00114074	0	0.00056341	0	0.00253494	0	0
PWY-7219: adenosine ribonucleotides de novo biosynthesis|g__Fusobacterium.s__Fusobacterium_gonidiaformans	0	0	0	0	0.00042933	0.00018752	0	0	0	0.00085675
PWY-7219: adenosine ribonucleotides de novo biosynthesis|g__Fusobacterium.s__Fusobacterium_nucleatum	0	0	0	0	0	0.00045934	0	0	0	0
PWY-7219: adenosine ribonucleotides de novo biosynthesis|g__Granulicatella.s__Granulicatella_elegans	0.00041316	0	0	0	0	0	0	0	0	0
PWY-7219: adenosine ribonucleotides de novo biosynthesis|g__Lachnospiraceae_noname.s__Lachnospiraceae_bacterium_2_1_58FAA	0	0	0.00164665	0	0	0	0	0	0	0
PWY-7219: adenosine ribonucleotides de novo biosynthesis|g__Lactobacillus.s__Lactobacillus_ruminis	0.00076814	0	0	0	0.00102024	0	0.00027369	0	0	0
PWY-7219: adenosine ribonucleotides de novo biosynthesis|g__Megamonas.s__Megamonas_hypermegale	0	0	0	0	0	0	0.00061804	0	0	0
PWY-7219: adenosine ribonucleotides de novo biosynthesis|g__Megamonas.s__Megamonas_rupellensis	0	0	0	0	0	0	0.00113643	0	0	0
PWY-7219: adenosine ribonucleotides de novo biosynthesis|g__Megasphaera.s__Megasphaera_elsdenii	0	0	0	0.00126675	0.00096912	0	0	0	0	0
PWY-7219: adenosine ribonucleotides de novo biosynthesis|g__Methanobrevibacter.s__Methanobrevibacter_smithii	0.00034854	0	0	0	0	0	0	0	0	0
PWY-7219: adenosine ribonucleotides de novo biosynthesis|g__Mitsuokella.s__Mitsuokella_multacida	0	0.00058926	0	0.00108368	0.00140788	0	0.00260689	0	0	0
PWY-7219: adenosine ribonucleotides de novo biosynthesis|g__Parabacteroides.s__Parabacteroides_merdae	0	0	0	0	0	0	0	0	0.00013743	0
PWY-7219: adenosine ribonucleotides de novo biosynthesis|g__Phascolarctobacterium.s__Phascolarctobacterium_succinatutens	0	0.00034628	0.00012329	0	0	0	0.0001892	0	0	0
PWY-7219: adenosine ribonucleotides de novo biosynthesis|g__Porphyromonas.s__Porphyromonas_somerae	0.00012888	0	0	0	0	0	0	0	0	0
PWY-7219: adenosine ribonucleotides de novo biosynthesis|g__Prevotella.s__Prevotella_copri	0	0.00913966	0	0.00450701	0	0	0.0112204	0	0.00438475	0.00278328
PWY-7219: adenosine ribonucleotides de novo biosynthesis|g__Prevotella.s__Prevotella_disiens	0	0.00031619	0	0	0.00051869	0.00060762	0	0	0	0.00023511

PWY-7219: adenosine ribonucleotides de novo biosynthesis|g__Prevotella.s__Prevotella_stercorea	0	0	0	0	0	0	0	0	0.00067858	0
PWY-7219: adenosine ribonucleotides de novo biosynthesis|g__Roseburia.s__Roseburia_inulinivorans	0	0.00095463	0	0	0.00144554	0	0	0	0	0
PWY-7219: adenosine ribonucleotides de novo biosynthesis|g__Ruminococcus.s__Ruminococcus_bromii	0	0	0.00033177	0	0	0	0	0	0	0.00077196
PWY-7219: adenosine ribonucleotides de novo biosynthesis|g__Ruminococcus.s__Ruminococcus_callidus	0	0	0	0.00031531	0	0	0	0	0	0
PWY-7220: adenosine deoxyribonucleotides de novo biosynthesis II|g__Bacteroides.s__Bacteroides_vulgatus	0	0	0	0	0.00061451	0.00060261	0	0	0	0
PWY-7220: adenosine deoxyribonucleotides de novo biosynthesis II|g__Dialister.s__Dialister_micraerophilus	0	0	0	0	0	0	0	0.00040239	0	0
PWY-7220: adenosine deoxyribonucleotides de novo biosynthesis II|g__Eggerthella.s__Eggerthella_lenta	0	0	0.00022773	0	0	0	0	0	0	0
PWY-7220: adenosine deoxyribonucleotides de novo biosynthesis II|g__Mitsuokella.s__Mitsuokella_multacida	0	0	0	0	0	0	0.00211676	0	0	0
PWY-7221: guanosine ribonucleotides de novo biosynthesis|g__Akkermansia.s__Akkermansia_muciniphila	0	0	0.00585977	0	0	0	0	0	0	0
PWY-7221: guanosine ribonucleotides de novo biosynthesis|g__Bacteroides.s__Bacteroides_fragilis	0.00711361	0	0.0009801	0	0	0	0	0	0	0.00284701
PWY-7221: guanosine ribonucleotides de novo biosynthesis|g__Bacteroides.s__Bacteroides_vulgatus	0	0	0.00064595	0	0.00082135	0.00073929	0	0	0	0
PWY-7221: guanosine ribonucleotides de novo biosynthesis|g__Bifidobacterium.s__Bifidobacterium_adolescentis	0.00216275	0	0	0.0013444	0.00633725	0	0.00066819	0	0.00258335	0
PWY-7221: guanosine ribonucleotides de novo biosynthesis|g__Bifidobacterium.s__Bifidobacterium_longum	0.00034161	0	0	0	0	0	0	0	0.00011695	0
PWY-7221: guanosine ribonucleotides de novo biosynthesis|g__Bilophila.s__Bilophila_wadsworthia	0	0	0	0	0.00024008	0	0	0	0	0
PWY-7221: guanosine ribonucleotides de novo biosynthesis|g__Blautia.s__Ruminococcus_gnavus	0.00223389	0	0.00175537	0	0	0	0	0	0	0
PWY-7221: guanosine ribonucleotides de novo biosynthesis|g__Butyrivibrio.s__Butyrivibrio_crossotus	0	0.00052642	0	0	0	0	0	0	0	0
PWY-7221: guanosine ribonucleotides de novo biosynthesis|g__Campylobacter.s__Campylobacter_hominis	0	0	0	0	0	0	0.00021986	0	0	0
PWY-7221: guanosine ribonucleotides de novo biosynthesis|g__Clostridium.s__Clostridium_hathewayi	0	0	0	0	0	0	0	0	0	0.00062818
PWY-7221: guanosine ribonucleotides de novo biosynthesis|g__Clostridium.s__Clostridium_nexile	0	0	0	0	0	0.00036206	0	0	0	0.00044445
PWY-7221: guanosine ribonucleotides de novo biosynthesis|g__Clostridium.s__Clostridium_symbiosum	0	0	0.00017681	0	0	0	0	0	0	0
PWY-7221: guanosine ribonucleotides de novo biosynthesis|g__Coprococcus.s__Coprococcus_comes	0	0.00022297	0	0	0	0	0	0	0	0
PWY-7221: guanosine ribonucleotides de novo biosynthesis|g__Dialister.s__Dialister_micraerophilus	0.00014956	0	0	0	0	0	0	0	0	0
PWY-7221: guanosine ribonucleotides de novo biosynthesis|g__Enterococcus.s__Enterococcus_faecium	0.00013261	0	0	0	0	0	0	0	0	0
PWY-7221: guanosine ribonucleotides de novo biosynthesis|g__Escherichia.s__Escherichia_coli	0	0.00045178	0.00030246	0	0	0	0	0	0	0
PWY-7221: guanosine ribonucleotides de novo biosynthesis|g__Eubacterium.s__Eubacterium_rectale	0	0	0	0.0008009	0.00053057	0	0	0	0.00148554	0.00087296
PWY-7221: guanosine ribonucleotides de novo biosynthesis|g__Faecalibacterium.s__Faecalibacterium_prausnitzii	0.0001447	0.00080674	0	0	0.00063008	0	0.00036654	0	0.00052806	0.00137872
PWY-7221: guanosine ribonucleotides de novo biosynthesis|g__Finegoldia.s__Finegoldia_magna	0	0	0	0.00054625	0	0.00052656	0	0.00104646	0	0
PWY-7221: guanosine ribonucleotides de novo biosynthesis|g__Fusobacterium.s__Fusobacterium_gonidiaformans	0	0	0	0	0	0	0	0	0	0.00071921
PWY-7221: guanosine ribonucleotides de novo biosynthesis|g__Granulicatella.s__Granulicatella_elegans	0.00013855	0	0	0	0	0	0	0	0	0

PWY-7221: guanosine ribonucleotides de novo biosynthesis|g__Lachnospiraceae_noname.s__Lachnospiraceae_bacterium_2_1_58FAA	0	0	0.00124755	0	0	0	0	0	0	0
PWY-7221: guanosine ribonucleotides de novo biosynthesis|g__Lactobacillus.s__Lactobacillus_ruminis	0.0005667	0	0	0	0.00111238	0	0.0002029	0	0	0
PWY-7221: guanosine ribonucleotides de novo biosynthesis|g__Megasphaera.s__Megasphaera_elsdenii	0	0	0	0.00069297	0.00113384	0	0	0	0	0
PWY-7221: guanosine ribonucleotides de novo biosynthesis|g__Methanobrevibacter.s__Methanobrevibacter_smithii	0	0	0.00043196	0	0	0	0	0	0	0
PWY-7221: guanosine ribonucleotides de novo biosynthesis|g__Mitsuokella.s__Mitsuokella_multacida	0	0	0	0	0	0	0.00205292	0	0	0
PWY-7221: guanosine ribonucleotides de novo biosynthesis|g__Peptoniphilus.s__Peptoniphilus_duerdenii	0	0	0	0	0	0.00088702	0	0	0	0
PWY-7221: guanosine ribonucleotides de novo biosynthesis|g__Porphyromonas.s__Porphyromonas_asaccharolytica	0	0	0	0.00030071	0	0.00070117	0	0	0.00041361	0
PWY-7221: guanosine ribonucleotides de novo biosynthesis|g__Prevotella.s__Prevotella_copri	0	0.00845553	0	0.0030507	0	0	0.00728768	0	0.0033762	0.00197761
PWY-7221: guanosine ribonucleotides de novo biosynthesis|g__Prevotella.s__Prevotella_disiens	0.00014561	0	0	0	0	0.00025877	0	0	0	0.00021569
PWY-7221: guanosine ribonucleotides de novo biosynthesis|g__Prevotella.s__Prevotella_stercorea	0	0	0	0	0	0	0	0	0.00017453	0
PWY-7221: guanosine ribonucleotides de novo biosynthesis|g__Roseburia.s__Roseburia_inulinivorans	0	0.00066181	0	0	0	0	0	0	0	0
PWY-7221: guanosine ribonucleotides de novo biosynthesis|g__Ruminococcus.s__Ruminococcus_bromii	0	0	0	0	0	0	0	0	0	0.00052914
PWY-7221: guanosine ribonucleotides de novo biosynthesis|unclassified	0	0.0089875	0	0.00320086	0	0	0.0077742	0	0.00270749	0
PWY-7222: guanosine deoxyribonucleotides de novo biosynthesis II|g__Bacteroides.s__Bacteroides_vulgatus	0	0	0	0	0.00061451	0.00060261	0	0	0	0
PWY-7222: guanosine deoxyribonucleotides de novo biosynthesis II|g__Dialister.s__Dialister_micraerophilus	0	0	0	0	0	0	0	0.00040239	0	0
PWY-7222: guanosine deoxyribonucleotides de novo biosynthesis II|g__Eggerthella.s__Eggerthella_lenta	0	0	0.00022773	0	0	0	0	0	0	0
PWY-7222: guanosine deoxyribonucleotides de novo biosynthesis II|g__Mitsuokella.s__Mitsuokella_multacida	0	0	0	0	0	0	0.00211676	0	0	0
PWY-7228: superpathway of guanosine nucleotides de novo biosynthesis I|g__Akkermansia.s__Akkermansia_muciniphila	0	0	0.00517183	0	0	0	0	0	0	0
PWY-7228: superpathway of guanosine nucleotides de novo biosynthesis I|g__Bacteroides.s__Bacteroides_vulgatus	0	0	0.00064383	0	0.00076445	0.00069644	0	0	0	0
PWY-7228: superpathway of guanosine nucleotides de novo biosynthesis I|g__Mitsuokella.s__Mitsuokella_multacida	0	0	0	0	0	0	0.00210249	0	0	0
PWY-7228: superpathway of guanosine nucleotides de novo biosynthesis I|unclassified	0	0	0	0	0	0	0.00196914	0	0.0015268	0
PWY-7229: superpathway of adenosine nucleotides de novo biosynthesis I|g__Bacteroides.s__Bacteroides_vulgatus	0	0	0	0	0.0010704	0.0006486	0	0	0	0
PWY-7229: superpathway of adenosine nucleotides de novo biosynthesis I|g__Mitsuokella.s__Mitsuokella_multacida	0	0	0	0	0	0	0.00228095	0	0	0
PWY-7229: superpathway of adenosine nucleotides de novo biosynthesis I|unclassified	0	0	0	0	0.00240384	0	0	0	0.00165797	0
PWY-7234: inosine-5'-phosphate biosynthesis III	0.00771787	0.00175134	0.00123311	0.00501027	0.00405633	0.00249713	0.00287614	0	0.00789914	0.00110935
PWY-7234: inosine-5'-phosphate biosynthesis III|g__Escherichia.s__Escherichia_coli	0	0	0	0	0	0	0	0	0	0.00033479
PWY-7234: inosine-5'-phosphate biosynthesis III|unclassified	0	0	0	0	0	0	0	0	0.00163281	0
PWY-7235: superpathway of ubiquinol-6 biosynthesis (eukaryotic)	0	0	0	0	0.0005015	0	0	0	0	0
PWY-7237: myo-, chiro- and scillo-inositol degradation	0	0	0	0	0	0	0.00800228	0	0	0

PWY-7237: myo-, chiro- and scillo-inositol degradation|g__Megamonas.s__Megamonas_rupellensis	0	0	0	0	0	0	0.00408667	0	0	0
PWY-7242: D-fructuronate degradation	0.00083555	0.00360044	0.00113479	0.00224572	0.00178898	0	0.00234773	0	0.00518988	0.00373915
PWY-7242: D-fructuronate degradation|g__Escherichia.s__Escherichia_coli	0	0	0.00073722	0.00045136	0.00061492	0	0	0	0	0
PWY-7242: D-fructuronate degradation|g__Faecalibacterium.s__Faecalibacterium_prausnitzii	0.00018138	0.00203969	0	0.00118154	0.00095147	0	0.00136003	0	0.00280526	0.00180851
PWY-724: superpathway of L-lysine, L-threonine and L-methionine biosynthesis II|g__Faecalibacterium.s__Faecalibacterium_prausnitzii	0	0.00074799	0	0	0	0	0	0	0.00150608	0.00099015
PWY-724: superpathway of L-lysine, L-threonine and L-methionine biosynthesis II|g__Mitsuokella.s__Mitsuokella_multacida	0	0	0	0	0.00074128	0	0.0009446	0	0	0
PWY-724: superpathway of L-lysine, L-threonine and L-methionine biosynthesis II|unclassified	0	0	0	0	0	0.00890169	0.00269133	0	0	0
PWY-7254: TCA cycle VII (acetate-producers)	0	0.00086333	0.00105666	0.00177476	0.000366	0	0	0	0	0.00097874
PWY-7254: TCA cycle VII (acetate-producers)|g__Escherichia.s__Escherichia_coli	0	0	0.00060927	0	0.00025473	0	0	0	0	0
PWY-7269: NAD/NADP-NADH/NADPH mitochondrial interconversion (yeast)	0	0.00040588	0.0018863	0	0.00112712	0	0	0	0	0.0005839
PWY-7269: NAD/NADP-NADH/NADPH mitochondrial interconversion (yeast)|g__Escherichia.s__Escherichia_coli	0	0	0.00122142	0	0.00080539	0	0	0	0	0
PWY-7282: 4-amino-2-methyl-5-phosphomethylpyrimidine biosynthesis (yeast)|g__Bacteroides.s__Bacteroides_fragilis	0.00666584	0	0	0	0	0	0	0	0.00023619	0
PWY-7315: dTDP-N-acetylthomosamine biosynthesis	0.00052351	0	0.00363047	0.00124119	0.00274428	0	0	0	0	0.00065821
PWY-7315: dTDP-N-acetylthomosamine biosynthesis|g__Escherichia.s__Escherichia_coli	0.00016895	0	0.00057066	0.00049264	0.00063204	0	0	0	0	0.00025215
PWY-7323: superpathway of GDP-mannose-derived O-antigen building blocks biosynthesis|g__Escherichia.s__Escherichia_coli	0	0	0	0	0	0	0	0	0	0.00015051
PWY-7328: superpathway of UDP-glucose-derived O-antigen building blocks biosynthesis	0.00038381	0	0.00048003	0	0.00115863	0	0	0	0	0.00098176
PWY-7357: thiamin formation from pyrithiamine and oxythiamine (yeast)|g__Anaerococcus.s__Anaerococcus_vaginalis	0	0.00056001	0	0	0	0	0	0	0	0
PWY-7357: thiamin formation from pyrithiamine and oxythiamine (yeast)|g__Blautia.s__Ruminococcus_torques	0	0	0	0	0	0	0.00057731	0	0	0
PWY-7357: thiamin formation from pyrithiamine and oxythiamine (yeast)|g__Erysipelotrichaceae_noname.s__Eubacterium_biforme	0	0.00046107	0	0	0	0	0.00046055	0	0	0
PWY-7357: thiamin formation from pyrithiamine and oxythiamine (yeast)|g__Escherichia.s__Escherichia_coli	0	0	0.00028404	0	0.00071166	0	0	0	0	0.00031846
PWY-7357: thiamin formation from pyrithiamine and oxythiamine (yeast)|g__Faecalibacterium.s__Faecalibacterium_prausnitzii	0	0	0	0	0	0	0.00055186	0	0.00109078	0.00052156
PWY-7357: thiamin formation from pyrithiamine and oxythiamine (yeast)|g__Fusobacterium.s__Fusobacterium_nucleatum	0	0	0	0	0	0.00050946	0	0	0	0
PWY-7357: thiamin formation from pyrithiamine and oxythiamine (yeast)|g__Megamonas.s__Megamonas_rupellensis	0	0	0	0	0	0	0.00191893	0	0	0
PWY-7357: thiamin formation from pyrithiamine and oxythiamine (yeast)|g__Peptoniphilus.s__Peptoniphilus_duerdenii	0	0	0	0	0	0.00053938	0	0	0	0
PWY-7357: thiamin formation from pyrithiamine and oxythiamine (yeast)|g__Roseburia.s__Roseburia_inulinivorans	0	0.00053762	0	0	0.00088878	0	0	0	0	0
PWY-7371: 1,4-dihydroxy-6-naphthoate biosynthesis II	0	0.00075986	0	0.00088922	0.00109609	0	0.00300429	0	0	0
PWY-7371: 1,4-dihydroxy-6-naphthoate biosynthesis II|g__Megamonas.s__Megamonas_rupellensis	0	0	0	0	0	0	0.00075939	0	0	0
PWY-7371: 1,4-dihydroxy-6-naphthoate biosynthesis II|g__Mitsuokella.s__Mitsuokella_multacida	0	0.00071789	0	0.00079978	0.00087856	0	0.00168607	0	0	0

PWY-7383: anaerobic energy metabolism (invertebrates, cytosol)	0.00034368	0.00054663	0.00372177	0.00215394	0.00264516	0	0	0	0.00237244	0.0005291
PWY-7388: octanoyl-[acyl-carrier protein] biosynthesis (mitochondria, yeast)	0.00055136	0.00158114	0.00164213	0	0.00129673	0	0.00047226	0	0	0.00040789
PWY-7388: octanoyl-[acyl-carrier protein] biosynthesis (mitochondria, yeast)|g__Escherichia.s__Escherichia_coli	0	0	0	0	0.00045318	0	0	0	0	0
PWY-7392: taxadiene biosynthesis (engineered)	0	0	0	0	0	0	0	0	0	0.00096284
PWY-7400: L-arginine biosynthesis IV (archaebacteria)	0.00252422	0	0.00713953	0.00624541	0.00488688	0	0.00592805	0	0	0.00172979
PWY-7400: L-arginine biosynthesis IV (archaebacteria)|g__Escherichia.s__Escherichia_coli	0	0	0	0	0.0003672	0	0	0	0	0
PWY-7409: phospholipid remodeling (phosphatidylethanolamine, yeast)	0	0	0.00038016	0	0	0	0	0	0	0
PWY-7409: phospholipid remodeling (phosphatidylethanolamine, yeast)|g__Escherichia.s__Escherichia_coli	0	0	0.00038016	0	0	0	0	0	0	0
PWY-7446: sulfoglycolysis	0	0.00074157	0.00077737	0.00119109	0	0	0	0	0	0
PWY-7446: sulfoglycolysis|g__Escherichia.s__Escherichia_coli	0	0.00074157	0.00077737	0.00119109	0	0	0	0	0	0
PWY-7456: mannan degradation	0	0.00276741	0	0	0	0	0.00234679	0	0.00332325	0
PWY-7456: mannan degradation|g__Eubacterium.s__Eubacterium_siraeum	0	0.00022269	0	0	0	0	0	0	0	0
PWY-7539: 6-hydroxymethyl-dihydropterin diphosphate biosynthesis III (Chlamydia)|g__Escherichia.s__Escherichia_coli	0	0	0	0.00098576	0	0	0	0	0	0
PWY-7663: gondoate biosynthesis (anaerobic)	0.00822976	0.00964759	0.00369839	0.00653969	0.00476907	0.00250583	0.00827408	0	0.00855375	0.00810273
PWY-7663: gondoate biosynthesis (anaerobic)|g__Bacteroides.s__Bacteroides_fragilis	0.00588214	0	0.00084737	0	0	0	0	0	0	0.00281285
PWY-7663: gondoate biosynthesis (anaerobic)|g__Campylobacter.s__Campylobacter_hominis	0	0	0	0	0	0	0.00066899	0	0	0
PWY-7663: gondoate biosynthesis (anaerobic)|g__Escherichia.s__Escherichia_coli	0	0	0.00051749	0	0.00075426	0	0	0	0	0
PWY-7663: gondoate biosynthesis (anaerobic)|g__Prevotella.s__Prevotella_buccalis	0	0	0	0	0	0	0	0	0.00026054	0
PWY-7663: gondoate biosynthesis (anaerobic)|g__Prevotella.s__Prevotella_disiens	0	0.00045563	0	0	0	0	0	0	0	0.00028802
PWY-7664: oleate biosynthesis IV (anaerobic)	0.00079804	0.00217664	0.00213184	0	0.0017047	0	0.00069432	0	0	0.0005946
PWY-7664: oleate biosynthesis IV (anaerobic)|g__Escherichia.s__Escherichia_coli	0	0	0	0	0.00057964	0	0	0	0	0
PWY-821: superpathway of sulfur amino acid biosynthesis (Saccharomyces cerevisiae)	0	0	0.00258176	0	0	0	0	0	0	0
PWY-841: superpathway of purine nucleotides de novo biosynthesis I	0.00343519	0	0.00687744	0	0.00524832	0.00227637	0.00553181	0	0.00377929	0.00154141
PWY0-1061: superpathway of L-alanine biosynthesis	0.00181433	0.00244012	0.00321726	0.00238364	0.00293228	0	0.00053016	0	0	0.00095263
PWY0-1061: superpathway of L-alanine biosynthesis|g__Enterococcus.s__Enterococcus_faecium	0.00033604	0	0	0	0	0	0	0	0	0
PWY0-1061: superpathway of L-alanine biosynthesis|g__Escherichia.s__Escherichia_coli	0	0	0.00094503	0.00083682	0.00070738	0	0	0	0	0.00046543
PWY0-1241: ADP-L-glycero-β-D-manno-heptose biosynthesis	0	0.00065016	0.00096803	0	0.00043916	0	0	0	0	0
PWY0-1241: ADP-L-glycero-β-D-manno-heptose biosynthesis|g__Escherichia.s__Escherichia_coli	0	0.00043122	0.00063611	0	0.00038664	0	0	0	0	0
PWY0-1261: anhydromuropeptides recycling|g__Escherichia.s__Escherichia_coli	0.00028595	0.00038672	0.00110172	0.00057979	0	0	0	0	0	0
PWY0-1296: purine ribonucleosides degradation|g__Blautia.s__Ruminococcus_gnavus	0.00361401	0	0.00144805	0	0	0	0	0	0	0
PWY0-1296: purine ribonucleosides degradation|g__Blautia.s__Ruminococcus_torques	0	0	0	0	0	0	0.00132716	0	0	0
PWY0-1296: purine ribonucleosides degradation|g__Catenibacterium.s__Catenibacterium_mitsuokai	0.00065189	0.00064435	0	0	0.00088935	0	0.00026479	0	0	0
PWY0-1296: purine ribonucleosides degradation|g__Clostridium.s__Clostridium_hathewayi	0	0	0	0	0	0	0	0	0	0.00149958
PWY0-1296: purine ribonucleosides degradation|g__Clostridium.s__Clostridium_nexile	0	0	0	0	0	0	0	0	0	0.00037377

PWY0-1296: purine ribonucleosides degradation|g__Dorea.s__Dorea_formicigenerans	0	0.00049026	0	0	0	0	0	0	0.00025955	0
PWY0-1296: purine ribonucleosides degradation|g__Dorea.s__Dorea_longicatena	0	0.00096107	0	0	0	0	0.00033731	0	0	0
PWY0-1296: purine ribonucleosides degradation|g__Erysipelotrichaceae_noname.s__Eubacterium_biforme	0	0	0	0	0	0	0.00040092	0	0	0
PWY0-1296: purine ribonucleosides degradation|g__Escherichia.s__Escherichia_coli	0	0.000304	0.00027844	0	0.00051664	0	0	0	0	0
PWY0-1296: purine ribonucleosides degradation|g__Eubacterium.s__Eubacterium_rectale	0.00042305	0	0	0	0.00075952	0.00023659	0	0	0.00160824	0
PWY0-1296: purine ribonucleosides degradation|g__Faecalibacterium.s__Faecalibacterium_prausnitzii	0.00032008	0.00186332	0	0.00062587	0.00034528	0	0.00025432	0	0.0016412	0.0015912
PWY0-1296: purine ribonucleosides degradation|g__Fusobacterium.s__Fusobacterium_mortiferum	0	0	0.00029001	0	0	0	0	0	0	0
PWY0-1296: purine ribonucleosides degradation|g__Granulicatella.s__Granulicatella_elegans	0.00059443	0	0	0	0	0	0	0	0	0
PWY0-1296: purine ribonucleosides degradation|g__Lachnospiraceae_noname.s__Lachnospiraceae_bacterium_2_1_58FAA	0	0	0.00037073	0	0	0	0	0	0	0
PWY0-1296: purine ribonucleosides degradation|g__Megamonas.s__Megamonas_rupellensis	0	0	0	0	0	0	0.0007157	0	0	0
PWY0-1296: purine ribonucleosides degradation|g__Megasphaera.s__Megasphaera_elsdenii	0	0	0	0	0.00083632	0	0	0	0	0
PWY0-1296: purine ribonucleosides degradation|g__Mitsuokella.s__Mitsuokella_multacida	0	0	0	0	0.00040792	0	0.00173761	0	0	0
PWY0-1296: purine ribonucleosides degradation|g__Mycoplasma.s__Mycoplasma_hominis	0	0	0	0	0	0.00027289	0	0	0	0
PWY0-1296: purine ribonucleosides degradation|g__Roseburia.s__Roseburia_inulinivorans	0	0.00061182	0	0	0.00031356	0	0	0	0.00016272	0
PWY0-1296: purine ribonucleosides degradation|unclassified	0	0	0	0	0	0.0116616	0	0	0.0010619	0
PWY0-1297: superpathway of purine deoxyribonucleosides degradation|g__Blautia.s__Ruminococcus_gnavus	0.00381076	0	0.00142955	0	0	0	0	0	0	0
PWY0-1297: superpathway of purine deoxyribonucleosides degradation|g__Escherichia.s__Escherichia_coli	0	0	0.000312	0	0.00037864	0	0	0	0	0
PWY0-1297: superpathway of purine deoxyribonucleosides degradation|g__Lachnospiraceae_noname.s__Lachnospiraceae_bacterium_2_1_58FAA	0	0	0.00041027	0	0	0	0	0	0	0
PWY0-1319: CDP-diacylglycerol biosynthesis II|g__Anaerococcus.s__Anaerococcus_vaginalis	0	0.0003813	0	0	0	0	0	0	0	0
PWY0-1319: CDP-diacylglycerol biosynthesis II|g__Bacteroides.s__Bacteroides_fragilis	0	0	0.00033904	0	0	0	0	0	0	0.00388397
PWY0-1319: CDP-diacylglycerol biosynthesis II|g__Blautia.s__Ruminococcus_gnavus	0.00400588	0	0.00207134	0	0	0	0	0	0	0
PWY0-1319: CDP-diacylglycerol biosynthesis II|g__Blautia.s__Ruminococcus_torques	0	0	0	0	0	0	0.0006713	0	0	0
PWY0-1319: CDP-diacylglycerol biosynthesis II|g__Clostridium.s__Clostridium_nexile	0	0	0	0	0	0	0	0	0	0.00036513
PWY0-1319: CDP-diacylglycerol biosynthesis II|g__Dorea.s__Dorea_formicigenerans	0	0	0	0.00043313	0	0	0	0	0.00018517	0
PWY0-1319: CDP-diacylglycerol biosynthesis II|g__Dorea.s__Dorea_longicatena	0	0	0	0	0.00034403	0	0	0	0	0
PWY0-1319: CDP-diacylglycerol biosynthesis II|g__Escherichia.s__Escherichia_coli	0	0	0.00035616	0	0.00035519	0	0	0	0	0
PWY0-1319: CDP-diacylglycerol biosynthesis II|g__Eubacterium.s__Eubacterium_rectale	0.00026523	0	0	0.00064232	0	0	0	0	0.0005902	0.00032202
PWY0-1319: CDP-diacylglycerol biosynthesis II|g__Faecalibacterium.s__Faecalibacterium_prausnitzii	0.00026746	0.00065111	0	0.00090178	0.00037193	0	0.00044973	0	0.00187009	0.00130843
PWY0-1319: CDP-diacylglycerol biosynthesis II|g__Megamonas.s__Megamonas_hypermegale	0	0	0	0	0	0	0.00048918	0	0	0
PWY0-1319: CDP-diacylglycerol biosynthesis II|g__Mitsuokella.s__Mitsuokella_multacida	0	0	0	0	0	0	0.00174695	0	0	0
PWY0-1319: CDP-diacylglycerol biosynthesis II|g__Peptoniphilus.s__Peptoniphilus_lacrimalis	0	0	0	0	0	0.00042239	0	0	0	0

PWY0-1319: CDP-diacylglycerol biosynthesis II|g__Porphyromonas.s__Porphyromonas_asaccharolytica	0	0	0	0	0	0.00035547	0	0	0	0
PWY0-1319: CDP-diacylglycerol biosynthesis II|g__Prevotella.s__Prevotella_stercorea	0	0	0	0	0	0	0	0	0.00052378	0.00068208
PWY0-1319: CDP-diacylglycerol biosynthesis II|g__Roseburia.s__Roseburia_inulinivorans	0	0	0	0	0.00039173	0	0	0	0	0
PWY0-1338: polymyxin resistance	0	0.00018706	0.00029004	0.00024518	0	0	0	0	0	0
PWY0-1338: polymyxin resistance|g__Escherichia.s__Escherichia_coli	0	0	0.00029004	0	0	0	0	0	0	0
PWY0-1415: superpathway of heme biosynthesis from uroporphyrinogen-III	0.00050601	0	0.00028783	0.00052038	0	0	0	0	0	0.00046169
PWY0-1479: tRNA processing	0	0.00617462	0	0.00623357	0	0	0	0	0	0
PWY0-1533: methylphosphonate degradation I	0	0	0.00036418	0.00082623	0.00091689	0	0	0	0	0
PWY0-1533: methylphosphonate degradation I|g__Escherichia.s__Escherichia_coli	0	0	0.00036418	0.00080056	0.00091689	0	0	0	0	0
PWY0-1586: peptidoglycan maturation (meso-diaminopimelate containing)|g__Akkermansia.s__Akkermansia_muciniphila	0	0	0.00700898	0	0	0	0	0	0	0
PWY0-1586: peptidoglycan maturation (meso-diaminopimelate containing)|g__Escherichia.s__Escherichia_coli	0.00063161	0.00071457	0.00195815	0.00047734	0.00103405	0	0	0	0	0.00094516
PWY0-1586: peptidoglycan maturation (meso-diaminopimelate containing)|g__Faecalibacterium.s__Faecalibacterium_prausnitzii	0	0.00065148	0	0.00081725	0.00021674	0	0.00067176	0	0.00172698	0.0011997
PWY0-1586: peptidoglycan maturation (meso-diaminopimelate containing)|g__Megamonas.s__Megamonas_rupellensis	0	0	0	0	0	0	0.00087323	0	0	0
PWY0-162: superpathway of pyrimidine ribonucleotides de novo biosynthesis|unclassified	0	0	0	0	0	0	0	0	0.00111771	0
PWY0-166: superpathway of pyrimidine deoxyribonucleotides de novo biosynthesis (E. coli)|unclassified	0	0	0	0	0	0	0	0	0.00154521	0
PWY0-41: allantoin degradation IV (anaerobic)	0	0	0	0	0.00029363	0	0	0	0	0
PWY0-42: 2-methylcitrate cycle I	0	0	0	0.00037295	0	0	0	0	0	0
PWY0-781: aspartate superpathway	0.00188947	0.0018832	0.00148207	0.00612215	0.00287134	0	0.00293763	0	0.00458537	0.0010923
PWY0-845: superpathway of pyridoxal 5'-phosphate biosynthesis and salvage|g__Bacteroides.s__Bacteroides_fragilis	0.00759143	0	0	0	0	0	0	0	0	0
PWY0-845: superpathway of pyridoxal 5'-phosphate biosynthesis and salvage|unclassified	0	0.00303047	0	0	0	0	0	0	0	0
PWY0-862: (5Z)-dodec-5-enoate biosynthesis	0.00069478	0.00194035	0.00199821	0	0.00154444	0	0.00060268	0	0	0.00051513
PWY0-862: (5Z)-dodec-5-enoate biosynthesis|g__Escherichia.s__Escherichia_coli	0	0	0.00048685	0	0.00054789	0	0	0	0	0
PWY4FS-7: phosphatidylglycerol biosynthesis I (plastidic)|g__Escherichia.s__Escherichia_coli	0	0	0	0	0.00047217	0	0	0	0	0
PWY4FS-8: phosphatidylglycerol biosynthesis II (non-plastidic)|g__Escherichia.s__Escherichia_coli	0	0	0	0	0.00047217	0	0	0	0	0
PWY4LZ-257: superpathway of fermentation (Chlamydomonas reinhardtii)	0.00053127	0	0.00035953	0	0	0	0	0	0	0.00137991
PWY66-399: gluconeogenesis III	0.0005079	0	0.00071287	0.00134301	0.00126251	0	0	0	0.00192743	0.00119838
PWY66-400: glycolysis VI (metazoan)	0.00666786	0.00248607	0.00499947	0.00178269	0.00389471	0	0.00673805	0	0.0032494	0.0101683
PWY66-400: glycolysis VI (metazoan)|g__Megamonas.s__Megamonas_hypermegale	0	0	0	0	0	0	0.00165382	0	0	0
PWY66-422: D-galactose degradation V (Leloir pathway)|g__Erysipelotrichaceae_noname.s__Eubacterium_biforme	0	0.00030304	0	0	0	0	0.00019045	0	0	0.00022508
PWY66-422: D-galactose degradation V (Leloir pathway)|g__Escherichia.s__Escherichia_coli	0	0	0.00054323	0	0	0	0	0	0	0
PWY66-422: D-galactose degradation V (Leloir pathway)|g__Eubacterium.s__Eubacterium_rectale	0.00025142	0	0	0.00103068	0.00045597	0	0	0	0.00090089	0.00041766

PWY66-422: D-galactose degradation V (Leloir pathway)|g__Faecalibacterium.s__Faecalibacterium_prausnitzii	0.00024187	0.00125034	0	0.00074654	0.00051221	0	0.00063836	0	0.00171059	0.00135029
PWY66-422: D-galactose degradation V (Leloir pathway)|g__Fusobacterium.s__Fusobacterium_gonidiaformans	0	0	0	0	0	0	0	0	0	0.00056366
PWY66-422: D-galactose degradation V (Leloir pathway)|g__Megamonas.s__Megamonas_rupellensis	0	0	0	0	0	0	0.00046486	0	0	0
PWY66-422: D-galactose degradation V (Leloir pathway)|g__Mitsuokella.s__Mitsuokella_multacida	0	0	0	0.00077125	0.00107337	0	0.00166227	0	0	0
PWYG-321: mycolate biosynthesis	0.00080939	0.00199835	0	0	0.00172755	0	0.00070347	0	0	0.00061673
PYRIDNUCSAL-PWY: NAD salvage pathway I	0.00043442	0	0.00070392	0.00211688	0.00095117	0	0	0	0.00198536	0.00141644
PYRIDNUCSAL-PWY: NAD salvage pathway I|g__Escherichia.s__Escherichia_coli	0.00020856	0	0.00042415	0	0	0	0	0	0	0
PYRIDNUCSYN-PWY: NAD biosynthesis I (from aspartate)|g__Megamonas.s__Megamonas_rupellensis	0	0	0	0	0	0	0.00073695	0	0	0
PYRIDNUCSYN-PWY: NAD biosynthesis I (from aspartate)|g__Prevotella.s__Prevotella_buccalis	0	0	0	0	0	0	0	0	0.00076755	0
PYRIDNUCSYN-PWY: NAD biosynthesis I (from aspartate)|unclassified	0	0.00104567	0	0	0	0	0.00187809	0	0	0
PYRIDOXSYN-PWY: pyridoxal 5'-phosphate biosynthesis I|g__Bacteroides.s__Bacteroides_fragilis	0.00754177	0	0	0	0	0	0	0	0	0
PYRIDOXSYN-PWY: pyridoxal 5'-phosphate biosynthesis I|unclassified	0	0.00474463	0	0	0	0	0	0	0	0
REDCITCYC: TCA cycle VIII (helicobacter)	0	0.0007428	0.00103759	0.00126363	0.00037951	0	0	0	0	0.00058821
RHAMCAT-PWY: L-rhamnose degradation I	0.00156009	0.00165923	0.00170836	0	0.00131236	0	0.00452232	0	0.00156777	0.00163699
RHAMCAT-PWY: L-rhamnose degradation I|g__Blautia.s__Ruminococcus_torques	0	0	0	0	0	0	0.00044757	0	0	0
RHAMCAT-PWY: L-rhamnose degradation I|g__Clostridium.s__Clostridium_hathewayi	0	0	0	0	0	0	0	0	0	0.00085756
RHAMCAT-PWY: L-rhamnose degradation I|g__Escherichia.s__Escherichia_coli	0	0.00059023	0.00102194	0	0.00078832	0	0	0	0	0
RHAMCAT-PWY: L-rhamnose degradation I|g__Faecalibacterium.s__Faecalibacterium_prausnitzii	0.00015183	0	0	0	0	0	0.00037946	0	0.00052864	0.00116419
RIBOSYN2-PWY: flavin biosynthesis I (bacteria and plants)|g__Escherichia.s__Escherichia_coli	0.00022667	0	0.00077815	0	0	0	0	0	0	0
SALVADEHYPOX-PWY: adenosine nucleotides degradation II	0.00145036	0.00133813	0.00135639	0.00101081	0.00152618	0.00138022	0.00087399	0	0.00100318	0.00129855
SALVADEHYPOX-PWY: adenosine nucleotides degradation II|g__Blautia.s__Ruminococcus_torques	0	0	0	0	0	0	0.00060335	0	0	0
SALVADEHYPOX-PWY: adenosine nucleotides degradation II|g__Escherichia.s__Escherichia_coli	0	0.00017642	0.00080057	0.00056396	0.00074748	0	0	0	0	0
SER-GLYSYN-PWY: superpathway of L-serine and glycine biosynthesis I|g__Blautia.s__Ruminococcus_torques	0	0	0	0	0	0	0.00057655	0	0	0
SER-GLYSYN-PWY: superpathway of L-serine and glycine biosynthesis I|g__Campylobacter.s__Campylobacter_hominis	0	0	0	0	0	0	0.00027545	0	0	0
SER-GLYSYN-PWY: superpathway of L-serine and glycine biosynthesis I|g__Dorea.s__Dorea_formicigenerans	0	0	0	0.00069818	0	0	0	0	0	0
SER-GLYSYN-PWY: superpathway of L-serine and glycine biosynthesis I|g__Escherichia.s__Escherichia_coli	0.0001294	0	0.00044846	0	0.00030618	0	0	0	0	0
SER-GLYSYN-PWY: superpathway of L-serine and glycine biosynthesis I|g__Faecalibacterium.s__Faecalibacterium_prausnitzii	0	0.00102958	0	0	0.00064066	0	0.00055096	0	0.00140339	0.0005741
SER-GLYSYN-PWY: superpathway of L-serine and glycine biosynthesis I|g__Mitsuokella.s__Mitsuokella_multacida	0	0.00070742	0	0.00097625	0.00064762	0	0.00152862	0	0	0
SER-GLYSYN-PWY: superpathway of L-serine and glycine biosynthesis I|g__Prevotella.s__Prevotella_disiens	0.00039471	0	0	0	0	0	0	0	0	0.00021229
SER-GLYSYN-PWY: superpathway of L-serine and glycine biosynthesis I|g__Ruminococcus.s__Ruminococcus_bromii	0	0	0	0	0	0	0	0	0	0.00025916
SER-GLYSYN-PWY: superpathway of L-serine and glycine biosynthesis I|unclassified	0	0	0	0	0	0	0	0	0.00083249	0
SO4ASSIM-PWY: sulfate reduction I (assimilatory)	0.00031343	0.00141824	0.00518969	0.00323057	0.00109217	0	0	0	0.0026332	0.00508318

SO4ASSIM-PWY: sulfate reduction I (assimilatory)|g__Akkermansia.s__Akkermansia_muciniphila	0	0	0.00405357	0	0	0	0	0	0	0
SO4ASSIM-PWY: sulfate reduction I (assimilatory)|g__Escherichia.s__Escherichia_coli	0.00021543	0	0.00048693	0.00083629	0.0008611	0	0	0	0	0.00044678
SULFATE-CYS-PWY: superpathway of sulfate assimilation and cysteine biosynthesis	0.00062447	0.00157138	0.00256765	0.00252047	0.00147403	0	0	0	0.00377247	0.00267447
SULFATE-CYS-PWY: superpathway of sulfate assimilation and cysteine biosynthesis|g__Escherichia.s__Escherichia_coli	0	0	0.00034696	0	0.00036968	0	0	0	0	0
TCA-GLYOX-BYPASS: superpathway of glyoxylate bypass and TCA	0.00038691	0.00085886	0.00137041	0.00172066	0.00130843	0	0	0	0	0.00091837
TCA-GLYOX-BYPASS: superpathway of glyoxylate bypass and TCA|g__Escherichia.s__Escherichia_coli	0	0	0.00082562	0.0005112	0.00045391	0	0	0	0	0
TCA: TCA cycle I (prokaryotic)|g__Escherichia.s__Escherichia_coli	0	0	0.00084602	0.00059061	0.00049217	0	0	0	0	0
THISYN-PWY: superpathway of thiamin diphosphate biosynthesis I	0.00522709	0	0.0027714	0.0032474	0.00463933	0	0	0	0	0.00222445
THISYNARA-PWY: superpathway of thiamin diphosphate biosynthesis III (eukaryotes)|g__Blautia.s__Ruminococcus_torques	0	0	0	0	0	0	0.00044785	0	0	0
THISYNARA-PWY: superpathway of thiamin diphosphate biosynthesis III (eukaryotes)|g__Faecalibacterium.s__Faecalibacterium_prausnitzii	0	0	0	0	0	0	0	0	0.00137286	0.00045767
THISYNARA-PWY: superpathway of thiamin diphosphate biosynthesis III (eukaryotes)|g__Fusobacterium.s__Fusobacterium_gonidiaformans	0	0	0	0	0	0	0	0	0	0.00055818
THISYNARA-PWY: superpathway of thiamin diphosphate biosynthesis III (eukaryotes)|g__Peptoniphilus.s__Peptoniphilus_duerdenii	0	0	0	0	0	0.00059004	0	0	0	0
THRESYN-PWY: superpathway of L-threonine biosynthesis|g__Bifidobacterium.s__Bifidobacterium_adolescentis	0.00217213	0	0.00033969	0.00428367	0.00897797	0	0.00084	0	0.0025782	0
THRESYN-PWY: superpathway of L-threonine biosynthesis|g__Bifidobacterium.s__Bifidobacterium_catenulatum	0	0	0	0	0	0	0	0	0.00041828	0
THRESYN-PWY: superpathway of L-threonine biosynthesis|g__Blautia.s__Ruminococcus_torques	0	0	0	0	0	0	0.00079736	0	0	0
THRESYN-PWY: superpathway of L-threonine biosynthesis|g__Erysipelotrichaceae_noname.s__Eubacterium_biforme	0	0	0	0	0	0	0.00033306	0	0	0
THRESYN-PWY: superpathway of L-threonine biosynthesis|g__Escherichia.s__Escherichia_coli	0	0	0.00065707	0	0.00035012	0	0	0	0	0.00035738
THRESYN-PWY: superpathway of L-threonine biosynthesis|g__Faecalibacterium.s__Faecalibacterium_prausnitzii	0.00016864	0.00122128	0	0.00060302	0.00026446	0	0.00082002	0	0.00178999	0.0012798
THRESYN-PWY: superpathway of L-threonine biosynthesis|g__Mitsuokella.s__Mitsuokella_multacida	0	0	0	0.00044114	0.00057137	0	0.00085925	0	0	0
THRESYN-PWY: superpathway of L-threonine biosynthesis|g__Peptoniphilus.s__Peptoniphilus_lacrimalis	0	0	0	0	0	0.00046405	0	0	0	0
THRESYN-PWY: superpathway of L-threonine biosynthesis|unclassified	0	0	0	0	0	0.00708561	0	0	0	0
TRNA-CHARGING-PWY: tRNA charging|unclassified	0	0.00493053	0	0	0	0	0	0	0.00159011	0
TRPSYN-PWY: L-tryptophan biosynthesis|g__Bacteroides.s__Bacteroides_fragilis	0.00639717	0	0.00085656	0	0	0	0	0	0	0.00431473
TRPSYN-PWY: L-tryptophan biosynthesis|g__Escherichia.s__Escherichia_coli	0	0	0.00047284	0.00029299	0.00017985	0	0	0	0	0
UBISYN-PWY: superpathway of ubiquinol-8 biosynthesis (prokaryotic)	0	0	0	0	0.0005092	0	0	0	0	0
UDPNAGSYN-PWY: UDP-N-acetyl-D-glucosamine biosynthesis I|g__Bifidobacterium.s__Bifidobacterium_adolescentis	0.00105483	0	0	0.00108555	0.00234159	0	0.00081757	0	0.00066097	0
UDPNAGSYN-PWY: UDP-N-acetyl-D-glucosamine biosynthesis I|g__Bifidobacterium.s__Bifidobacterium_catenulatum	0	0	0	0	0	0	0	0	0.0006311	0
UDPNAGSYN-PWY: UDP-N-acetyl-D-glucosamine biosynthesis I|g__Bifidobacterium.s__Bifidobacterium_longum	0.0003691	0	0	0.00036565	0.00035811	0	0	0	0	0

UDPNAGSYN-PWY: UDP-N-acetyl-D-glucosamine biosynthesis Ilg__Campylobacter.s__Campylobacter_hominis	0	0	0	0	0	0	0.0001531	0	0	0
UDPNAGSYN-PWY: UDP-N-acetyl-D-glucosamine biosynthesis Ilg__Escherichia.s__Escherichia_coli	0.00012742	0	0.00082329	0.00057324	0.00083999	0	0	0	0	0.00020386
URDEGR-PWY: superpathway of allantoin degradation in plants	0	0	0	0	0.0002545	0	0	0	0	0
VALSYN-PWY: L-valine biosynthesis|g__Actinomyces.s__Actinomyces_turicensis	0	0	0	0	0	0.00119669	0	0	0.00073136	0
VALSYN-PWY: L-valine biosynthesis|g__Akkermansia.s__Akkermansia_muciniphila	0	0	0.00660927	0	0	0	0	0	0	0
VALSYN-PWY: L-valine biosynthesis|g__Bacteroides.s__Bacteroides_vulgatus	0	0	0.00045913	0.00027739	0.00071489	0.00060547	0	0	9.72E-05	0
VALSYN-PWY: L-valine biosynthesis|g__Bifidobacterium.s__Bifidobacterium_adolescentis	0.00215929	0	0.00043379	0.00420073	0.00673951	0	0.00103908	0	0.00165851	0
VALSYN-PWY: L-valine biosynthesis|g__Bifidobacterium.s__Bifidobacterium_catenulatum	0	0	0	0	0	0	0	0	0.00043579	0
VALSYN-PWY: L-valine biosynthesis|g__Bifidobacterium.s__Bifidobacterium_longum	0.00025461	0	0	0.00040717	0.00022459	0	0	0	0	0
VALSYN-PWY: L-valine biosynthesis|g__Blautia.s__Ruminococcus_obeum	0	0	0	0	0	0	0	0	0.00022276	0.0001711
VALSYN-PWY: L-valine biosynthesis|g__Blautia.s__Ruminococcus_torques	0	0	0	0	0	0	0.00096224	0	0	0
VALSYN-PWY: L-valine biosynthesis|g__Butyrivibrio.s__Butyrivibrio_crossotus	0	0.00027656	0	0	0	0	0	0	0	0
VALSYN-PWY: L-valine biosynthesis|g__Campylobacter.s__Campylobacter_hominis	0	0.00037023	0	0	0	0	0.00015107	0	0	0
VALSYN-PWY: L-valine biosynthesis|g__Catenibacterium.s__Catenibacterium_mitsuokai	0.00050043	0.0005732	0	0	0.00074546	0	0.00084998	0	0	0
VALSYN-PWY: L-valine biosynthesis|g__Clostridium.s__Clostridium_hathewayi	0	0	0	0	0	0	0	0	0	0.00061863
VALSYN-PWY: L-valine biosynthesis|g__Clostridium.s__Clostridium_nexile	0	0	0	0	0	0.00045781	0	0	0	0.00147809
VALSYN-PWY: L-valine biosynthesis|g__Desulfovibrio.s__Desulfovibrio_piger	0	0	0.00024669	0	0	0	0	0	0	0
VALSYN-PWY: L-valine biosynthesis|g__Dorea.s__Dorea_formicigenerans	0.00018524	0	0	0	0	0	0	0	0.00016854	0
VALSYN-PWY: L-valine biosynthesis|g__Dorea.s__Dorea_longicatena	0.00011058	0	0	0	0	0	0	0	0	0
VALSYN-PWY: L-valine biosynthesis|g__Erysipelotrichaceae_noname.s__Erysipelotrichaceae_bacterium_6_1_45	0	0	0.00023405	0	0	0	0	0	0	0
VALSYN-PWY: L-valine biosynthesis|g__Escherichia.s__Escherichia_coli	0	0	0.00064918	0.00051628	0.00082344	0	0	0	0	0.00055537
VALSYN-PWY: L-valine biosynthesis|g__Eubacterium.s__Eubacterium_rectale	0.00024424	0	0.00013449	0.00164565	0.00036744	0	0	0	0.00115241	0
VALSYN-PWY: L-valine biosynthesis|g__Eubacterium.s__Eubacterium_siraeum	0	0.00025101	0	0	0	0	0	0	0	0
VALSYN-PWY: L-valine biosynthesis|g__Faecalibacterium.s__Faecalibacterium_prausnitzii	0.0002131	0.00303228	0	0.00093488	0.00074141	0	0.00093202	0	0.00365944	0.00251559
VALSYN-PWY: L-valine biosynthesis|g__Megamonas.s__Megamonas_hypermegale	0	0	0	0	0	0	0.00040219	0	0	0
VALSYN-PWY: L-valine biosynthesis|g__Megamonas.s__Megamonas_rupellensis	0	0	0	0	0	0	0.0004512	0	0	0
VALSYN-PWY: L-valine biosynthesis|g__Methanobrevibacter.s__Methanobrevibacter_smithii	0	0	0.00016621	0	0	0	0	0	0	0
VALSYN-PWY: L-valine biosynthesis|g__Mitsuokella.s__Mitsuokella_multacida	0	0.00043826	0	0.00117596	0.00073457	0	0.00111117	0	0	0
VALSYN-PWY: L-valine biosynthesis|g__Phascolarctobacterium.s__Phascolarctobacterium_succinatutens	0	0	0	0	0	0	0	0	0	0.00014095

VALSYN-PWY: L-valine biosynthesis|g__Roseburia.s__Roseburia_inulinivorans	0	0	0	0	0.00047516	0	0	0	0	0
VALSYN-PWY: L-valine biosynthesis|g__Ruminococcus.s__Ruminococcus_bromii	0	0	0	0.00044185	0	0	0	0	0	0.00040023
VALSYN-PWY: L-valine biosynthesis|g__Streptococcus.s__Streptococcus_salivarius	0	0	0	0	0	0	0	0	0.00019146	0
VALSYN-PWY: L-valine biosynthesis|unclassified	0	0.00822518	0	0.00483489	0	0.0131769	0.00337359	0	0.00147156	0

Supplementary Table 5. List of significant pathways greater in abundance in the endocervical site for *Ct* positive compared to *Ct* negative groups.

Sl. No.	List of significant pathways in the endocervical site for *Ct* positive and negative groups.
1	1CMET2-PWY: N10-formyl-tetrahydrofolate biosynthesis
	ASPASN-PWY: superpathway of L-aspartate and L-asparagine biosynthesis|g__Prevotella.s__Prevotella_timonensis
	Mean of negative C samples: 0
	Mean of positive C samples: 0.000207974133333333
	Wilcoxon rank sum test with continuity correction
	0.0398626
2	CALVIN-PWY: Calvin-Benson-Bassham cycle
	Mean of negative C samples: 0.00450651266666667
	Mean of positive C samples: 0.00993423533333333
	Wilcoxon rank sum test with continuity correction
	0.01604828
3	COA-PWY-1: coenzyme A biosynthesis II (mammalian)|g__Peptoniphilus.s__Peptoniphilus_lacrimalis
	Mean of negative C samples: 0
	Mean of positive C samples: 0.0001673434
	Wilcoxon rank sum test with continuity correction
	0.0398626
4	COA-PWY-1: coenzyme A biosynthesis II (mammalian)|g__Porphyromonas.s__Porphyromonas_uenonis
	Mean of negative C samples: 0
	Mean of positive C samples: 0.0003306706
	Wilcoxon rank sum test with continuity correction
	0.0398626
5	COA-PWY-1: coenzyme A biosynthesis II (mammalian)|g__Prevotella.s__Prevotella_amnii
	Mean of negative C samples: 0
	Mean of positive C samples: 0.00118434066666667
	Wilcoxon rank sum test with continuity correction
	0.0398626
6	COA-PWY: coenzyme A biosynthesis I|g__Clostridiales_noname.s__Clostridiales_genomosp_BVAB3
	Mean of negative C samples: 0
	Mean of positive C samples: 0.00718997266666667
	Wilcoxon rank sum test with continuity correction
	0.01920716
7	COA-PWY: coenzyme A biosynthesis I|g__Peptoniphilus.s__Peptoniphilus_lacrimalis
	Mean of negative C samples: 0
	Mean of positive C samples: 0.000151556533333333
	Wilcoxon rank sum test with continuity correction
	0.01920716
8	COA-PWY: coenzyme A biosynthesis I|g__Porphyromonas.s__Porphyromonas_uenonis
	Mean of negative C samples: 0
	Mean of positive C samples: 0.0003845866

	Wilcoxon rank sum test with continuity correction
	0.0398626
9	COA-PWY: coenzyme A biosynthesis I|g__Prevotella.s__Prevotella_amnii
	Mean of negative C samples: 0
	Mean of positive C samples: 0.00207121066666667
	Wilcoxon rank sum test with continuity correction
	0.009048307
10	DTDPRHAMSYN-PWY: dTDP-L-rhamnose biosynthesis I
	Mean of negative C samples: 0.006882602
	Mean of positive C samples: 0.0245929126666667
	Wilcoxon rank sum test with continuity correction
	0.02235376
11	DTDPRHAMSYN-PWY: dTDP-L-rhamnose biosynthesis I|g__Porphyromonas.s__Porphyromonas_uenonis
	Mean of negative C samples: 0
	Mean of positive C samples: 0.000588746133333333
	Wilcoxon rank sum test with continuity correction
	0.01920716
12	DTDPRHAMSYN-PWY: dTDP-L-rhamnose biosynthesis I|g__Prevotella.s__Prevotella_amnii
	Mean of negative C samples: 0.00022056
	Mean of positive C samples: 0.00168153266666667
	Wilcoxon rank sum test with continuity correction
	0.03748188
13	DTDPRHAMSYN-PWY: dTDP-L-rhamnose biosynthesis I|g__Prevotella.s__Prevotella_timonensis
	Mean of negative C samples: 0
	Mean of positive C samples: 0.000305991133333333
	Wilcoxon rank sum test with continuity correction
	0.0398626
14	HISDEG-PWY: L-histidine degradation I|g__Peptoniphilus.s__Peptoniphilus_lacrimalis
	Mean of negative C samples: 0
	Mean of positive C samples: 0.000135893
	Wilcoxon rank sum test with continuity correction
	0.01920716
15	PANTO-PWY: phosphopantothenate biosynthesis I
	Mean of negative C samples: 0.00634068133333333
	Mean of positive C samples: 0.02190305
	Wilcoxon rank sum test with continuity correction
	0.007668517
16	PANTO-PWY: phosphopantothenate biosynthesis I|g__Porphyromonas.s__Porphyromonas_uenonis
	Mean of negative C samples: 0
	Mean of positive C samples: 0.000460947733333333
	Wilcoxon rank sum test with continuity correction
	0.01920716
17	PANTOSYN-PWY: pantothenate and coenzyme A biosynthesis I

	Mean of negative C samples: 0.00419575733333333
	Mean of positive C samples: 0.009325818
	Wilcoxon rank sum test with continuity correction
	0.04576236
18	PANTOSYN-PWY: pantothenate and coenzyme A biosynthesis I|g__Porphyromonas.s__Porphyromonas_uenonis
	Mean of negative C samples: 0
	Mean of positive C samples: 0.000358429466666667
	Wilcoxon rank sum test with continuity correction
	0.0398626
19	PEPTIDOGLYCANSYN-PWY: peptidoglycan biosynthesis I (meso-diaminopimelate containing)|g__Faecalibacterium.s__Faecalibacterium_prausnitzii
	Mean of negative C samples: 0
	Mean of positive C samples: 0.000251344133333333
	Wilcoxon rank sum test with continuity correction
	0.0398626
20	PWY-1042: glycolysis IV (plant cytosol)
	Mean of negative C samples: 0.00672810266666667
	Mean of positive C samples: 0.01184764
	Wilcoxon rank sum test with continuity correction
	0.02280408
21	PWY-1042: glycolysis IV (plant cytosol)|unclassified
	Mean of negative C samples: 0
	Mean of positive C samples: 0.00048177
	Wilcoxon rank sum test with continuity correction
	0.0398626
22	PWY-2942: L-lysine biosynthesis III
	Mean of negative C samples: 0.004925164
	Mean of positive C samples: 0.0210235826666667
	Wilcoxon rank sum test with continuity correction
	0.01525733
23	PWY-2942: L-lysine biosynthesis III|g__Faecalibacterium.s__Faecalibacterium_prausnitzii
	Mean of negative C samples: 0
	Mean of positive C samples: 0.000141157733333333
	Wilcoxon rank sum test with continuity correction
	0.0398626
24	PWY-2942: L-lysine biosynthesis III|g__Porphyromonas.s__Porphyromonas_uenonis
	Mean of negative C samples: 0
	Mean of positive C samples: 0.0003424068
	Wilcoxon rank sum test with continuity correction
	0.0398626
25	PWY-2942: L-lysine biosynthesis III|g__Prevotella.s__Prevotella_amnii
	Mean of negative C samples: 0
	Mean of positive C samples: 0.000971763333333333

	Wilcoxon rank sum test with continuity correction
	0.0398626
26	PWY-4242: pantothenate and coenzyme A biosynthesis III|g__Megasphaera.s__Megasphaera_genomosp_type_1
	Mean of negative C samples: 0
	Mean of positive C samples: 0.000787704666666667
	Wilcoxon rank sum test with continuity correction
	0.0398626
27	PWY-4242: pantothenate and coenzyme A biosynthesis III|g__Peptoniphilus.s__Peptoniphilus_lacrimalis
	Mean of negative C samples: 0
	Mean of positive C samples: 0.000123256066666667
	Wilcoxon rank sum test with continuity correction
	0.0398626
28	PWY-5667: CDP-diacylglycerol biosynthesis I|g__Peptostreptococcus.s__Peptostreptococcus_anaerobius
	Mean of negative C samples: 0
	Mean of positive C samples: 5.95804e-05
	Wilcoxon rank sum test with continuity correction
	0.0398626
29	PWY-5667: CDP-diacylglycerol biosynthesis I|g__Porphyromonas.s__Porphyromonas_uenonis
	Mean of negative C samples: 0
	Mean of positive C samples: 0.000364170133333333
	Wilcoxon rank sum test with continuity correction
	0.01920716
30	PWY-5686: UMP biosynthesis|g__Collinsella.s__Collinsella_aerofaciens
	Mean of negative C samples: 0
	Mean of positive C samples: 0.000164659933333333
	Wilcoxon rank sum test with continuity correction
	0.0398626
31	PWY-5686: UMP biosynthesis|g__Porphyromonas.s__Porphyromonas_uenonis
	Mean of negative C samples: 0
	Mean of positive C samples: 0.000405925933333333
	Wilcoxon rank sum test with continuity correction
	0.01920716
32	PWY-5695: urate biosynthesis/inosine 5'-phosphate degradation
	Mean of negative C samples: 0.00355311866666667
	Mean of positive C samples: 0.010814502
	Wilcoxon rank sum test with continuity correction
	0.01440524
33	PWY-6123: inosine-5'-phosphate biosynthesis I|unclassified
	Mean of negative C samples: 0
	Mean of positive C samples: 0.000228310933333333
	Wilcoxon rank sum test with continuity correction
	0.0398626
34	PWY-6151: S-adenosyl-L-methionine cycle I|g__Porphyromonas.s__Porphyromonas_uenonis

	Mean of negative C samples: 0
	Mean of positive C samples: 0.000352874666666667
	Wilcoxon rank sum test with continuity correction
	0.0398626
35	PWY-6151: S-adenosyl-L-methionine cycle I|g__Prevotella.s__Prevotella_amnii
	Mean of negative C samples: 0
	Mean of positive C samples: 0.001342314
	Wilcoxon rank sum test with continuity correction
	0.01920716
36	PWY-6385: peptidoglycan biosynthesis III (mycobacteria)|g__Gardnerella.s__Gardnerella_vaginalis
	Mean of negative C samples: 0
	Mean of positive C samples: 0.000367912
	Wilcoxon rank sum test with continuity correction
	0.0398626
37	PWY-6386: UDP-N-acetylmuramoyl-pentapeptide biosynthesis II (lysine-containing)|g__Faecalibacterium.s__Faecalibacterium_prausnitzii
	Mean of negative C samples: 0
	Mean of positive C samples: 0.000267089266666667
	Wilcoxon rank sum test with continuity correction
	0.0398626
38	PWY-6386: UDP-N-acetylmuramoyl-pentapeptide biosynthesis II (lysine-containing)|g__Prevotella.s__Prevotella_amnii
	Mean of negative C samples: 0.00010408
	Mean of positive C samples: 0.00187466466666667
	Wilcoxon rank sum test with continuity correction
	0.03288102
39	PWY-6387: UDP-N-acetylmuramoyl-pentapeptide biosynthesis I (meso-diaminopimelate containing)|g__Prevotella.s__Prevotella_amnii
	Mean of negative C samples: 9.7692e-05
	Mean of positive C samples: 0.00162623066666667
	Wilcoxon rank sum test with continuity correction
	0.03288102
40	PWY-6609: adenine and adenosine salvage III
	Mean of negative C samples: 0.00244062853333333
	Mean of positive C samples: 0.016935864
	Wilcoxon rank sum test with continuity correction
	0.005719678
41	PWY-6609: adenine and adenosine salvage III|g__Clostridiales_noname.s__Clostridiales_genomosp_BVAB3
	Mean of negative C samples: 0
	Mean of positive C samples: 0.000713626066666667
	Wilcoxon rank sum test with continuity correction
	0.01920716
42	PWY-6609: adenine and adenosine salvage III|g__Porphyromonas.s__Porphyromonas_uenonis
	Mean of negative C samples: 0

	Mean of positive C samples: 0.0006457888
	Wilcoxon rank sum test with continuity correction
	0.01920716
43	PWY-6609: adenine and adenosine salvage III|g__Prevotella.s__Prevotella_timonensis
	Mean of negative C samples: 0
	Mean of positive C samples: 0.0002191374
	Wilcoxon rank sum test with continuity correction
	0.0398626
44	PWY-6703: preQ0 biosynthesis|g__Porphyromonas.s__Porphyromonas_uenonis
	Mean of negative C samples: 0
	Mean of positive C samples: 0.0004239736
	Wilcoxon rank sum test with continuity correction
	0.01920716
45	PWY-6737: starch degradation V|g__Erysipelotrichaceae_noname.s__Eubacterium_biforme
	Mean of negative C samples: 0
	Mean of positive C samples: 3.65846e-05
	Wilcoxon rank sum test with continuity correction
	0.0398626
46	PWY-7211: superpathway of pyrimidine deoxyribonucleotides de novo biosynthesis
	Mean of negative C samples: 0
	Mean of positive C samples: 0.000338932933333333
	Wilcoxon rank sum test with continuity correction
	0.0398626
47	PWY-7221: guanosine ribonucleotides de novo biosynthesis|g__Clostridiales_noname.s__Clostridiales_genomosp_BVAB3
	Mean of negative C samples: 0
	Mean of positive C samples: 0.002169382
	Wilcoxon rank sum test with continuity correction
	0.0398626
48	PWY-7221: guanosine ribonucleotides de novo biosynthesis|g__Porphyromonas.s__Porphyromonas_uenonis
	Mean of negative C samples: 0
	Mean of positive C samples: 0.0004247142
	Wilcoxon rank sum test with continuity correction
	0.01920716
49	PWY-7221: guanosine ribonucleotides de novo biosynthesis|g__Prevotella.s__Prevotella_amnii
	Mean of negative C samples: 0.000127599333333333
	Mean of positive C samples: 0.00356307866666667
	Wilcoxon rank sum test with continuity correction
	0.03288102
50	PWY-7221: guanosine ribonucleotides de novo biosynthesis|g__Prevotella.s__Prevotella_timonensis
	Mean of negative C samples: 0
	Mean of positive C samples: 0.000483203066666667
	Wilcoxon rank sum test with continuity correction

	0.009048307
51	PWY-7357: thiamin formation from pyrithiamine and oxythiamine (yeast)|g__Faecalibacterium.s__Faecalibacterium_prausnitzii
	Mean of negative C samples: 0
	Mean of positive C samples: 0.0001442798
	Wilcoxon rank sum test with continuity correction
	0.0398626
52	PWY0-1319: CDP-diacylglycerol biosynthesis II|g__Peptostreptococcus.s__Peptostreptococcus_anaerobius
	Mean of negative C samples: 0
	Mean of positive C samples: 5.95804e-05
	Wilcoxon rank sum test with continuity correction
	0.0398626
53	PWY0-1319: CDP-diacylglycerol biosynthesis II|g__Porphyromonas.s__Porphyromonas_uenonis
	Mean of negative C samples: 0
	Mean of positive C samples: 0.000364170133333333
	Wilcoxon rank sum test with continuity correction
	0.01920716
54	PYRIDNUCSYN-PWY: NAD biosynthesis I (from aspartate)|g__Porphyromonas.s__Porphyromonas_asaccharolytica
	Mean of negative C samples: 0
	Mean of positive C samples: 0.0001051312
	Wilcoxon rank sum test with continuity correction
	0.0398626
55	PYRIDNUCSYN-PWY: NAD biosynthesis I (from aspartate)|g__Porphyromonas.s__Porphyromonas_uenonis
	Mean of negative C samples: 0
	Mean of positive C samples: 0.000305497133333333
	Wilcoxon rank sum test with continuity correction
	0.01920716
56	SER-GLYSYN-PWY: superpathway of L-serine and glycine biosynthesis I
	Mean of negative C samples: 0.00179109866666667
	Mean of positive C samples: 0.005480758
	Wilcoxon rank sum test with continuity correction
	0.01367059
57	THISYNARA-PWY: superpathway of thiamin diphosphate biosynthesis III (eukaryotes)
	Mean of negative C samples: 0.002263288
	Mean of positive C samples: 0.00748402533333333
	Wilcoxon rank sum test with continuity correction
	0.03140906

Supplementary Table 6. List of significant pathways greater in abundance in the vaginal site for *Ct* positive compared to *Ct* negative groups.

**Sl. No.**	**List of significant pathways in the vaginal site for Ct positive and negative groups.**
1	COA-PWY: coenzyme A biosynthesis I|g__Prevotella.s__Prevotella_amnii
	Mean of negative V samples: 0
	Mean of positive V samples: 0.00482089
	Wilcoxon rank sum test with continuity correction
	0.03600283
2	PANTO-PWY: phosphopantothenate biosynthesis I
	Mean of negative V samples: 0.0075926
	Mean of positive V samples: 0.02836558
	Wilcoxon rank sum test with continuity correction
	0.01722682
3	PWY-2942: L-lysine biosynthesis III
	Mean of negative V samples: 0.000835652
	Mean of positive V samples: 0.01342348
	Wilcoxon rank sum test with continuity correction
	0.02245429
4	PWY-5695: urate biosynthesis/inosine 5'-phosphate degradation
	Mean of negative V samples: 0.002564818
	Mean of positive V samples: 0.019471752
	Wilcoxon rank sum test with continuity correction
	0.01722682
5	PWY-6270: isoprene biosynthesis I
	Mean of negative V samples: 0
	Mean of positive V samples: 0.005477904
	Wilcoxon rank sum test with continuity correction
	0.03600283
6	PWY-6609: adenine and adenosine salvage III
	Mean of negative V samples: 0.001881482
	Mean of positive V samples: 0.03648411
	Wilcoxon rank sum test with continuity correction
	0.01000409
7	PWY-7219: adenosine ribonucleotides de novo biosynthesis|g__Prevotella.s__Prevotella_amnii
	Mean of negative V samples: 0.00040457
	Mean of positive V samples: 0.008595214
	Wilcoxon rank sum test with continuity correction
	0.02245429
8	PWY-7221: guanosine ribonucleotides de novo biosynthesis|g__Clostridiales_noname.s__Clostridiales_genomosp_BVAB3
	Mean of negative V samples: 0
	Mean of positive V samples: 0.006508146
	Wilcoxon rank sum test with continuity correction

	0.03600283
9	PWY-7560: methylerythritol phosphate pathway II
	Mean of negative V samples: 0
	Mean of positive V samples: 0.005117306
	Wilcoxon rank sum test with continuity correction
	0.03600283
10	PYRIDNUCSYN-PWY: NAD biosynthesis I (from aspartate)
	Mean of negative V samples: 0.000976034
	Mean of positive V samples: 0.009138072
	Wilcoxon rank sum test with continuity correction
	0.03347764
11	SER-GLYSYN-PWY: superpathway of L-serine and glycine biosynthesis I
	Mean of negative V samples: 0.0009404
	Mean of positive V samples: 0.007829592
	Wilcoxon rank sum test with continuity correction
	0.03347764

Supplementary Table 7. List of significant pathways greater in abundance in the rectal site for *Ct* positive compared to *Ct* negative groups.

Sl. No.	List of significant pathways in the rectal site for *Ct* positive and negative groups.
1	CALVIN-PWY: Calvin-Benson-Bassham cycle
	Mean of negative R samples: 0.007751638
	Mean of positive R samples: 0.010358246
	Wilcoxon rank sum test with continuity correction
	0.01078587
2	COA-PWY: coenzyme A biosynthesis I|g__Peptoniphilus.s__Peptoniphilus_lacrimalis
	Mean of negative R samples: 0
	Mean of positive R samples: 0.0002876168
	Wilcoxon rank sum test with continuity correction
	0.03600283
3	DTDPRHAMSYN-PWY: dTDP-L-rhamnose biosynthesis I|g__Porphyromonas.s__Porphyromonas_uenonis
	Mean of negative R samples: 0
	Mean of positive R samples: 0.0010232544
	Wilcoxon rank sum test with continuity correction
	0.03600283
4	HSERMETANA-PWY: L-methionine biosynthesis III
	Mean of negative R samples: 0.00190689
	Mean of positive R samples: 0.003265976
	Wilcoxon rank sum test with continuity correction
	0.04734647
5	P164-PWY: purine nucleobases degradation I (anaerobic)
	Mean of negative R samples: 0.000217072
	Mean of positive R samples: 0.001716426
	Wilcoxon rank sum test with continuity correction
	0.03731959
6	PANTO-PWY: phosphopantothenate biosynthesis I|g__Porphyromonas.s__Porphyromonas_uenonis
	Mean of negative R samples: 0
	Mean of positive R samples: 0.0010095312
	Wilcoxon rank sum test with continuity correction
	0.03600283
7	PEPTIDOGLYCANSYN-PWY: peptidoglycan biosynthesis I (meso-diaminopimelate containing)|g__Faecalibacterium.s__Faecalibacterium_prausnitzii
	Mean of negative R samples: 0
	Mean of positive R samples: 0.0007540324
	Wilcoxon rank sum test with continuity correction
	0.03600283
8	PWY-1042: glycolysis IV (plant cytosol)
	Mean of negative R samples: 0.010036268
	Mean of positive R samples: 0.01420182
	Wilcoxon rank sum test with continuity correction
	0.00609289

9	PWY-1042: glycolysis IV (plant cytosol)|unclassified
	Mean of negative R samples: 0
	Mean of positive R samples: 0.00144531
	Wilcoxon rank sum test with continuity correction
	0.03600283
10	PWY-2942: L-lysine biosynthesis III|g__Faecalibacterium.s__Faecalibacterium_prausnitzii
	Mean of negative R samples: 0
	Mean of positive R samples: 0.0004234732
	Wilcoxon rank sum test with continuity correction
	0.03600283
11	PWY-4981: L-proline biosynthesis II (from arginine)
	Mean of negative R samples: 0.002988556
	Mean of positive R samples: 0.005296054
	Wilcoxon rank sum test with continuity correction
	0.0300514
12	PWY-5100: pyruvate fermentation to acetate and lactate II
	Mean of negative R samples: 0.005457302
	Mean of positive R samples: 0.012290354
	Wilcoxon rank sum test with continuity correction
	0.0300514
13	PWY-5659: GDP-mannose biosynthesis|g__Faecalibacterium.s__Faecalibacterium_prausnitzii
	Mean of negative R samples: 0
	Mean of positive R samples: 0.0005541712
	Wilcoxon rank sum test with continuity correction
	0.03600283
14	PWY-5667: CDP-diacylglycerol biosynthesis I|g__Porphyromonas.s__Porphyromonas_uenonis
	Mean of negative R samples: 0
	Mean of positive R samples: 0.0005641724
	Wilcoxon rank sum test with continuity correction
	0.03600283
15	PWY-5686: UMP biosynthesis
	Mean of negative R samples: 0.01558742
	Mean of positive R samples: 0.02269956
	Wilcoxon rank sum test with continuity correction
	0.04734647
16	PWY-5686: UMP biosynthesis|g__Collinsella.s__Collinsella_aerofaciens
	Mean of negative R samples: 0
	Mean of positive R samples: 0.0004939798
	Wilcoxon rank sum test with continuity correction
	0.03600283
19	PWY-5686: UMP biosynthesis|g__Porphyromonas.s__Porphyromonas_uenonis
	Mean of negative R samples: 0
	Mean of positive R samples: 0.0008550318

	Wilcoxon rank sum test with continuity correction
	0.03600283
20	PWY-6123: inosine-5'-phosphate biosynthesis I|unclassified
	Mean of negative R samples: 0
	Mean of positive R samples: 0.0006849328
	Wilcoxon rank sum test with continuity correction
	0.03600283
21	PWY-6386: UDP-N-acetylmuramoyl-pentapeptide biosynthesis II (lysine-containing)|g__Faecalibacterium.s__Faecalibacterium_prausnitzii
	Mean of negative R samples: 0
	Mean of positive R samples: 0.0008012678
	Wilcoxon rank sum test with continuity correction
	0.03600283
22	PWY-6595: superpathway of guanosine nucleotides degradation (plants)
	Mean of negative R samples: 0.0001131422
	Mean of positive R samples: 0.0004618166
	Wilcoxon rank sum test with continuity correction
	0.03731959
23	PWY-6609: adenine and adenosine salvage III
	Mean of negative R samples: 0.0054404036
	Mean of positive R samples: 0.011202802
	Wilcoxon rank sum test with continuity correction
	0.04734647
24	PWY-6609: adenine and adenosine salvage III|g__Porphyromonas.s__Porphyromonas_uenonis
	Mean of negative R samples: 0
	Mean of positive R samples: 0.0013676024
	Wilcoxon rank sum test with continuity correction
	0.03600283
25	PWY-6609: adenine and adenosine salvage III|g__Prevotella.s__Prevotella_timonensis
	Mean of negative R samples: 0
	Mean of positive R samples: 0.0006574122
	Wilcoxon rank sum test with continuity correction
	0.03600283
26	PWY-6703: preQ0 biosynthesis|g__Porphyromonas.s__Porphyromonas_uenonis
	Mean of negative R samples: 0
	Mean of positive R samples: 0.0007687068
	Wilcoxon rank sum test with continuity correction
	0.03600283
27	PWY-6737: starch degradation V|g__Erysipelotrichaceae_noname.s__Eubacterium_biforme
	Mean of negative R samples: 0
	Mean of positive R samples: 0.0001097538
	Wilcoxon rank sum test with continuity correction
	0.03600283
28	PWY-7199: pyrimidine deoxyribonucleosides salvage

	Mean of negative R samples: 0.002615446
	Mean of positive R samples: 0.004792488
	Wilcoxon rank sum test with continuity correction
	0.0300514
29	PWY-7211: superpathway of pyrimidine deoxyribonucleotides de novo biosynthesis
	Mean of negative R samples: 0
	Mean of positive R samples: 0.0010167988
	Wilcoxon rank sum test with continuity correction
	0.03600283
30	PWY-7219: adenosine ribonucleotides de novo biosynthesis
	Mean of negative R samples: 0.02347884
	Mean of positive R samples: 0.03448928
	Wilcoxon rank sum test with continuity correction
	0.04734647
31	PWY-7221: guanosine ribonucleotides de novo biosynthesis|g__Porphyromonas.s__Porphyromonas_uenonis
	Mean of negative R samples: 0
	Mean of positive R samples: 0.0009165326
	Wilcoxon rank sum test with continuity correction
	0.03600283
32	PWY-7221: guanosine ribonucleotides de novo biosynthesis|g__Prevotella.s__Prevotella_timonensis
	Mean of negative R samples: 0
	Mean of positive R samples: 0.0006964536
	Wilcoxon rank sum test with continuity correction
	0.03600283
33	PWY-7229: superpathway of adenosine nucleotides de novo biosynthesis I
	Mean of negative R samples: 0.00632311
	Mean of positive R samples: 0.010614516
	Wilcoxon rank sum test with continuity correction
	0.01835693
34	PWY-7357: thiamin formation from pyrithiamine and oxythiamine (yeast)|g__Faecalibacterium.s__Faecalibacterium_prausnitzii
	Mean of negative R samples: 0
	Mean of positive R samples: 0.0004328394
	Wilcoxon rank sum test with continuity correction
	0.03600283
35	PWY0-1319: CDP-diacylglycerol biosynthesis II|g__Porphyromonas.s__Porphyromonas_uenonis
	Mean of negative R samples: 0
	Mean of positive R samples: 0.0005641724
	Wilcoxon rank sum test with continuity correction
	0.03600283
36	PYRIDNUCSYN-PWY: NAD biosynthesis I (from aspartate)
	Mean of negative R samples: 0.004100136
	Mean of positive R samples: 0.006182278
	Wilcoxon rank sum test with continuity correction

	0.01835693
37	PYRIDNUCSYN-PWY: NAD biosynthesis I (from aspartate)|g__Porphyromonas.s__Porphyromonas_uenonis
	Mean of negative R samples: 0
	Mean of positive R samples: 0.0007084214
	Wilcoxon rank sum test with continuity correction
	0.03600283
38	THISYNARA-PWY: superpathway of thiamin diphosphate biosynthesis III (eukaryotes)
	Mean of negative R samples: 0.001803784
	Mean of positive R samples: 0.005104034
	Wilcoxon rank sum test with continuity correction
	0.00609289
